# Supplementary material for: Uremic Toxins and Vascular Calcification–Missing the Forest for All the Trees
Source: Toxins (Basel). 2020 Sep 29;12(10):624. doi: 10.3390/toxins12100624 (PMC7599869; doi:10.3390/toxins12100624)
Supplement: Supplementary file 1 [file toxins-12-00624-s001.pdf]

Supplementary Materials: Uremic toxins and vascular calcification – missing the forest for all the trees

Nikolas Rapp, Pieter Evenepoel, Peter Stenvinkel and Leon Schurgers

Table S1. Full table of the MM and their influence on calcification, as well as their effects on VC related processes in VSMC = increase, = decrease, — = tested, but no effect, ~ = unclear effect.

Uremic retention molecule	Calcification - in vitro	Calcification - in vivo	Calcification - Clinical studies	Osteogenesis	Oxidative stress	Inflammation	Apoptosis	Senescence	Proliferation	Migration	Atherosclerosis
Adiponectin	↓[1–3]<br>—[4]	↓[5,6]	↑[7–9]<br>↓[10,11]<br>—[12–14]	↓[1,6]		↓[15]	↓[3,5]		↓[16–23]<br>~[24]	↓[20,21,25,26]	↓[26,27]
Adrenomedullin	↓[28,29]	↓[28,30]		[30]	↓[31,32]				↑[33,34]<br>↓[35–41]	↓[42–44]	
alpha 1 microglubulin					↓[45]						
Atrial Natriuretic Peptide					↓[46,47]		↑[48]<br>↓[49]		↑[47,50–64]	↓[50,65,66]	↓[62]
Basic fibroblast growth factor					↑[67]		↓[68–72]		↑[72–88]<br>↓[89]	↑[67,78,79,82, 85,90–99]	↑[88]<br>↓[100]
beta trace protein											
Calcitonin											
Calcitonin gene-related peptide					↓[101,102]	↓[103]	↑[104]<br>↓[101,105]		↑[106]<br>↓[39,102,106–112]	↓[102,113]	
Cholecystokinin						↓[114]					
Clara cell protein (CC16)											
Complement Factor D											
Cystatin C						↑[115]	↑[115]			↓[116]	↑[117]<br>↓[118]

Degranulation Inhibiting Protein I											
Delta-sleep Inducing Peptide											
Delta-sleep inducing peptide											
Endothelin	↑[119]	↑[119–121]	↑[122–124]		↑[125–127]	↑[126–133]	↑[134]<br>↓[135]		↑[125,127–129,133,134,136–156]<br>—[157]	↑[127,128,134,136,137,143,156,158]<br>~[159]	↑[127,128,136,137,140,143]
glutahion, oxidized											
Guanylin											
Hyaluronic acid					↑[160,161]	↑[176–182]	↓[166]	↑[167]	↑[140,160–163,166–170]<br>↓[165,171,172]<br>~[163,173]	↓[160–164,166,168,170–172,174–177]	↑[140,161,162,165–167,178,179]
IL-8	↑[180]		↑[181]<br>—[182]			↓[183]			↑[183–191]<br>—[183]	↑[185,186,188–194]<br>—[183]	
Insulin-like growth factor 1					↑[195,196]<br>↓[197,198]<br>~[199]	↓[197]	↓[198,200–205]		↑[196,204,206–219]<br>~[199]	↑[195,199,209,211,213,215,217,220–223]<br>~[199]	↑[212,214,223,224]<br>↓[197,198,215,225–227]
Interleukin-10						↓[228–231]	↑[232]<br>↓[228]		↓[231,233–239]	↓[231]	↓[228,229,240]<br>—[241,242]
Interleukin-18	↑[243,244]		↑[245–247]	↑[243,244]	↑[248]	↑[244,248–253]			↑[250,254–257]	↑[248,250,258,259]	↑[248–250,252–255,258,260–265]
Interleukin-1β	↑[266–268]<br>~[269]	↑[267,270,271]	—<br>[267,268,272]	↑[266,268]<br>~[273]	—[274]	↑[274–282]	↑[283,284]	↑[268]	↑[285–293]	↑[287,292,294,295]	↑[267,278,279,282,283,287,290,295–297]
Interleukin-6	↑[298–303]	↑[299,300,304]	↑[11,272,305–310]	↑[299,301,303,311]	↑[312]	↑↓[311]	↑[313,314]	↑[302]	↑[315–318]	↑[317–323]	↑[299,301]
Methionine-Enkephalin											
Motiline											
Myoglobin											

Neuropeptide Y							↑[324]		↑[324–333]<br>~[334]	↑[330,332,335]	↑[324,330,336,337]<br>~[334]
Osteocalcin											
Parathyroid hormone	↑[338,339]	↑[338,340,341]<br>↓[342,343]	↑[310,344]<br>—[345]	↑[338]	↓[342]				—[346]		
Prolactin									↑[347,348]		
Resistin					↑[349,350]	↑[351,352]	↑[353]		↑[354–360]	↑[354,355,357,<br>359,361,362]	↑[350,352,352,355,<br>358,359,361]
Retinol Binding Protein											
Soluble intercellular adhesion molecule										↑[363]	
Substance P									↑[364,365]		
Tumor Necrosis Factor Alpha	↑[273,366–<br>372]	↑[369]	↑[307,310]<br>—[14,309]	↑[273,366,369,3<br>73]	↑[374–383]	↑[374–<br>376,378,380–<br>390]<br>~[391]	↑[384,392–<br>402]		↑[374,377–<br>379,384,389,39<br>3,398,400,403–<br>413]	↑[373,374,377<br>–<br>379,384,386,38<br>9,403,404,407–<br>411,414–418]	↑[382,383,387,390,<br>392,397,399,403,41<br>6]
Uroguanylin											
Vascular endothelial growth factor					↑[319]	↑[319]<br>—[419]	—[420]<br>↓[421]		↑[422–430]<br>—[319,419]<br>↓[431]	↑[432–<br>434,319,435,42<br>6,428,436]	↑[319,437]
Vasoactive intestinal peptide						↓[438]			↓[438]	↓[438]	↓[438]
Vasopressin											
α1-Acid glycoprotein											
β-2-Microglobulin											
β-Endorphin											
κ-Ig light chain											
λ-Ig Light Chain											

Table S2. Full table of the PBURM and their influence on calcification, as well as their effects on VC related processes in VSMC; ↑ = increase, ↓ = decrease, — = tested, but no effect, ~ = unclear effect.

Uremic retention molecule	Calcification - in vitro	Calcification - in vivo	Calcification - Clinical studies	Osteogenesis	Oxidative stress	Inflammation	Apoptosis	Senescence	Proliferation	Migration	Atherosclerosis
2-methoxyresorcinol											
3-Carboxy-4-Methyl-5-Propyl-2-Furanpropanoic Acid (CMPF)											
3-Deoxyglucosone					↑[439]				↑[439]		
Acrolein					[440]	↑[441,442]	↑[442,443]	↑[442]	~[444]		↑[445]
Angiogenin											
Dihydroxyphenylalanine (PB-DOPA)											
Fructoselysine											
Glyoxal											
Hippuric acid											
Homocysteine	↑[446–450]	↑[446,449–452]	↑[309,451,453–458] —[272,459–462]	↑[446,447,449,450]	↑[463–471]	↑[464,466,469,471–475]	↑[467,476] —[477]		↑[466,467,469,475–488] ~[489]	↑[481,484,490,491]	↑[446,469,471,475,477,484,486,488,492,493]
Hydroquinone											
Indican											
Indole-3-acetic acid											
Indoxyl sulfate	↑[180,494–497]	↑[494,497,498]	↑[499,500] —[501]	↑[180,496,497,502]	↑[502–507]	↑[505,508]	↑[495]	↑[495,495]	↑[504–506,509–512] ↓[513] ~[495]	↑[495,503,504]	↑[495,503,504,514]
Indoxyl-β-D-glucoronide											
Kinurenine											
Kynurenic Acid											

Leptin	↑[515–517]	↑[515,518,519]	↑[10,520–522]<br>—[11,14]	↑[515,515,517,518,523]	↑[524,525]	↑[524,526–528]	↑[525]<br>↓[529]		↑[516,524–528,530–541]<br>↓[542–544]	↑[516,525,528,533,540,541]<br>↓[542]	↑[516,528,530–532,538–540,545–547]<br>~[525,548]
Melatonin					↓[549–551]	↓[549,550,552,553]	↓[550]		↓[553]	↓[549]	↓[549]
Methylglyoxal					↑[554–560]	↑[555,556,559,561]			↑[562,563]		
N(6)-Carboxymethyllysine	↑[564]	↑[564]	↑[565]	↑[564]		↑[566]	↓[567]		↑[567]		↑[564]
p-cresol						↑[568,569]			↑[568]	↑[568]	
p-Cresylsulfate (t		↑[498]	↑[570]	↑[571]	↑[571,572]	↑[498,571]			↑[573]	↑[573]	↑[569,571,573]
p-hydroxy-hippuric acid											
Pentosidine											
Phenol											
Phenylacetic acid					↑[574]						
Putrescine							—[575]		↑[576,577]		↑[577,578]
Quinolinic Acid											
Spermidine					↓[579]	↓[579]	↑[575]		↑[580,581]	↑[582]	↓[579,583]
Spermine	↓[584]				↑[585]		↑[575,586]		↑[576,581,587]<br>↓[588]		
Thiocyanate											↑[589]

Table S3. Full table of LMWS and their influence on calcification, as well as their effects on VC related processes in VSMC,↑ = increase, ↓ = decrease, — = tested, but no effect, ~ = unclear effect.

Uremic retention molecule.	Calcification - in vitro	Calcification - in vivo	Calcification - Clinical studies	Osteogenesis	Oxidative stress	Inflammation	Apoptosis	Senescence	Proliferation	Migration	Atherosclerosis
1-Methyladenosine											
1-Methylguanosine											
1-Methylinosine											
2-Heptenal											
2-Hexenal											
2-Nonenal											
2-Octenal											
4-Decenal											
4-HO-Decenal											
4-HO-Hexenal					↑[590]	~[590]	↑[590]				
4-HO-Nonenal					↑[591–593]	↑[591,593,594] —[595]	↑[593,594,596, 597]		↑[593–595,598–603] ~[604]	↑[595,598] ~[604]	↑[593,594,605]
4-HO-Octenal											
4-Pyridone-3-carboxamide-1-β-D-ribonucleoside											
8-Hydroxy-2′-deoxyguanosine					↓[606]	↓[606]			↓[606]	↓[606]	↓[606]
anthranilic acid											
Arabinitol											
Argininic Acid											
Asymetric Dimethylarginine	↓[607]		↑[608–610,609,611,612] —[613]		↑[614]	↑[615]	↑[614]		↑[615,616] —[607]	↓[617,618]	
Benzylalcohol											
Creatine											

Creatinine											
Cystein					↑[619]				—[619]		
Cytidine											
Decanal											
Dimethylamine											
Dimethylglycine											
Erythritol											
Ethylamine											
Guanidine	—[607]								—[607]		
Guanidino acetic acid	—[607]								—[607]		
Guanidino succinic acid	↓[607]								—[607]		
Heptanal											
Hexanal											
Hypoxanthine					↑[620,621]	↑[622]			↓[623]<br>—[624]		
Inosine											
Malondialdehyde (MDA)							↑[596]				
Mannitol						↑[625,626]<br>—[627]	↑[628–630]<br>—[631]		—[631,632]	—[631]	
Methylguanidine	—[607]								—[607]		
Monomethylamine	↓[633]			↓[633]			↑[634]				
Myoinositol											
N-methyl-2-pyridone-5-carboxamide											
N-methyl-4-pyridone-3-carboxamide											
N2,N2-Dimethylguanosine											
N4-Acetylcytidine											
N6-Methyladenosine											

N6-Threonylcarbamoyladenosine											
Neopterin						↑[635–637]<br>↓[638]	↑[636]<br>—[638]		↓[638]	↓[638]	↓[638]
Nicotinamide									↓[639]		
Noadrenalin	↑[640]	↑[640]		↑[640]					↑[329,641–653]	↑[648,654]	
Nonanal											
Orotic Acid											
Orotidine											
Oxalate											
Phenylacetylglutamine											
Pseudouridine											
S-Adenosylhomocysteine											
Sorbitol									—[655]		
Symmetric Dimethylarginine	↓[607]								—[607]		
Taurocyamine											
Threitol											
Thymine											
Trimethylamine											
Trimethylamine-N-oxide	↑[656]	↑[656]	—[657]	↑[656]		↑[656,658]					↑[658]
Urea							↑[659]				↑[660]
Uric Acid	↑[661,662]	↑[662]	↑[663–666]<br>—[667]	↑[661,662]	↑[668–671]	↑[661,669,670,672–675]			↑[624,668,672,673,676–678]<br>↓[623,623]	↑[673]	↑[669]
Uridine									↑[679]		
Xanthine									↓[623]<br>—[624,677]		
Xanthosine											

α-keto-δ-Guanidinovaleric Acid		
α-N-Acetylarginine		
β-Guanidinopropionic Acid	—[607]	—[607]
β-Lipotropin		
γ-guanidinobutyric Acid	—[607]	—[607]

References.

1. Zhan: J.-K.; Wang, Y.-J.; Wang, Y.; Tang, Z.-Y.; Tan, P.; Huang, W.; Liu, Y.-S. Adiponectin attenuates the osteoblastic differentiation of vascular smooth muscle cells through the AMPK/mTOR pathway. *Exp. Cell Res.* **2014**, *323*, 352–358.
2. Zhan, J.-K.; Wang, Y.-J.; Wang, Y.; Wang, S.; Tan, P.; Huang, W.; Liu, Y.-S. The mammalian target of rapamycin signalling pathway is involved in osteoblastic differentiation of vascular smooth muscle cells. *Can. J. Cardiol.* **2014**, *30*, 568–575.
3. Son, B.-K.; Akishita, M.; Iijima, K.; Kozaki, K.; Maemura, K.; Eto, M.; Ouchi, Y. Adiponectin antagonizes stimulatory effect of tumor necrosis factor-alpha on vascular smooth muscle cell calcification: Regulation of growth arrest-specific gene 6-mediated survival pathway by adenosine 5′-monophosphate-activated protein kinase. *Endocrinology* **2008**, *149*, 1646–1653.
4. Niknam, S.; Ghatreh-Samani, K.; Farrokhi, E. The effect of adiponectin on osteonectin gene expression by oxidized low density lipoprotein-treated vascular smooth muscle cells. *Int. J. Mol. Cell. Med.* **2015**, *4*, 60–66.
5. Lu, Y.; Bian, Y.; Wang, Y.; Bai, R.; Wang, J.; Xiao, C. Globular adiponectin reduces vascular calcification via inhibition of ER-stress-mediated smooth muscle cell apoptosis. *Int. J. Clin. Exp. Pathol.* **2015**, *8*, 2545–2554.
6. Luo, X.-H.; Zhao, L.-L.; Yuan, L.-Q.; Wang, M.; Xie, H.; Liao, E.-Y. Development of arterial calcification in adiponectin-deficient mice: Adiponectin regulates arterial calcification. *J. Bone Miner. Res. Off. J. Am. Soc. Bone Miner. Res.* **2009**, *24*, 1461–1468.
7. Maahs, D.M.; Ogden, L.G.; Kinney, G.L.; Wadwa, P.; Snell-Bergeon, J.K.; Dabelea, D.; Hokanson, J.E.; Ehrlich, J.; Eckel, R.H.; Rewers, M. Low plasma adiponectin levels predict progression of coronary artery calcification. *Circulation* **2005**, *111*, 747–753.
8. Steffes, M.W.; Gross, M.D.; Lee, D.-H.; Schreiner, P.J.; Jacobs, D.R. Adiponectin, visceral fat, oxidative stress, and early macrovascular disease: The Coronary Artery Risk Development in Young Adults Study. *Obes. Silver Spring Md* **2006**, *14*, 319–326.
9. Tanna, N.; Patel, K.; Moore, A.E.; Dulnoan, D.; Edwards, S.; Hampson, G. The relationship between circulating adiponectin, leptin and vaspin with bone mineral density (BMD), arterial calcification and stiffness: A cross-sectional study in post-menopausal women. *J. Endocrinol. Invest.* **2017**, *40*, 1345–1353.
10. Aoqui, C.; Cuppari, L.; Kamimura, M.A.; Canziani, M.E.F. Increased visceral adiposity is associated with coronary artery calcification in male patients with chronic kidney disease. *Eur. J. Clin. Nutr.* **2013**, *67*, 610–614.
11. Larsen, B.A.; Laughlin, G.A.; Cummins, K.; Barrett-Connor, E.; Wassel, C.L. Adipokines and severity and progression of coronary artery calcium: Findings from the Rancho Bernardo Study. *Atherosclerosis* **2017**, *265*, 1–6.
12. Värri, M.; Niskanen, L.; Tuomainen, T.; Honkanen, R.; Kröger, H.; Tuppurainen, M.T. Association of adipokines and estradiol with bone and carotid calcifications in postmenopausal women. *Climacteric, J. Int. Menopause Soc.* **2016**, *19*, 204–211.
13. Hyun, Y.Y.; Kim, H.; Oh, Y.K.; Oh, K.-H.; Ahn, C.; Sung, S.A.; Choi, K.H.; Kim, S.W.; Lee, K.-B. High fibroblast growth factor 23 is associated with coronary calcification in patients with high adiponectin: Analysis from the KoreaN cohort study for Outcome in patients With Chronic Kidney Disease (KNOW-CKD) study. *Nephrol. Dial. Transplant. Off. Publ. Eur. Dial. Transpl. Assoc.—Eur. Ren. Assoc.* **2019**, *34*, 123–129.
14. Mehta, A.; Patel, J.; Al Rifai, M.; Ayers, C.R.; Neeland, I.J.; Kanaya, A.M.; Kandula, N.; Blaha, M.J.; Nasir, K.; Blumenthal, R.S.; et al. Inflammation and coronary artery calcification in South Asians: The Mediators of Atherosclerosis in South Asians Living in America (MASALA) study. *Atherosclerosis* **2018**, *270*, 49–56.
15. Ishihara, T.; Haraguchi, G.; Konishi, M.; Ohigashi, H.; Saito, K.; Nakano, Y.; Isobe, M. Effect of adiponectin on cardiac allograft vasculopathy. *Circ. J. Off. J. Jpn. Circ. Soc.* **2011**, *75*, 2005–2012.
16. Zhang, W.; Shu, C.; Li, Q.; Li, M.; Li, X. Adiponectin affects vascular smooth muscle cell proliferation and apoptosis through modulation of the mitofusin-2-mediated Ras-Raf-Erk1/2 signaling pathway. *Mol. Med. Rep.* **2015**, *12*, 4703–4707.
17. Luo, L.; Zheng, W.; Lian, G.; Chen, H.; Li, L.; Xu, C.; Xie, L. Combination treatment of adipose-derived stem cells and adiponectin attenuates pulmonary arterial hypertension in rats by inhibiting pulmonary arterial smooth muscle cell proliferation and regulating the AMPK/BMP/Smad pathway. *Int. J. Mol. Med.* **2018**, *41*, 51–60.

18. Fairaq, A.; Shawky, N.M.; Osman, I.; Pichavaram, P.; Segar, L. AdipoRon, an adiponectin receptor agonist, attenuates PDGF-induced VSMC proliferation through inhibition of mTOR signaling independent of AMPK: Implications toward suppression of neointimal hyperplasia. *Pharmacol. Res.* **2017**, *119*, 289–302.
19. Lamers, D.; Schlich, R.; Greulich, S.; Sasson, S.; Sell, H.; Eckel, J. Oleic acid and adipokines synergize in inducing proliferation and inflammatory signalling in human vascular smooth muscle cells. *J. Cell. Mol. Med.* **2011**, *15*, 1177–1188.
20. Arita, Y.; Kihara, S.; Ouchi, N.; Maeda, K.; Kuriyama, H.; Okamoto, Y.; Kumada, M.; Hotta, K.; Nishida, M.; Takahashi, M.; et al. Adipocyte-derived plasma protein adiponectin acts as a platelet-derived growth factor-BB-binding protein and regulates growth factor-induced common postreceptor signal in vascular smooth muscle cell. *Circulation* **2002**, *105*, 2893–2898.
21. Matsuda, M.; Shimomura, I.; Sata, M.; Arita, Y.; Nishida, M.; Maeda, N.; Kumada, M.; Okamoto, Y.; Nagaretani, H.; Nishizawa, H.; et al. Role of adiponectin in preventing vascular stenosis. The missing link of adipo-vascular axis. *J. Biol. Chem.* **2002**, *277*, 37487–37491.
22. Wang, Y.; Lam, K.S.L.; Xu, J.Y.; Lu, G.; Xu, L.Y.; Cooper, G.J.S.; Xu, A. Adiponectin inhibits cell proliferation by interacting with several growth factors in an oligomerization-dependent manner. *J. Biol. Chem.* **2005**, *280*, 18341–18347.
23. Weng, M.; Raher, M.J.; Leyton, P.; Combs, T.P.; Scherer, P.E.; Bloch, K.D.; Medoff, B.D. Adiponectin decreases pulmonary arterial remodeling in murine models of pulmonary hypertension. *Am. J. Respir. Cell Mol. Biol.* **2011**, *45*, 340–347.
24. Fuerst, M.; Taylor, C.G.; Wright, B.; Tworek, L.; Zahradka, P. Inhibition of smooth muscle cell proliferation by adiponectin requires proteolytic conversion to its globular form. *J. Endocrinol.* **2012**, *215*, 107–117.
25. Motobayashi, Y.; Izawa-Ishizawa, Y.; Ishizawa, K.; Orino, S.; Yamaguchi, K.; Kawazoe, K.; Hamano, S.; Tsuchiya, K.; Tomita, S.; Tamaki, T. Adiponectin inhibits insulin-like growth factor-1-induced cell migration by the suppression of extracellular signal-regulated kinase 1/2 activation, but not Akt in vascular smooth muscle cells. *Hypertens. Res. Off. J. Jpn. Soc. Hypertens.* **2009**, *32*, 188–193.
26. Saneipour, M.; Ghatreh-Samani, K.; Heydarian, E.; Farrokhi, E.; Abdian, N. Adiponectin inhibits oxidized low density lipoprotein-induced increase in matrix metalloproteinase 9 expression in vascular smooth muscle cells. *ARYA Atheroscler.* **2015**, *11*, 191–195.
27. Okamoto, Y.; Kihara, S.; Ouchi, N.; Nishida, M.; Arita, Y.; Kumada, M.; Ohashi, K.; Sakai, N.; Shimomura, I.; Kobayashi, H.; et al. Adiponectin reduces atherosclerosis in apolipoprotein E-deficient mice. *Circulation* **2002**, *106*, 2767–2770.
28. Cai, Y.; Teng, X.; Pan, C.; Duan, X.; Tang, C.; Qi, Y. Adrenomedullin up-regulates osteopontin and attenuates vascular calcification via the cAMP/PKA signaling pathway. *Acta Pharmacol. Sin.* **2010**, *31*, 1359–1366.
29. Huang, Z.; Li, J.; Jiang, Z.; Qi, Y.; Tang, C.; Du, J. Effects of adrenomedullin, C-type natriuretic peptide, and parathyroid hormone-related peptide on calcification in cultured rat vascular smooth muscle cells. *J. Cardiovasc. Pharmacol.* **2003**, *42*, 89–97.
30. Zhou, Y.-B.; Gao, Q.; Li, P.; Han, Y.; Zhang, F.; Qi, Y.-F.; Tang, C.-S.; Gao, X.-Y.; Zhu, G.-Q. Adrenomedullin attenuates vascular calcification in fructose-induced insulin resistance rats. *Acta Physiol. Oxf. Engl.* **2013**, *207*, 437–446.
31. Liu, J.; Shimosawa, T.; Matsui, H.; Meng, F.; Supowit, S.C.; DiPette, D.J.; Ando, K.; Fujita, T. Adrenomedullin inhibits angiotensin II-induced oxidative stress via Csk-mediated inhibition of Src activity. *Am. J. Physiol. Heart Circ. Physiol.* **2007**, *292*, H1714–H1721.
32. Yoshimoto, T.; Fukai, N.; Sato, R.; Sugiyama, T.; Ozawa, N.; Shichiri, M.; Hirata, Y. Antioxidant effect of adrenomedullin on angiotensin II-induced reactive oxygen species generation in vascular smooth muscle cells. *Endocrinology* **2004**, *145*, 3331–3337.
33. Shichiri, M.; Fukai, N.; Ozawa, N.; Iwasaki, H.; Hirata, Y. Adrenomedullin is an autocrine/paracrine growth factor for rat vascular smooth muscle cells. *Regul. Pept.* **2003**, *112*, 167–173.
34. Iwasaki, H.; Eguchi, S.; Shichiri, M.; Marumo, F.; Hirata, Y. Adrenomedullin as a novel growth-promoting factor for cultured vascular smooth muscle cells: Role of tyrosine kinase-mediated mitogen-activated protein kinase activation. *Endocrinology* **1998**, *139*, 3432–3441.

35. Kano, H.; Kohno, M.; Yasunari, K.; Yokokawa, K.; Horio, T.; Ikeda, M.; Minami, M.; Hanehira, T.; Takeda, T.; Yoshikawa, J. Adrenomedullin as a novel antiproliferative factor of vascular smooth muscle cells. *J. Hypertens.* **1996**, *14*, 209–213.
36. Rossi, F.; Bertone, C.; Petricca, S.; Santiemma, V. Adrenomedullin antagonizes angiotensin II-stimulated proliferation of human aortic smooth muscle cells. *Peptides* **2006**, *27*, 2935–2941.
37. Ma, J.; Feng, Y.; Li, Z.; Tang, C. The effect of adrenomedullin and proadrenomedullin N-terminal 20 peptide on angiotensin II induced vascular smooth muscle cell proliferation. *Iran. J. Basic Med. Sci.* **2016**, *19*, 49–54.
38. Li, W.; Kong, Q.Y.; Zhao, C.F.; Zhao, F.; Li, F.H.; Xia, W.; Wang, R.; Hu, Y.M.; Hua, M. Adrenomedullin and adrenotensin regulate collagen synthesis and proliferation in pulmonary arterial smooth muscle cells. *Braz. J. Med. Biol. Res. Rev. Bras. Pesqui. Medicas E Biol.* **2013**, *46*, 1047–1055.
39. Wang, X.; Xue, L.; Tong, L. [Influence of vasoactive peptides on homocysteine-induced proliferation of cultured rabbit vascular smooth muscle cell]. *Zhonghua Yi Xue Za Zhi* **1999**, *79*, 411–413.
40. Li, C.; Dai, A.; Huang, C. [Effects of adrenomedullin regulating inducible nitric oxide synthase on proliferation and apoptosis in hypoxic pulmonary artery smooth muscle cells]. *Zhongguo Ying Yong Sheng Li Xue Za Zhi Zhongguo Yingyong Shenglixue Zazhi Chin. J. Appl. Physiol.* **2005**, *21*, 187–191.
41. Jiang, W.; Yang, J.-H.; Pan, C.-S.; Qi, Y.-F.; Pang, Y.-Z.; Tang, C.-S. Effects of adrenomedullin on cell proliferation in rat adventitia induced by aldosterone. *J. Hypertens.* **2004**, *22*, 1953–1961.
42. Kohno, M.; Yokokawa, K.; Kano, H.; Yasunari, K.; Minami, M.; Hanehira, T.; Yoshikawa, J. Adrenomedullin is a potent inhibitor of angiotensin II-induced migration of human coronary artery smooth muscle cells. *Hypertens. Dallas Tex 1979* **1997**, *29*, 1309–1313.
43. Horio, T.; Kohno, M.; Kano, H.; Ikeda, M.; Yasunari, K.; Yokokawa, K.; Minami, M.; Takeda, T. Adrenomedullin as a novel antimigration factor of vascular smooth muscle cells. *Circ. Res.* **1995**, *77*, 660–664.
44. Fukai, N.; Shichiri, M.; Ozawa, N.; Matsushita, M.; Hirata, Y. Coexpression of calcitonin receptor-like receptor and receptor activity-modifying protein 2 or 3 mediates the antimigratory effect of adrenomedullin. *Endocrinology* **2003**, *144*, 447–453.
45. Gáll, T.; Pethő, D.; Nagy, A.; Hendrik, Z.; Méhes, G.; Potor, L.; Gram, M.; Åkerström, B.; Smith, A.; Nagy, P.; et al. Heme Induces Endoplasmic Reticulum Stress (HIER Stress) in Human Aortic Smooth Muscle Cells. *Front. Physiol.* **2018**, *9*, 1595.
46. De Vito, P.; Di Nardo, P.; Palmery, M.; Peluso, I.; Luly, P.; Baldini, P.M. Oxidant-induced pHi/Ca2+ changes in rat aortic smooth muscle cells. The role of atrial natriuretic peptide. *Mol. Cell. Biochem.* **2003**, *252*, 353–362.
47. Madiraju, P.; Hossain, E.; Anand-Srivastava, M.B. Natriuretic peptide receptor-C activation attenuates angiotensin II-induced enhanced oxidative stress and hyperproliferation of aortic vascular smooth muscle cells. *Mol. Cell. Biochem.* **2018**, *448*, 77–89.
48. Deprez, I.; Darmon, M.E.; Hira, M.; Adam, M.; Sanquer, S.; Teiger, E.; Chetboul, V.; Eloit, M.; Adnot, S.; Pham, I. Adenovirus-mediated transfer of the atrial natriuretic peptide gene in rat pulmonary vascular smooth muscle cells leads to apoptosis. *J. Lab. Clin. Med.* **2001**, *137*, 155–164.
49. Mtairag, E.M.; Houard, X.; Rais, S.; Pasquier, C.; Oudghiri, M.; Jacob, M.-P.; Meilhac, O.; Michel, J.-B. Pharmacological potentiation of natriuretic peptide limits polymorphonuclear neutrophil-vascular cell interactions. *Arterioscler. Thromb. Vasc. Biol.* **2002**, *22*, 1824–1831.
50. Larifla, L.; Déprez, I.; Pham, I.; Rideau, D.; Louzier, V.; Adam, M.; Eloit, M.; Foucan, L.; Adnot, S.; Teiger, E. Inhibition of vascular smooth muscle cell proliferation and migration in vitro and neointimal hyperplasia in vivo by adenoviral-mediated atrial natriuretic peptide delivery. *J. Gene Med.* **2012**, *14*, 459–467.
51. Arjona, A.A.; Hsu, C.A.; Wrenn, D.S.; Hill, N.S. Effects of natriuretic peptides on vascular smooth-muscle cells derived from different vascular beds. *Gen. Pharmacol.* **1997**, *28*, 387–392.
52. Itoh, H.; Pratt, R.E.; Dzau, V.J. Atrial natriuretic polypeptide inhibits hypertrophy of vascular smooth muscle cells. *J. Clin. Invest.* **1990**, *86*, 1690–1697.
53. Rybalkin, S.D.; Yan, C.; Bornfeldt, K.E.; Beavo, J.A. Cyclic GMP phosphodiesterases and regulation of smooth muscle function. *Circ. Res.* **2003**, *93*, 280–291.
54. Hashim, S.; Li, Y.; Anand-Srivastava, M.B. Small cytoplasmic domain peptides of natriuretic peptide receptor-C attenuate cell proliferation through Gialpha protein/MAP kinase/PI3-kinase/AKT pathways. *Am. J. Physiol. Heart Circ. Physiol.* **2006**, *291*, H3144–H3153.

55. Dhaunsi, G.S.; Hassid, A. Atrial and C-type natriuretic peptides amplify growth factor activity in primary aortic smooth muscle cells. *Cardiovasc. Res.* **1996**, *31*, 37–47.
56. Awazu, M. Inhibition of platelet-derived growth factor receptor tyrosine kinase by atrial natriuretic peptide. *Kidney Int.* **1997**, *52*, 356–362.
57. Neuser, D.; Stasch, J.P.; Knorr, A.; Kazda, S. Inhibition by atrial natriuretic peptide of endothelin-1-stimulated proliferation of vascular smooth-muscle cells. *J. Cardiovasc. Pharmacol.* **1993**, *22 Suppl 8*, S257-261.
58. Itoh, H.; Nakao, K. Antagonism between the vascular renin-angiotensin and natriuretic peptide systems in vascular remodelling. *Blood Pressure. Supplement* **1994**, *5*, 49–53.
59. Hamad, A.M.; Johnson, S.R.; Knox, A.J. Antiproliferative effects of NO and ANP in cultured human airway smooth muscle. *Am. J. Physiol.* **1999**, *277*, L910–L918.
60. Wu, J.M.; Cheng, T.; Sun, S.D.; Niu, D.D.; Zhang, J.X.; Wang, S.H.; Tang, J.; Tang, C.S. Effect of endothelin, angiotensin II and ANP on proliferation of vascular smooth muscle cells and cardiomyocytes. *Sci. China B* **1993**, *36*, 948–953.
61. Wu, J.M.; Sun, S.D.; Liao, Y.B.; Tang, C.S. [Effect of ANP on angiotensin II--stimulated multiplication and c-fos oncogene expression of SHR vascular smooth muscle cells]. *Sheng Li Xue Bao* **1992**, *44*, 181–185.
62. Hirata, K.; Akita, H.; Yokoyama, M.; Watanabe, Y. Impaired vasodilatory response to atrial natriuretic peptide during atherosclerosis progression. *Arterioscler. Thromb. J. Vasc. Biol.* **1992**, *12*, 99–105.
63. Kariya, K.; Kawahara, Y.; Araki, S.; Fukuzaki, H.; Takai, Y. Antiproliferative action of cyclic GMP-elevating vasodilators in cultured rabbit aortic smooth muscle cells. *Atherosclerosis* **1989**, *80*, 143–147.
64. Dong, M.Q.; Zhu, M.Z.; Yu, J.; Shang, L.J.; Feng, H.S. [Comparison of inhibitory effects of three natriuretic peptides on the proliferation of pulmonary artery smooth muscle cells of rats]. *Sheng Li Xue Bao* **2000**, *52*, 252–254.
65. Ikeda, M.; Kohno, M.; Yasunari, K.; Yokokawa, K.; Horio, T.; Ueda, M.; Morisaki, N.; Yoshikawa, J. Natriuretic peptide family as a novel antimigration factor of vascular smooth muscle cells. *Arterioscler. Thromb. Vasc. Biol.* **1997**, *17*, 731–736.
66. Kohno, M.; Yokokawa, K.; Yasunari, K.; Kano, H.; Minami, M.; Ueda, M.; Yoshikawa, J. Effect of natriuretic peptide family on the oxidized LDL-induced migration of human coronary artery smooth muscle cells. *Circ. Res.* **1997**, *81*, 585–590.
67. Schröder, K.; Helmcke, I.; Palfi, K.; Krause, K.-H.; Busse, R.; Brandes, R.P. Nox1 mediates basic fibroblast growth factor-induced migration of vascular smooth muscle cells. *Arterioscler. Thromb. Vasc. Biol.* **2007**, *27*, 1736–1743.
68. Peluso, J.J. Basic fibroblast growth factor (bFGF) regulation of the plasma membrane calcium ATPase (PMCA) as part of an anti-apoptotic mechanism of action. *Biochem. Pharmacol.* **2003**, *66*, 1363–1369.
69. Ye, G.; Fu, Q.; Jiang, L.; Li, Z. Vascular smooth muscle cells activate PI3K/Akt pathway to attenuate myocardial ischemia/reperfusion-induced apoptosis and autophagy by secreting bFGF. *Biomed. Pharmacother. Biomedecine Pharmacother.* **2018**, *107*, 1779–1785.
70. Miyamoto, T.; Leconte, I.; Swain, J.L.; Fox, J.C. Autocrine FGF signaling is required for vascular smooth muscle cell survival in vitro. *J. Cell. Physiol.* **1998**, *177*, 58–67.
71. Fox, J.C.; Shanley, J.R. Antisense inhibition of basic fibroblast growth factor induces apoptosis in vascular smooth muscle cells. *J. Biol. Chem.* **1996**, *271*, 12578–12584.
72. Li, R.; Cindrova-Davies, T.; Skepper, J.N.; Sellers, L.A. Prostacyclin induces apoptosis of vascular smooth muscle cells by a cAMP-mediated inhibition of extracellular signal-regulated kinase activity and can counteract the mitogenic activity of endothelin-1 or basic fibroblast growth factor. *Circ. Res.* **2004**, *94*, 759–767.
73. Liang, M.; He, L. Inhibitory effects of ligustilide and butylidenephthalide on bFGF-stimulated proliferation of rat smooth muscle cells. *Yao Xue Xue Bao* **2006**, *41*, 161–165.
74. Inoue, Y.; King, T.E.; Barker, E.; Daniloff, E.; Newman, L.S. Basic fibroblast growth factor and its receptors in idiopathic pulmonary fibrosis and lymphangioleiomyomatosis. *Am. J. Respir. Crit. Care Med.* **2002**, *166*, 765–773.
75. Yu, D.; Rui, X.; He, S. Effect of heparin-derived oligosaccharide on bFGFR1 and bFGFR2 in vascular smooth muscle cells. *Vasc. Endovascular Surg.* **2014**, *48*, 289–296.
76. Millette, E.; Rauch, B.H.; Defawe, O.; Kenagy, R.D.; Daum, G.; Clowes, A.W. Platelet-derived growth factor-BB-induced human smooth muscle cell proliferation depends on basic FGF release and FGFR-1 activation. *Circ. Res.* **2005**, *96*, 172–179.

77. Wang, Z.; Rao, P.J.; Shillcutt, S.D.; Newman, W.H. Angiotensin II induces proliferation of human cerebral artery smooth muscle cells through a basic fibroblast growth factor (bFGF) dependent mechanism. *Neurosci. Lett.* **2005**, *373*, 38–41.
78. Rauch, B.H.; Millette, E.; Kenagy, R.D.; Daum, G.; Fischer, J.W.; Clowes, A.W. Syndecan-4 is required for thrombin-induced migration and proliferation in human vascular smooth muscle cells. *J. Biol. Chem.* **2005**, *280*, 17507–17511.
79. DeLong, S.A.; Moon, J.J.; West, J.L. Covalently immobilized gradients of bFGF on hydrogel scaffolds for directed cell migration. *Biomaterials* **2005**, *26*, 3227–3234.
80. Herbert, J.M.; Bono, F.; Savi, P. The mitogenic effect of H2O2 for vascular smooth muscle cells is mediated by an increase of the affinity of basic fibroblast growth factor for its receptor. *FEBS Lett.* **1996**, *395*, 43–47.
81. Hoshi, S.; Goto, M.; Koyama, N.; Nomoto, K.; Tanaka, H. Regulation of vascular smooth muscle cell proliferation by nuclear factor-kappaB and its inhibitor, I-kappaB. *J. Biol. Chem.* **2000**, *275*, 883–889.
82. Myler, H.A.; West, J.L. Heparanase and platelet factor-4 induce smooth muscle cell proliferation and migration via bFGF release from the ECM. *J. Biochem. (Tokyo)* **2002**, *131*, 913–922.
83. Huang, S.L.; Ding, B.; Li, J.; Yu, Q.S.; Guo, Z.G. Antisense Ca(2+)-calmodulin dependent protein kinase oligonucleotide inhibits bFGF-induced proliferation of rat vascular smooth muscle cells. *Acta Pharmacol. Sin.* **2000**, *21*, 229–233.
84. Segev, A.; Aviezer, D.; Safran, M.; Gross, Z.; Yayon, A. Inhibition of vascular smooth muscle cell proliferation by a novel fibroblast growth factor receptor antagonist. *Cardiovasc. Res.* **2002**, *53*, 232–241.
85. Zou, H.; Nie, X.; Zhang, Y.; Hu, M.; Zhang, Y.A. Effect of basic fibroblast growth factor on the proliferation, migration and phenotypic modulation of airway smooth muscle cells. *Chin. Med. J. (Engl.)* **2008**, *121*, 424–429.
86. Lindner, V.; Reidy, M.A. Expression of basic fibroblast growth factor and its receptor by smooth muscle cells and endothelium in injured rat arteries. An en face study. *Circ. Res.* **1993**, *73*, 589–595.
87. Hoshina, K.; Koyama, H.; Miyata, T.; Shigematsu, H.; Takato, T.; Dalman, R.L.; Nagawa, H. Aortic wall cell proliferation via basic fibroblast growth factor gene transfer limits progression of experimental abdominal aortic aneurysm. *J. Vasc. Surg.* **2004**, *40*, 512–518.
88. Kennedy, S.H.; Rouda, S.; Qin, H.; Aho, S.; Selber, J.; Tan, E.M. Basic FGF regulates interstitial collagenase gene expression in human smooth muscle cells. *J. Cell. Biochem.* **1997**, *65*, 32–41.
89. Olson, N.E.; Chao, S.; Lindner, V.; Reidy, M.A. Intimal smooth muscle cell proliferation after balloon catheter injury. The role of basic fibroblast growth factor. *Am. J. Pathol.* **1992**, *140*, 1017–1023.
90. Liu, W.; Parikh, A.A.; Stoeltzing, O.; Fan, F.; McCarty, M.F.; Wey, J.; Hicklin, D.J.; Ellis, L.M. Upregulation of neuropilin-1 by basic fibroblast growth factor enhances vascular smooth muscle cell migration in response to VEGF. *Cytokine* **2005**, *32*, 206–212.
91. Kenagy, R.D.; Hart, C.E.; Stetler-Stevenson, W.G.; Clowes, A.W. Primate smooth muscle cell migration from aortic explants is mediated by endogenous platelet-derived growth factor and basic fibroblast growth factor acting through matrix metalloproteinases 2 and 9. *Circulation* **1997**, *96*, 3555–3560.
92. Alfke, H.; Kleb, B.; Klose, K.J. Nitric oxide inhibits the basic fibroblast growth factor-stimulated migration of bovine vascular smooth muscle cells in vitro. *VASA Z. Gefasskrankheiten* **2000**, *29*, 99–102.
93. Kato, Y.; Yokoyama, U.; Fujita, T.; Umemura, M.; Kubota, T.; Ishikawa, Y. Epac1 deficiency inhibits basic fibroblast growth factor-mediated vascular smooth muscle cell migration. *J. Physiol. Sci. JPS* **2019**, *69*, 175–184.
94. Jackson, C.L.; Reidy, M.A. Basic fibroblast growth factor: Its role in the control of smooth muscle cell migration. *Am. J. Pathol.* **1993**, *143*, 1024–1031.
95. Irani, C.; Goncharova, E.A.; Hunter, D.S.; Walker, C.L.; Panettieri, R.A.; Krymskaya, V.P. Phosphatidylinositol 3-kinase but not tuberin is required for PDGF-induced cell migration. *Am. J. Physiol. Lung Cell. Mol. Physiol.* **2002**, *282*, L854–L862.
96. Wu, J.; Mao, Z.; Hong, Y.; Han, L.; Gao, C. Conjugation of basic fibroblast growth factor on a heparin gradient for regulating the migration of different types of cells. *Bioconjug. Chem.* **2013**, *24*, 1302–1313.
97. Kohno, M.; Yokokawa, K.; Yasunari, K.; Minami, M.; Kano, H.; Hanehira, T.; Yoshikawa, J. Induction by lysophosphatidylcholine, a major phospholipid component of atherogenic lipoproteins, of human coronary artery smooth muscle cell migration. *Circulation* **1998**, *98*, 353–359.

98. Huang, Y.-H.; Wu, M.-P.; Pan, S.-C.; Su, W.-C.; Chen, Y.-W.; Wu, L.-W. STAT1 activation by venous malformations mutant Tie2-R849W antagonizes VEGF-A-mediated angiogenic response partly via reduced bFGF production. *Angiogenesis* **2013**, *16*, 207–222.
99. Wu, J.; Mao, Z.; Han, L.; Zhao, Y.; Xi, J.; Gao, C. A density gradient of basic fibroblast growth factor guides directional migration of vascular smooth muscle cells. *Colloids Surf. B Biointerfaces* **2014**, *117*, 290–295.
100. Six, I.; Mouquet, F.; Corseaux, D.; Bordet, R.; Letourneau, T.; Vallet, B.; Dosquet, C.C.; Dupuis, B.; Jude, B.; Bertrand, M.E.; et al. Protective effects of basic fibroblast growth factor in early atherosclerosis. *Growth Factors Chur Switz.* **2004**, *22*, 157–167.
101. Schaeffer, C.; Thomassin, L.; Rochette, L.; Connat, J.L. Apoptosis induced in vascular smooth muscle cells by oxidative stress is partly prevented by pretreatment with CGRP. *Ann. N. Y. Acad. Sci.* **2003**, *1010*, 733–737.
102. Yang, L.; Sakurai, T.; Kamiyoshi, A.; Ichikawa-Shindo, Y.; Kawate, H.; Yoshizawa, T.; Koyama, T.; Iesato, Y.; Uetake, R.; Yamauchi, A.; et al. Endogenous CGRP protects against neointimal hyperplasia following wire-induced vascular injury. *J. Mol. Cell. Cardiol.* **2013**, *59*, 55–66.
103. Zeng, S.-Y.; Yang, L.; Hong, C.-L.; Lu, H.-Q.; Yan, Q.-J.; Chen, Y.; Qin, X.-P. Evidence That ADAM17 Mediates the Protective Action of CGRP against Angiotensin II-Induced Inflammation in Vascular Smooth Muscle Cells. *Mediators Inflamm.* **2018**, *2018*, 2109352.
104. Wang, W.; Sun, W.; Wang, X. Intramuscular gene transfer of CGRP inhibits neointimal hyperplasia after balloon injury in the rat abdominal aorta. *Am. J. Physiol. Heart Circ. Physiol.* **2004**, *287*, H1582–H1589.
105. Schaeffer, C.; Vandroux, D.; Thomassin, L.; Athias, P.; Rochette, L.; Connat, J.-L. Calcitonin gene-related peptide partly protects cultured smooth muscle cells from apoptosis induced by an oxidative stress via activation of ERK1/2 MAPK. *Biochim. Biophys. Acta* **2003**, *1643*, 65–73.
106. Connat, J.L.; Schnüriger, V.; Zanone, R.; Schaeffer, C.; Gaillard, M.; Faivre, B.; Rochette, L. The neuropeptide calcitonin gene-related peptide differently modulates proliferation and differentiation of smooth muscle cells in culture depending on the cell type. *Regul. Pept.* **2001**, *101*, 169–178.
107. Li, X.-W.; Hu, C.-P.; Wu, W.-H.; Zhang, W.-F.; Zou, X.-Z.; Li, Y.-J. Inhibitory effect of calcitonin gene-related peptide on hypoxia-induced rat pulmonary artery smooth muscle cells proliferation: Role of ERK1/2 and p27. *Eur. J. Pharmacol.* **2012**, *679*, 117–126.
108. Qin, X.-P.; Ye, F.; Hu, C.-P.; Liao, D.-F.; Deng, H.-W.; Li, Y.-J. Effect of calcitonin gene-related peptide on angiotensin II-induced proliferation of rat vascular smooth muscle cells. *Eur. J. Pharmacol.* **2004**, *488*, 45–49.
109. Li, Y.; Fiscus, R.R.; Wu, J.; Yang, L.; Wang, X. The antiproliferative effects of calcitonin gene-related peptide in different passages of cultured vascular smooth muscle cells. *Neuropeptides* **1997**, *31*, 503–509.
110. Fang, L.; Chen, M.-F.; Xiao, Z.-L.; Liu, Y.; Yu, G.-L.; Chen, X.-B.; Xie, X.-M. Calcitonin gene-related peptide released from endothelial progenitor cells inhibits the proliferation of rat vascular smooth muscle cells induced by angiotensin II. *Mol. Cell. Biochem.* **2011**, *355*, 99–108.
111. Shi, B.; Cui, C.; Long, X.; Chen, W.; Zhao, R.; Liu, Z.; Ao, Z. [Effects of CGRP and CGRP receptor modified mesenchymal stem cells on proliferation and phenotypic transformation of vascular smooth muscle cells]. *Zhonghua Yi Xue Za Zhi* **2013**, *93*, 2372–2376.
112. Shi, B.; Chen, P.; Long, X.; Zhao, R.; Deng, W. [Role of CGRP modified mesenchymal stem cells in restenosis in rat model with carotid angioplasty]. *Zhonghua Yi Xue Za Zhi* **2015**, *95*, 1619–1624.
113. Long, X.; Cui, C.; Chen, P.; Wang, S.; Wang, D.; Xu, G.; Yao, X.; Shi, B. [Impact and related mechanism of exogenous receptor activity modifying protein 1 on calcitonin gene-related peptide modified bone marrow mesenchymal stem cells on the migration of vascular smooth muscle cells in vitro]. *Zhonghua Xin Xue Guan Bing Za Zhi* **2015**, *43*, 537–541.
114. Saia, R.S.; Bertozi, G.; Mestriner, F.L.; Antunes-Rodrigues, J.; Queiróz Cunha, F.; Cárnio, E.C. Cardiovascular and inflammatory response to cholecystokinin during endotoxemic shock. *Shock Augusta Ga* **2013**, *39*, 104–113.
115. Schulte, S.; Sun, J.; Libby, P.; Macfarlane, L.; Sun, C.; Lopez-Ilasaca, M.; Shi, G.-P.; Sukhova, G.K. Cystatin C deficiency promotes inflammation in angiotensin II-induced abdominal aortic aneurisms in atherosclerotic mice. *Am. J. Pathol.* **2010**, *177*, 456–463.
116. Cheng, X.W.; Kuzuya, M.; Nakamura, K.; Di, Q.; Liu, Z.; Sasaki, T.; Kanda, S.; Jin, H.; Shi, G.-P.; Murohara, T.; et al. Localization of cysteine protease, cathepsin S, to the surface of vascular smooth muscle cells by association with integrin alphanubeta3. *Am. J. Pathol.* **2006**, *168*, 685–694.

117. Sukhova, G.K.; Wang, B.; Libby, P.; Pan, J.-H.; Zhang, Y.; Grubb, A.; Fang, K.; Chapman, H.A.; Shi, G.-P. Cystatin C deficiency increases elastic lamina degradation and aortic dilatation in apolipoprotein E-null mice. *Circ. Res.* **2005**, *96*, 368–375.
118. Shi, G.P.; Sukhova, G.K.; Grubb, A.; Ducharme, A.; Rhode, L.H.; Lee, R.T.; Ridker, P.M.; Libby, P.; Chapman, H.A. Cystatin C deficiency in human atherosclerosis and aortic aneurysms. *J. Clin. Invest.* **1999**, *104*, 1191–1197.
119. Wu, S.Y.; Zhang, B.H.; Pan, C.S.; Jiang, H.F.; Pang, Y.Z.; Tang, C.S.; Qi, Y.F. Endothelin-1 is a potent regulator in vivo in vascular calcification and in vitro in calcification of vascular smooth muscle cells. *Peptides* **2003**, *24*, 1149–1156.
120. Essalihi, R.; Ouellette, V.; Dao, H.H.; McKee, M.D.; Moreau, P. Phenotypic modulation of vascular smooth muscle cells during medial arterial calcification: A role for endothelin? *J. Cardiovasc. Pharmacol.* **2004**, *44 Suppl 1*, S147-150.
121. Larivière, R.; Gauthier-Bastien, A.; Ung, R.-V.; St-Hilaire, J.; Mac-Way, F.; Richard, D.E.; Agharazii, M. Endothelin type A receptor blockade reduces vascular calcification and inflammation in rats with chronic kidney disease. *J. Hypertens.* **2017**, *35*, 376–384.
122. Wang, F.; Li, T.; Cong, X.; Hou, Z.; Lu, B.; Zhou, Z.; Chen, X. The Value of Big Endothelin-1 in the Assessment of the Severity of Coronary Artery Calcification. *Clin. Appl. Thromb. Off. J. Int. Acad. Clin. Appl. Thromb.* **2018**, *24*, 1042–1049.
123. Qing, P.; Li, X.-L.; Zhang, Y.; Li, Y.-L.; Xu, R.-X.; Guo, Y.-L.; Li, S.; Wu, N.-Q.; Li, J.-J. Association of Big Endothelin-1 with Coronary Artery Calcification. *PLoS ONE* **2015**, *10*, e0142458.
124. Rebić, D.; Rašić, S.; Hamzić-Mehmedbašić, A.; Džemidžić, J.; Kurtalić, E. Valvular calcification and left ventricular modifying in peritoneal dialysis patients. *Ren. Fail.* **2015**, *37*, 1316–1322.
125. Wedgwood, S.; Dettman, R.W.; Black, S.M. ET-1 stimulates pulmonary arterial smooth muscle cell proliferation via induction of reactive oxygen species. *Am. J. Physiol. Lung Cell. Mol. Physiol.* **2001**, *281*, L1058–L1067.
126. Idris-Khodja, N.; Ouerd, S.; Trindade, M.; Gornitsky, J.; Rehman, A.; Barhoumi, T.; Offermanns, S.; Gonzalez, F.J.; Neves, M.F.; Paradis, P.; et al. Vascular smooth muscle cell peroxisome proliferator-activated receptor γ protects against endothelin-1-induced oxidative stress and inflammation. *J. Hypertens.* **2017**, *35*, 1390–1401.
127. Trindade, M.; Oigman, W.; Fritsch Neves, M. Potential Role of Endothelin in Early Vascular Aging. *Curr. Hypertens. Rev.* **2017**, *13*, 33–40.
128. Ivey, M.E.; Osman, N.; Little, P.J. Endothelin-1 signalling in vascular smooth muscle: Pathways controlling cellular functions associated with atherosclerosis. *Atherosclerosis* **2008**, *199*, 237–247.
129. He, J.; Yi, B.; Chen, Y.; Huang, Q.; Wang, H.; Lu, K.; Fu, W. The ET-1-mediated carbonylation and degradation of ANXA1 induce inflammatory phenotype and proliferation of pulmonary artery smooth muscle cells in HPS. *PLoS ONE* **2017**, *12*, e0175443.
130. Yeager, M.E.; Belchenko, D.D.; Nguyen, C.M.; Colvin, K.L.; Ivy, D.D.; Stenmark, K.R. Endothelin-1, the unfolded protein response, and persistent inflammation: Role of pulmonary artery smooth muscle cells. *Am. J. Respir. Cell Mol. Biol.* **2012**, *46*, 14–22.
131. Lin, C.-C.; Lin, W.-N.; Hou, W.-C.; Hsiao, L.-D.; Yang, C.-M. Endothelin-1 induces VCAM-1 expression-mediated inflammation via receptor tyrosine kinases and Elk/p300 in human tracheal smooth muscle cells. *Am. J. Physiol. Lung Cell. Mol. Physiol.* **2015**, *309*, L211–L225.
132. Knobloch, J.; Feldmann, M.; Wahl, C.; Jungck, D.; Behr, J.; Stoelben, E.; Koch, A. Endothelin receptor antagonists attenuate the inflammatory response of human pulmonary vascular smooth muscle cells to bacterial endotoxin. *J. Pharmacol. Exp. Ther.* **2013**, *346*, 290–299.
133. Anggrahini, D.W.; Emoto, N.; Nakayama, K.; Widyantoro, B.; Adiarto, S.; Iwasa, N.; Nonaka, H.; Rikitake, Y.; Kisanuki, Y.Y.; Yanagisawa, M.; et al. Vascular endothelial cell-derived endothelin-1 mediates vascular inflammation and neointima formation following blood flow cessation. *Cardiovasc. Res.* **2009**, *82*, 143–151.
134. Kim, F.Y.; Barnes, E.A.; Ying, L.; Chen, C.; Lee, L.; Alvira, C.M.; Cornfield, D.N. Pulmonary artery smooth muscle cell endothelin-1 expression modulates the pulmonary vascular response to chronic hypoxia. *Am. J. Physiol. Lung Cell. Mol. Physiol.* **2015**, *308*, L368–L377.
135. Dai, F.; Mao, Z.; Xia, J.; Zhu, S.; Wu, Z. Fluoxetine protects against big endothelin-1 induced anti-apoptosis by rescuing Kv1.5 channels in human pulmonary arterial smooth muscle cells. *Yonsei Med. J.* **2012**, *53*, 842–848.
136. Little, P.J.; Ivey, M.E.; Osman, N. Endothelin-1 actions on vascular smooth muscle cell functions as a target for the prevention of atherosclerosis. *Curr. Vasc. Pharmacol.* **2008**, *6*, 195–203.

137. Sihvola, R.K.; Pulkkinen, V.P.; Koskinen, P.K.; Lemström, K.B. Crosstalk of endothelin-1 and platelet-derived growth factor in cardiac allograft arteriosclerosis. *J. Am. Coll. Cardiol.* **2002**, *39*, 710–717.
138. Lu, M.-H.; Chao, C.-F.; Tsai, S.-H.; Chen, J.-Y.; Chang, L.-T. Autocrine effects of endothelin on in vitro proliferation of vascular smooth muscle cells from spontaneously hypertensive and normotensive rats. *Clin. Exp. Hypertens. N. Y. N 1993* **2006**, *28*, 463–474.
139. Wort, S.J.; Woods, M.; Warner, T.D.; Evans, T.W.; Mitchell, J.A. Endogenously released endothelin-1 from human pulmonary artery smooth muscle promotes cellular proliferation: Relevance to pathogenesis of pulmonary hypertension and vascular remodeling. *Am. J. Respir. Cell Mol. Biol.* **2001**, *25*, 104–110.
140. Tanaka, Y.; Makiyama, Y.; Mitsui, Y. Endothelin-1 is involved in the growth promotion of vascular smooth muscle cells by hyaluronic acid. *Int. J. Cardiol.* **2000**, *76*, 39–47.
141. Ljuca, F.; Drevensek, G. Endothelin-1 induced vascular smooth muscle cell proliferation is mediated by cytochrome p-450 arachidonic acid metabolites. *Bosn. J. Basic Med. Sci.* **2010**, *10*, 223–226.
142. Chen, S.; Qiong, Y.; Gardner, D.G. A role for p38 mitogen-activated protein kinase and c-myc in endothelin-dependent rat aortic smooth muscle cell proliferation. *Hypertens. Dallas Tex 1979* **2006**, *47*, 252–258.
143. Lin, Y.-J.; Juan, C.-C.; Kwok, C.-F.; Hsu, Y.-P.; Shih, K.-C.; Chen, C.-C.; Ho, L.-T. Endothelin-1 exacerbates development of hypertension and atherosclerosis in modest insulin resistant syndrome. *Biochem. Biophys. Res. Commun.* **2015**, *460*, 497–503.
144. Sauter, G.; Wolf, S.; Risler, T.; Brehm, B. Influence of endothelin receptor antagonism on smooth muscle cell proliferation after chronic renal failure. *J. Cardiovasc. Pharmacol.* **2004**, *44 Suppl 1*, S165-167.
145. Janakidevi, K.; Fisher, M.A.; Del Vecchio, P.J.; Tiruppathi, C.; Figge, J.; Malik, A.B. Endothelin-1 stimulates DNA synthesis and proliferation of pulmonary artery smooth muscle cells. *Am. J. Physiol.* **1992**, *263*, C1295–C1301.
146. Bouallegue, A.; Vardatsikos, G.; Srivastava, A.K. Role of insulin-like growth factor 1 receptor and c-Src in endothelin-1- and angiotensin II-induced PKB phosphorylation, and hypertrophic and proliferative responses in vascular smooth muscle cells. *Can. J. Physiol. Pharmacol.* **2009**, *87*, 1009–1018.
147. Chen, S.; Law, C.S.; Gardner, D.G. Vitamin D-dependent suppression of endothelin-induced vascular smooth muscle cell proliferation through inhibition of CDK2 activity. *J. Steroid Biochem. Mol. Biol.* **2010**, *118*, 135–141.
148. Alberts, G.F.; Peifley, K.A.; Johns, A.; Kleha, J.F.; Winkles, J.A. Constitutive endothelin-1 overexpression promotes smooth muscle cell proliferation via an external autocrine loop. *J. Biol. Chem.* **1994**, *269*, 10112–10118.
149. Kida, T.; Chuma, H.; Murata, T.; Yamawaki, H.; Matsumoto, S.; Hori, M.; Ozaki, H. Chronic treatment with PDGF-BB and endothelin-1 synergistically induces vascular hyperplasia and loss of contractility in organ-cultured rat tail artery. *Atherosclerosis* **2011**, *214*, 288–294.
150. Lu, J.; Wang, X.; Xie, X.; Han, D.; Li, S.; Li, M. [Calcineurin/NFAT signaling pathway mediates endothelin-1-induced pulmonary artery smooth muscle cell proliferation by regulating phosphodiesterase-5]. *Nan Fang Yi Ke Da Xue Xue Bao* **2013**, *33*, 26–29.
151. Tan, Z.; Yang, D.; Lu, W. [Effect of inhibition by 17beta-estradiol on endothelin-induced proliferation of vascular smooth muscle cells]. *Zhongguo Ying Yong Sheng Li Xue Za Zhi Zhongguo Yingyong Shenglixue Zazhi Chin. J. Appl. Physiol.* **2001**, *17*, 251–254.
152. Maruyama, H.; Dewachter, C.; Belhaj, A.; Rondelet, B.; Sakai, S.; Remmelink, M.; Vachiery, J.-L.; Naeije, R.; Dewachter, L. Endothelin-Bone morphogenetic protein type 2 receptor interaction induces pulmonary artery smooth muscle cell hyperplasia in pulmonary arterial hypertension. *J. Heart Lung Transplant. Off. Publ. Int. Soc. Heart Transplant.* **2015**, *34*, 468–478.
153. Jiang, H.-N.; Zeng, B.; Chen, G.-L.; Lai, B.; Lu, S.-H.; Qu, J.-M. Lipopolysaccharide potentiates endothelin-1-induced proliferation of pulmonary arterial smooth muscle cells by upregulating TRPC channels. *Biomed. Pharmacother. Biomedecine Pharmacother.* **2016**, *82*, 20–27.
154. Jia, L.; Wang, R.; Tang, D.D. Abl regulates smooth muscle cell proliferation by modulating actin dynamics and ERK1/2 activation. *Am. J. Physiol. Cell Physiol.* **2012**, *302*, C1026–C1034.
155. Biasin, V.; Chwalek, K.; Wilhelm, J.; Best, J.; Marsh, L.M.; Ghanim, B.; Klepetko, W.; Fink, L.; Schermuly, R.T.; Weissmann, N.; et al. Endothelin-1 driven proliferation of pulmonary arterial smooth muscle cells is c-fos dependent. *Int. J. Biochem. Cell Biol.* **2014**, *54*, 137–148.
156. Rodríguez-Pascual, F.; Busnadiego, O.; Lagares, D.; Lamas, S. Role of endothelin in the cardiovascular system. *Pharmacol. Res.* **2011**, *63*, 463–472.

157. Hahn, A.W.; Regenass, S.; Resink, T.J.; Kern, F.; Bühler, F.R. Morphogenic effects of endothelin-1 on vascular smooth muscle cells. *J. Vasc. Res.* **1993**, *30*, 192–201.
158. Planas-Rigol, E.; Terrades-Garcia, N.; Corbera-Bellalta, M.; Lozano, E.; Alba, M.A.; Segarra, M.; Espígol-Frigolé, G.; Prieto-González, S.; Hernández-Rodríguez, J.; Preciado, S.; et al. Endothelin-1 promotes vascular smooth muscle cell migration across the artery wall: A mechanism contributing to vascular remodelling and intimal hyperplasia in giant-cell arteritis. *Ann. Rheum. Dis.* **2017**, *76*, 1624–1634.
159. Meoli, D.F.; White, R.J. Endothelin-1 induces pulmonary but not aortic smooth muscle cell migration by activating ERK1/2 MAP kinase. *Can. J. Physiol. Pharmacol.* **2010**, *88*, 830–839.
160. Kashima, Y.; Takahashi, M.; Shiba, Y.; Itano, N.; Izawa, A.; Koyama, J.; Nakayama, J.; Taniguchi, S.; Kimata, K.; Ikeda, U. Crucial role of hyaluronan in neointimal formation after vascular injury. *PLoS ONE* **2013**, *8*, e58760.
161. Vendrov, A.E.; Madamanchi, N.R.; Niu, X.-L.; Molnar, K.C.; Runge, M.; Szyndralewiez, C.; Page, P.; Runge, M.S. NADPH oxidases regulate CD44 and hyaluronic acid expression in thrombin-treated vascular smooth muscle cells and in atherosclerosis. *J. Biol. Chem.* **2010**, *285*, 26545–26557.
162. Fischer, J.W. Role of hyaluronan in atherosclerosis: Current knowledge and open questions. *Matrix Biol. J. Int. Soc. Matrix Biol.* **2019**, *78–79*, 324–336.
163. Wilkinson, T.S.; Bressler, S.L.; Evanko, S.P.; Braun, K.R.; Wight, T.N. Overexpression of hyaluronan synthases alters vascular smooth muscle cell phenotype and promotes monocyte adhesion. *J. Cell. Physiol.* **2006**, *206*, 378–385.
164. Grandoch, M.; Hoffmann, J.; Röck, K.; Wenzel, F.; Oberhuber, A.; Schelzig, H.; Fischer, J.W. Novel effects of adenosine receptors on pericellular hyaluronan matrix: Implications for human smooth muscle cell phenotype and interactions with monocytes during atherosclerosis. *Basic Res. Cardiol.* **2013**, *108*, 340.
165. Sadowitz, B.; Seymour, K.; Gahtan, V.; Maier, K.G. The role of hyaluronic acid in atherosclerosis and intimal hyperplasia. *J. Surg. Res.* **2012**, *173*, e63–e72.
166. Moretto, P.; Karousou, E.; Viola, M.; Caon, I.; D'Angelo, M.L.; De Luca, G.; Passi, A.; Vigetti, D. Regulation of hyaluronan synthesis in vascular diseases and diabetes. *J. Diabetes Res.* **2015**, *2015*, 167283.
167. Lorentzen, K.A.; Chai, S.; Chen, H.; Danielsen, C.C.; Simonsen, U.; Wogensen, L. Mechanisms involved in extracellular matrix remodeling and arterial stiffness induced by hyaluronan accumulation. *Atherosclerosis* **2016**, *244*, 195–203.
168. Evanko, S.P.; Angello, J.C.; Wight, T.N. Formation of hyaluronan- and versican-rich pericellular matrix is required for proliferation and migration of vascular smooth muscle cells. *Arterioscler. Thromb. Vasc. Biol.* **1999**, *19*, 1004–1013.
169. Svensson Holm, A.-C.B.; Bengtsson, T.; Grenegård, M.; Lindström, E.G. Hyaluronic acid influence on platelet-induced airway smooth muscle cell proliferation. *Exp. Cell Res.* **2012**, *318*, 632–640.
170. Stein, J.J.; Iwuchukwu, C.; Maier, K.G.; Gahtan, V. Thrombospondin-1-induced smooth muscle cell chemotaxis and proliferation are dependent on transforming growth factor-β2 and hyaluronic acid synthase. *Mol. Cell. Biochem.* **2013**, *384*, 181–186.
171. Papakonstantinou, E.; Karakiulakis, G.; Eickelberg, O.; Perruchoud, A.P.; Block, L.H.; Roth, M. A 340 kDa hyaluronic acid secreted by human vascular smooth muscle cells regulates their proliferation and migration. *Glycobiology* **1998**, *8*, 821–830.
172. Papakonstantinou, E.; Roth, M.; Block, L.H.; Mirtsou-Fidani, V.; Argiriadis, P.; Karakiulakis, G. The differential distribution of hyaluronic acid in the layers of human atheromatic aortas is associated with vascular smooth muscle cell proliferation and migration. *Atherosclerosis* **1998**, *138*, 79–89.
173. Kothapalli, D.; Flowers, J.; Xu, T.; Puré, E.; Assoian, R.K. Differential activation of ERK and Rac mediates the proliferative and anti-proliferative effects of hyaluronan and CD44. *J. Biol. Chem.* **2008**, *283*, 31823–31829.
174. Boudreau, N.; Turley, E.; Rabinovitch, M. Fibronectin, hyaluronan, and a hyaluronan binding protein contribute to increased ductus arteriosus smooth muscle cell migration. *Dev. Biol.* **1991**, *143*, 235–247.
175. Maier, K.G.; Sadowitz, B.; Cullen, S.; Han, X.; Gahtan, V. Thrombospondin-1-induced vascular smooth muscle cell migration is dependent on the hyaluronic acid receptor CD44. *Am. J. Surg.* **2009**, *198*, 664–669.
176. Gouëffic, Y.; Guilluy, C.; Guérin, P.; Patra, P.; Pacaud, P.; Loirand, G. Hyaluronan induces vascular smooth muscle cell migration through RHAMM-mediated PI3K-dependent Rac activation. *Cardiovasc. Res.* **2006**, *72*, 339–348.

177. Savani, R.C.; Wang, C.; Yang, B.; Zhang, S.; Kinsella, M.G.; Wight, T.N.; Stern, R.; Nance, D.M.; Turley, E.A. Migration of bovine aortic smooth muscle cells after wounding injury. The role of hyaluronan and RHAMM. *J. Clin. Invest.* **1995**, *95*, 1158–1168.
178. Bot, P.T.; Pasterkamp, G.; Goumans, M.-J.; Strijder, C.; Moll, F.L.; de Vries, J.-P.; Pals, S.T.; de Kleijn, D.P.; Piek, J.J.; Hoefer, I.E. Hyaluronic acid metabolism is increased in unstable plaques. *Eur. J. Clin. Invest.* **2010**, *40*, 818–827.
179. Chai, S.; Chai, Q.; Danielsen, C.C.; Hjorth, P.; Nyengaard, J.R.; Ledet, T.; Yamaguchi, Y.; Rasmussen, L.M.; Wogensen, L. Overexpression of hyaluronan in the tunica media promotes the development of atherosclerosis. *Circ. Res.* **2005**, *96*, 583–591.
180. Bouabdallah, J.; Zibara, K.; Issa, H.; Lenglet, G.; Kchour, G.; Caus, T.; Six, I.; Choukroun, G.; Kamel, S.; Bennis, Y. Endothelial cells exposed to phosphate and indoxyl sulphate promote vascular calcification through interleukin-8 secretion. *Nephrol. Dial. Transplant. Off. Publ. Eur. Dial. Transpl. Assoc.—Eur. Ren. Assoc.* **2019**, *34*, 1125–1134.
181. Raaz-Schrauder, D.; Klinghammer, L.; Baum, C.; Frank, T.; Lewczuk, P.; Achenbach, S.; Cicha, I.; Stumpf, C.; Wiltfang, J.; Kornhuber, J.; et al. Association of systemic inflammation markers with the presence and extent of coronary artery calcification. *Cytokine* **2012**, *57*, 251–257.
182. Gauss, S.; Klinghammer, L.; Steinhoff, A.; Raaz-Schrauder, D.; Marwan, M.; Achenbach, S.; Garlichs, C.D. Association of systemic inflammation with epicardial fat and coronary artery calcification. *Inflamm. Res. Off. J. Eur. Histamine Res. Soc. Al* **2015**, *64*, 313–319.
183. Hastings, N.E.; Feaver, R.E.; Lee, M.Y.; Wamhoff, B.R.; Blackman, B.R. Human IL-8 regulates smooth muscle cell VCAM-1 expression in response to endothelial cells exposed to atheroprone flow. *Arterioscler. Thromb. Vasc. Biol.* **2009**, *29*, 725–731.
184. Kim, J.H.; Kang, Y.J.; Kim, H.S. IL-8/CXCL8 Upregulates 12-Lipoxygenase Expression in Vascular Smooth Muscle Cells from Spontaneously Hypertensive Rats. *Immune Netw.* **2009**, *9*, 106–113.
185. Kuo, P.-L.; Hsu, Y.-L.; Huang, M.-S.; Chiang, S.-L.; Ko, Y.-C. Bronchial epithelium-derived IL-8 and RANTES increased bronchial smooth muscle cell migration and proliferation by Krüppel-like factor 5 in areca nut-mediated airway remodeling. *Toxicol. Sci. Off. J. Soc. Toxicol.* **2011**, *121*, 177–190.
186. Tang, H.; Sun, Y.; Shi, Z.; Huang, H.; Fang, Z.; Chen, J.; Xiu, Q.; Li, B. YKL-40 induces IL-8 expression from bronchial epithelium via MAPK (JNK and ERK) and NF-κB pathways, causing bronchial smooth muscle proliferation and migration. *J. Immunol. Baltim. Md 1950* **2013**, *190*, 438–446.
187. Meng, C.; Qi, X.; Li, Y.; Dang, Y.; Liu, H.; Wang, T.; Yuan, H.; Ni, Y.; Liu, G.; Wu, Z.; et al. [Interleukin-8 monoclonal antibody attenuates smooth muscle cell proliferation and balloon inflation-induced abdominal aorta stenosis in rabbits]. *Zhonghua Xin Xue Guan Bing Za Zhi* **2012**, *40*, 1056–1061.
188. Kuo, P.-L.; Hsu, Y.-L.; Tsai, M.-J.; Lien, C.-T.; Huang, M.-S.; Ko, Y.-C. Nonylphenol induces bronchial epithelial apoptosis via Fas-mediated pathway and stimulates bronchial epithelium to secrete IL-6 and IL-8, causing bronchial smooth muscle proliferation and migration. *Basic Clin. Pharmacol. Toxicol.* **2012**, *110*, 178–186.
189. Yue, T.L.; Wang, X.; Sung, C.P.; Olson, B.; McKenna, P.J.; Gu, J.L.; Feuerstein, G.Z. Interleukin-8. A mitogen and chemoattractant for vascular smooth muscle cells. *Circ. Res.* **1994**, *75*, 1–7.
190. Qin, Y.; Fan, F.; Zhao, Y.; Cui, Y.; Wei, X.; Kohama, K.; Gordon, J.R.; Li, F.; Gao, Y. Recombinant human CXCL8(3-72)K11R/G31P regulates smooth muscle cell proliferation and migration through blockage of interleukin-8 receptor. *IUBMB Life* **2013**, *65*, 67–75.
191. Lv, G.; Zhu, H.; Li, C.; Wang, J.; Zhao, D.; Li, S.; Ma, L.; Sun, G.; Li, F.; Zhao, Y.; et al. Inhibition of IL-8-mediated endothelial adhesion, VSMCs proliferation and migration by siRNA-TMEM98 suggests TMEM98's emerging role in atherosclerosis. *Oncotarget* **2017**, *8*, 88043–88058.
192. Govindaraju, V.; Michoud, M.-C.; Al-Chalabi, M.; Ferraro, P.; Powell, W.S.; Martin, J.G. Interleukin-8: Novel roles in human airway smooth muscle cell contraction and migration. *Am. J. Physiol. Cell Physiol.* **2006**, *291*, C957–C965.
193. Yue, T.L.; Mckenna, P.J.; Gu, J.L.; Feuerstein, G.Z. Interleukin-8 is chemotactic for vascular smooth muscle cells. *Eur. J. Pharmacol.* **1993**, *240*, 81–84.
194. Lim, H.K.; Ryoo, S. Native low-density lipoprotein-dependent interleukin-8 production through pertussis toxin-sensitive G-protein coupled receptors and hydrogen peroxide generation contributes to migration of human aortic smooth muscle cells. *Yonsei Med. J.* **2011**, *52*, 413–419.
195. Meng, D.; Lv, D.-D.; Fang, J. Insulin-like growth factor-I induces reactive oxygen species production and cell migration through Nox4 and Rac1 in vascular smooth muscle cells. *Cardiovasc. Res.* **2008**, *80*, 299–308.

196. Meng, D.; Shi, X.; Jiang, B.-H.; Fang, J. Insulin-like growth factor-I (IGF-I) induces epidermal growth factor receptor transactivation and cell proliferation through reactive oxygen species. *Free Radic. Biol. Med.* **2007**, *42*, 1651–1660.
197. Sukhanov, S.; Higashi, Y.; Shai, S.-Y.; Vaughn, C.; Mohler, J.; Li, Y.; Song, Y.-H.; Titterington, J.; Delafontaine, P. IGF-1 reduces inflammatory responses, suppresses oxidative stress, and decreases atherosclerosis progression in ApoE-deficient mice. *Arterioscler. Thromb. Vasc. Biol.* **2007**, *27*, 2684–2690.
198. Sukhanov, S.; Higashi, Y.; Shai, S.-Y.; Blackstock, C.; Galvez, S.; Vaughn, C.; Titterington, J.; Delafontaine, P. Differential requirement for nitric oxide in IGF-1-induced anti-apoptotic, anti-oxidant and anti-atherosclerotic effects. *FEBS Lett.* **2011**, *585*, 3065–3072.
199. Stratton, M.S.; Yang, X.; Sreejayan, N.; Ren, J. Impact of insulin-like growth factor-I on migration, proliferation and Akt-ERK signaling in early and late-passages of vascular smooth muscle cells. *Cardiovasc. Toxicol.* **2007**, *7*, 273–281.
200. Allen, T.R.; Krueger, K.D.; Hunter, W.J.; Agrawal, D.K. Evidence that insulin-like growth factor-1 requires protein kinase C-epsilon, PI3-kinase and mitogen-activated protein kinase pathways to protect human vascular smooth muscle cells from apoptosis. *Immunol. Cell Biol.* **2005**, *83*, 651–667.
201. Bai, H. z; Pollman, M.J.; Inishi, Y.; Gibbons, G.H. Regulation of vascular smooth muscle cell apoptosis. Modulation of bad by a phosphatidylinositol 3-kinase-dependent pathway. *Circ. Res.* **1999**, *85*, 229–237.
202. Sun, M.; Wang, F.; Feng, P. Insulin-like growth factor-1 inhibits colonic smooth muscle cell apoptosis in diabetic rats with colonic dysmotility. *Regul. Pept.* **2014**, *194–195*, 41–48.
203. Zwaka, T.P.; Torzewski, J.; Hoeflich, A.; Déjosez, M.; Kaiser, S.; Hombach, V.; Jehle, P.M. The terminal complement complex inhibits apoptosis in vascular smooth muscle cells by activating an autocrine IGF-1 loop. *FASEB J. Off. Publ. Fed. Am. Soc. Exp. Biol.* **2003**, *17*, 1346–1348.
204. González-Timón, B.; González-Muñoz, M.; Zaragoza, C.; Lamas, S.; Melián, E.M. Native and oxidized low density lipoproteins oppositely modulate the effects of insulin-like growth factor I on VSMC. *Cardiovasc. Res.* **2004**, *61*, 247–255.
205. Jin, C.; Guo, J.; Qiu, X.; Ma, K.; Xiang, M.; Zhu, X.; Guo, J. IGF-1 induces iNOS expression via the p38 MAPK signal pathway in the anti-apoptotic process in pulmonary artery smooth muscle cells during PAH. *J. Recept. Signal Transduct. Res.* **2014**, *34*, 325–331.
206. Kuemmerle, J.F.; Zhou, H.; Bowers, J.G. IGF-I stimulates human intestinal smooth muscle cell growth by regulation of G1 phase cell cycle proteins. *Am. J. Physiol. Gastrointest. Liver Physiol.* **2004**, *286*, G412–G419.
207. Myit, S.; Delafontaine, P.; Bochaton-Piallat, M.-L.; Giraud, S.; Gabbiani, G.; Brink, M. Different growth properties of neointimal and medial smooth muscle cells in response to growth factors. *J. Vasc. Res.* **2003**, *40*, 97–104.
208. Chen, Y.; Capron, L.; Magnusson, J.O.; Wallby, L.A.; Arnqvist, H.J. Insulin-like growth factor-1 stimulates vascular smooth muscle cell proliferation in rat aorta in vivo. *Growth Horm. IGF Res. Off. J. Growth Horm. Res. Soc. Int. IGF Res. Soc.* **1998**, *8*, 299–303.
209. Yano, K.; Bauchat, J.R.; Liimatta, M.B.; Clemmons, D.R.; Duan, C. Down-regulation of protein kinase C inhibits insulin-like growth factor I-induced vascular smooth muscle cell proliferation, migration, and gene expression. *Endocrinology* **1999**, *140*, 4622–4632.
210. Cheng, G.; Kim, M.-J.; Jia, G.; Agrawal, D.K. Involvement of chloride channels in IGF-I-induced proliferation of porcine arterial smooth muscle cells. *Cardiovasc. Res.* **2007**, *73*, 198–207.
211. Imai, Y.; Clemmons, D.R. Roles of phosphatidylinositol 3-kinase and mitogen-activated protein kinase pathways in stimulation of vascular smooth muscle cell migration and deoxyriboncleic acid synthesis by insulin-like growth factor-I. *Endocrinology* **1999**, *140*, 4228–4235.
212. Allen, L.B.; Capps, B.E.; Miller, E.C.; Clemmons, D.R.; Maile, L.A. Glucose-oxidized low-density lipoproteins enhance insulin-like growth factor I-stimulated smooth muscle cell proliferation by inhibiting integrin-associated protein cleavage. *Endocrinology* **2009**, *150*, 1321–1329.
213. Duan, C.; Bauchat, J.R.; Hsieh, T. Phosphatidylinositol 3-kinase is required for insulin-like growth factor-I-induced vascular smooth muscle cell proliferation and migration. *Circ. Res.* **2000**, *86*, 15–23.
214. González, B.; Lamas, S.; Melián, E.M. Cooperation between low density lipoproteins and IGF-I in the promotion of mitogenesis in vascular smooth muscle cells. *Endocrinology* **2001**, *142*, 4852–4860.
215. Bayes-Genis, A.; Schwartz, R.S.; Bale, L.K.; Conover, C.A. Effects of insulin-like growth factor-I on cultured human coronary artery smooth muscle cells. *Growth Horm. IGF Res. Off. J. Growth Horm. Res. Soc. Int. IGF Res. Soc.* **2003**, *13*, 246–253.

216. Wang, L.; Han, Y.; Shen, Y.; Yan, Z.-Q.; Zhang, P.; Yao, Q.-P.; Shen, B.-R.; Gao, L.-Z.; Qi, Y.-X.; Jiang, Z.-L. Endothelial insulin-like growth factor-1 modulates proliferation and phenotype of smooth muscle cells induced by low shear stress. *Ann. Biomed. Eng.* **2014**, *42*, 776–786.
217. Li, K.; Wang, Y.; Zhang, A.; Liu, B.; Jia, L. miR-379 Inhibits Cell Proliferation, Invasion, and Migration of Vascular Smooth Muscle Cells by Targeting Insulin-Like Factor-1. *Yonsei Med. J.* **2017**, *58*, 234–240.
218. Kou, X.; Han, Y.; Yang, D.; Liu, Y.; Fu, J.; Zheng, S.; He, D.; Zhou, L.; Zeng, C. Dopamine d(1)-like receptors suppress proliferation of vascular smooth muscle cell induced by insulin-like growth factor-1. *Clin. Exp. Hypertens. N. Y. N 1993* **2014**, *36*, 140–147.
219. Shuang, T.; Fu, M.; Yang, G.; Wu, L.; Wang, R. The interaction of IGF-1/IGF-1R and hydrogen sulfide on the proliferation of mouse primary vascular smooth muscle cells. *Biochem. Pharmacol.* **2018**, *149*, 143–152.
220. Pukac, L.; Huangpu, J.; Karnovsky, M.J. Platelet-derived growth factor-BB, insulin-like growth factor-I, and phorbol ester activate different signaling pathways for stimulation of vascular smooth muscle cell migration. *Exp. Cell Res.* **1998**, *242*, 548–560.
221. Bornfeldt, K.E.; Raines, E.W.; Nakano, T.; Graves, L.M.; Krebs, E.G.; Ross, R. Insulin-like growth factor-I and platelet-derived growth factor-BB induce directed migration of human arterial smooth muscle cells via signaling pathways that are distinct from those of proliferation. *J. Clin. Invest.* **1994**, *93*, 1266–1274.
222. Ruiz-Torres, A.; Lozano, R.; Melón, J.; Carraro, R. Age-dependent decline of in vitro migration (basal and stimulated by IGF-1 or insulin) of human vascular smooth muscle cells. *J. Gerontol. A. Biol. Sci. Med. Sci.* **2003**, *58*, B1074–B1077.
223. Beneit, N.; Fernández-García, C.E.; Martín-Ventura, J.L.; Perdomo, L.; Escribano, Ó.; Michel, J.B.; García-Gómez, G.; Fernández, S.; Díaz-Castroverde, S.; Egido, J.; et al. Expression of insulin receptor (IR) A and B isoforms, IGF-IR, and IR/IGF-IR hybrid receptors in vascular smooth muscle cells and their role in cell migration in atherosclerosis. *Cardiovasc. Diabetol.* **2016**, *15*, 161.
224. Jia, G.; Cheng, G.; Gangahar, D.M.; Agrawal, D.K. Insulin-like growth factor-1 and TNF-alpha regulate autophagy through c-jun N-terminal kinase and Akt pathways in human atherosclerotic vascular smooth cells. *Immunol. Cell Biol.* **2006**, *84*, 448–454.
225. Okura, Y.; Brink, M.; Zahid, A.A.; Anwar, A.; Delafontaine, P. Decreased expression of insulin-like growth factor-1 and apoptosis of vascular smooth muscle cells in human atherosclerotic plaque. *J. Mol. Cell. Cardiol.* **2001**, *33*, 1777–1789.
226. von der Thüsen, J.H.; Borensztajn, K.S.; Moimas, S.; van Heiningen, S.; Teeling, P.; van Berkel, T.J.C.; Biessen, E.A.L. IGF-1 has plaque-stabilizing effects in atherosclerosis by altering vascular smooth muscle cell phenotype. *Am. J. Pathol.* **2011**, *178*, 924–934.
227. Shai, S.-Y.; Sukhanov, S.; Higashi, Y.; Vaughn, C.; Kelly, J.; Delafontaine, P. Smooth muscle cell-specific insulin-like growth factor-1 overexpression in Apoe-/- mice does not alter atherosclerotic plaque burden but increases features of plaque stability. *Arterioscler. Thromb. Vasc. Biol.* **2010**, *30*, 1916–1924.
228. Mallat, Z.; Heymes, C.; Ohan, J.; Faggin, E.; Lesèche, G.; Tedgui, A. Expression of interleukin-10 in advanced human atherosclerotic plaques: Relation to inducible nitric oxide synthase expression and cell death. *Arterioscler. Thromb. Vasc. Biol.* **1999**, *19*, 611–616.
229. Potteaux, S.; Esposito, B.; van Oostrom, O.; Brun, V.; Ardouin, P.; Groux, H.; Tedgui, A.; Mallat, Z. Leukocyte-derived interleukin 10 is required for protection against atherosclerosis in low-density lipoprotein receptor knockout mice. *Arterioscler. Thromb. Vasc. Biol.* **2004**, *24*, 1474–1478.
230. Rajagopal, S.P.; Hutchinson, J.L.; Dorward, D.A.; Rossi, A.G.; Norman, J.E. Crosstalk between monocytes and myometrial smooth muscle in culture generates synergistic pro-inflammatory cytokine production and enhances myocyte contraction, with effects opposed by progesterone. *Mol. Hum. Reprod.* **2015**, *21*, 672–686.
231. Mazighi, M.; Pellé, A.; Gonzalez, W.; Mtairag, E.M.; Philippe, M.; Hénin, D.; Michel, J.-B.; Feldman, L.J. IL-10 inhibits vascular smooth muscle cell activation in vitro and in vivo. *Am. J. Physiol. Heart Circ. Physiol.* **2004**, *287*, H866–H871.
232. Liu, H.; Yang, S.; Sun, X.; Chen, T. Human interleukin-10 gene inhibits acute rejection by triggering apoptosis in allograft vascular transplantation. *Int. J. Clin. Exp. Pathol.* **2014**, *7*, 5864–5871.

233. Ito, T.; Okada, T.; Miyashita, H.; Nomoto, T.; Nonaka-Sarukawa, M.; Uchibori, R.; Maeda, Y.; Urabe, M.; Mizukami, H.; Kume, A.; et al. Interleukin-10 expression mediated by an adeno-associated virus vector prevents monocrotaline-induced pulmonary arterial hypertension in rats. *Circ. Res.* **2007**, *101*, 734–741.
234. Selzman, C.H.; Shames, B.D.; Whitehill, T.A.; Harken, A.H.; McIntyre, R.C. Class II cytokine receptor ligands inhibit human vascular smooth muscle proliferation. *Surgery* **1998**, *124*, 318–326; discussion 326-327.
235. Xia, C.; Huo, Y.; Yin, H.; Zhu, G.; Tang, C. Interleukin 10 inhibits the rat VSMC proliferation and collagen secretion stimulated by angiotensin II. *Chin. Med. Sci. J. Chung-Kuo Hsueh Ko Hsueh Tsa Chih* **2001**, *16*, 125–128.
236. Ouyang, P.; Peng, L.-S.; Yang, H.; Peng, W.-L.; Wu, W.-Y.; Xu, A.-L. Recombinant human interleukin-10 inhibits proliferation of vascular smooth muscle cells stimulated by advanced glycation end products and neointima hyperplasia after carotid injury in the rat. *Sheng Li Xue Bao* **2003**, *55*, 128–134.
237. Selzman, C.H.; Meldrum, D.R.; Cain, B.S.; Meng, X.; Shames, B.D.; Ao, L.; Harken, A.H. Interleukin-10 inhibits postinjury tumor necrosis factor-mediated human vascular smooth muscle proliferation. *J. Surg. Res.* **1998**, *80*, 352–356.
238. Selzman, C.H.; McIntyre, R.C.; Shames, B.D.; Whitehill, T.A.; Banerjee, A.; Harken, A.H. Interleukin-10 inhibits human vascular smooth muscle proliferation. *J. Mol. Cell. Cardiol.* **1998**, *30*, 889–896.
239. Kim, H.Y.; Kim, H.S. Dimethylarginine dimethylaminohydrolase-1 mediates inhibitory effect of interleukin-10 on angiotensin II-induced hypertensive effects in vascular smooth muscle cells of spontaneously hypertensive rats. *Cytokine* **2016**, *77*, 203–210.
240. Zernecke, A.; Liehn, E.A.; Gao, J.-L.; Kuziel, W.A.; Murphy, P.M.; Weber, C. Deficiency in CCR5 but not CCR1 protects against neointima formation in atherosclerosis-prone mice: Involvement of IL-10. *Blood* **2006**, *107*, 4240–4243.
241. Rectenwald, J.E.; Minter, R.M.; Moldawer, L.L.; Abouhamze, Z.; La Face, D.; Hutchins, E.; Huber, T.S.; Seeger, J.M.; Ozaki, C.K. Interleukin-10 fails to modulate low shear stress-induced neointimal hyperplasia. *J. Surg. Res.* **2002**, *102*, 110–118.
242. Sage, A.P.; Nus, M.; Baker, L.L.; Finigan, A.J.; Masters, L.M.; Mallat, Z. Regulatory B cell-specific interleukin-10 is dispensable for atherosclerosis development in mice. *Arterioscler. Thromb. Vasc. Biol.* **2015**, *35*, 1770–1773.
243. Schelski, N., Luong, T.T.D.; Lang. F.; Pieske. B.; Voelkl, J., Alesutan, I., SGK1-dependent Stimulation of Vascular Smooth Muscle Cell Osteo-/Chondrogenic Transdifferentiation by interleukin-18 Available online: https://pubmed.ncbi.nlm.nih.gov/30706178/?from_term=il-18+AND+calcification&from_size=50&from_pos=7 (accessed on Mar 19, 2020).
244. Zhang, K.; Zhang, Y.; Feng, W.; Chen, R.; Chen, J.; Touyz, R.M.; Wang, J.; Huang, H. Interleukin-18 Enhances Vascular Calcification and Osteogenic Differentiation of Vascular Smooth Muscle Cells Through TRPM7 Activation. *Arterioscler. Thromb. Vasc. Biol.* **2017**, *37*, 1933–1943.
245. Wang, B.; Wei, G.; Liu, B.; Zhou, X.; Xiao, H.; Dong, N.; Li, F. The Role of High Mobility Group Box 1 Protein in Interleukin-18-Induced Myofibroblastic Transition of Valvular Interstitial Cells. *Cardiology* **2016**, *135*, 168–178.
246. Porażko, T., Kuźniar, J., Kusztal, M., Kuźniar, T. J., Weyde, W., Kuriata-Kordek, M., Klinger, M, IL-18 Is Involved in Vascular Injury in End-Stage Renal Disease Patients Available online: https://pubmed.ncbi.nlm.nih.gov/18775894/?from_term=il-18+AND+calcification&from_size=50&from_pos=9 (accessed on Mar 19, 2020).
247. Kiu Weber, C. I., Duchateau-Nguyen, G., Solier, C., Schell-Steven, A., Hermosilla, R., Nogoceke, E., Block, G, Cardiovascular Risk Markers Associated With Arterial Calcification in Patients With Chronic Kidney Disease Stages 3 and 4 Available online: https://pubmed.ncbi.nlm.nih.gov/24683472/?from_term=il-18+AND+calcification&from_size=50&from_pos=17 (accessed on Mar 19, 2020).
248. Venkatesan, B.; Valente, A.J.; Reddy, V.S.; Siwik, D.A.; Chandrasekar, B. Resveratrol blocks interleukin-18-EMMPRIN cross-regulation and smooth muscle cell migration. *Am. J. Physiol. Heart Circ. Physiol.* **2009**, *297*, H874–H886.
249. Rodriguez-Menocal, L.; Faridi, M.H.; Martinez, L.; Shehadeh, L.A.; Duque, J.C.; Wei, Y.; Mesa, A.; Pena, A.; Gupta, V.; Pham, S.M.; et al. Macrophage-derived IL-18 and increased fibrinogen deposition are age-related inflammatory signatures of vascular remodeling. *Am. J. Physiol. Heart Circ. Physiol.* **2014**, *306*, H641–H653.

250. Valente, A.J.; Yoshida, T.; Murthy, S.N.; Sakamuri, S.S.V.P.; Katsuyama, M.; Clark, R.A.; Delafontaine, P.; Chandrasekar, B. Angiotensin II enhances AT1-Nox1 binding and stimulates arterial smooth muscle cell migration and proliferation through AT1, Nox1, and interleukin-18. *Am. J. Physiol. Heart Circ. Physiol.* **2012**, *303*, H282–H296.
251. Zhang, S.; Luo, X.; Huang, H.; Chai, Y.; Hu, D.; Tao, Q. [N-acetylcysteine antagonizes the Interleukin-18-induced expression of TNF-alpha and IL-6 in mouse vascular smooth muscle cells]. *Xi Bao Yu Fen Zi Mian Yi Xue Za Zhi Chin. J. Cell. Mol. Immunol.* **2010**, *26*, 35–37.
252. Sahar, S.; Dwarakanath, R.S.; Reddy, M.A.; Lanting, L.; Todorov, I.; Natarajan, R. Angiotensin II enhances interleukin-18 mediated inflammatory gene expression in vascular smooth muscle cells: A novel cross-talk in the pathogenesis of atherosclerosis. *Circ. Res.* **2005**, *96*, 1064–1071.
253. Gerdes, N.; Sukhova, G.K.; Libby, P.; Reynolds, R.S.; Young, J.L.; Schönbeck, U. Expression of interleukin (IL)-18 and functional IL-18 receptor on human vascular endothelial cells, smooth muscle cells, and macrophages: Implications for atherogenesis. *J. Exp. Med.* **2002**, *195*, 245–257.
254. Chandrasekar, B.; Mummidi, S.; Valente, A.J.; Patel, D.N.; Bailey, S.R.; Freeman, G.L.; Hatano, M.; Tokuhisa, T.; Jensen, L.E. The pro-atherogenic cytokine interleukin-18 induces CXCL16 expression in rat aortic smooth muscle cells via MyD88, interleukin-1 receptor-associated kinase, tumor necrosis factor receptor-associated factor 6, c-Src, phosphatidylinositol 3-kinase, Akt, c-Jun N-terminal kinase, and activator protein-1 signaling. *J. Biol. Chem.* **2005**, *280*, 26263–26277.
255. Maffia, P.; Grassia, G.; Di Meglio, P.; Carnuccio, R.; Berrino, L.; Garside, P.; Ianaro, A.; Ialenti, A. Neutralization of interleukin-18 inhibits neointimal formation in a rat model of vascular injury. *Circulation* **2006**, *114*, 430–437.
256. Lin, Y.-C.; Chiang, C.-H.; Chang, L.-T.; Sun, C.-K.; Leu, S.; Shao, P.-L.; Hsieh, M.-C.; Tsai, T.-H.; Chua, S.; Chung, S.-Y.; et al. Simvastatin attenuates the additive effects of TNF-α and IL-18 on the connexin 43 up-regulation and over-proliferation of cultured aortic smooth muscle cells. *Cytokine* **2013**, *62*, 341–351.
257. Hamakawa, T.; Sasaki, S.; Shibata, Y.; Imura, M.; Kubota, Y.; Kojima, Y.; Kohri, K. Interleukin-18 may lead to benign prostatic hyperplasia via thrombospondin-1 production in prostatic smooth muscle cells. *The Prostate* **2014**, *74*, 590–601.
258. Chandrasekar, B.; Mummidi, S.; Mahimainathan, L.; Patel, D.N.; Bailey, S.R.; Imam, S.Z.; Greene, W.C.; Valente, A.J. Interleukin-18-induced human coronary artery smooth muscle cell migration is dependent on NF-kappaB- and AP-1-mediated matrix metalloproteinase-9 expression and is inhibited by atorvastatin. *J. Biol. Chem.* **2006**, *281*, 15099–15109.
259. Valente, A.J.; Yoshida, T.; Izadpanah, R.; Delafontaine, P.; Siebenlist, U.; Chandrasekar, B. Interleukin-18 enhances IL-18R/Nox1 binding, and mediates TRAF3IP2-dependent smooth muscle cell migration. Inhibition by simvastatin. *Cell. Signal.* **2013**, *25*, 1447–1456.
260. Elhage, R.; Jawien, J.; Rudling, M.; Ljunggren, H.-G.; Takeda, K.; Akira, S.; Bayard, F.; Hansson, G.K. Reduced atherosclerosis in interleukin-18 deficient apolipoprotein E-knockout mice. *Cardiovasc. Res.* **2003**, *59*, 234–240.
261. Tenger, C.; Sundborger, A.; Jawien, J.; Zhou, X. IL-18 accelerates atherosclerosis accompanied by elevation of IFN-gamma and CXCL16 expression independently of T cells. *Arterioscler. Thromb. Vasc. Biol.* **2005**, *25*, 791–796.
262. Pejnovic, N.; Vratimos, A.; Lee, S.H.; Popadic, D.; Takeda, K.; Akira, S.; Chan, W.L. Increased atherosclerotic lesions and Th17 in interleukin-18 deficient apolipoprotein E-knockout mice fed high-fat diet. *Mol. Immunol.* **2009**, *47*, 37–45.
263. Mallat, Z.; Corbaz, A.; Scoazec, A.; Graber, P.; Alouani, S.; Esposito, B.; Humbert, Y.; Chvatchko, Y.; Tedgui, A. Interleukin-18/interleukin-18 binding protein signaling modulates atherosclerotic lesion development and stability. *Circ. Res.* **2001**, *89*, E41–E45.
264. Pigarevskii, P.V.; Maltseva, S.V.; Snegova, V.A.; Davydova, N.G. Role of interleukin-18 in destabilization of the atherosclerotic plaque in humans. *Bull. Exp. Biol. Med.* **2014**, *157*, 821–824.
265. de Nooijer, R.; von der Thüsen, J.H.; Verkleij, C.J.N.; Kuiper, J.; Jukema, J.W.; van der Wall, E.E.; van Berkel, J.C.; Biessen, E. a. L. Overexpression of IL-18 decreases intimal collagen content and promotes a vulnerable plaque phenotype in apolipoprotein-E-deficient mice. *Arterioscler. Thromb. Vasc. Biol.* **2004**, *24*, 2313–2319.
266. Bessueille, L.; Fakhry, M.; Hamade, E.; Badran, B.; Magne, D. Glucose stimulates chondrocyte differentiation of vascular smooth muscle cells and calcification: A possible role for IL-1β. *FEBS Lett.* **2015**, *589*, 2797–2804.

267. Ceneri, N.; Zhao, L.; Young, B.D.; Healy, A.; Coskun, S.; Vasavada, H.; Yarovinsky, T.O.; Ike, K.; Pardi, R.; Qin, L.; et al. Rac2 Modulates Atherosclerotic Calcification by Regulating Macrophage IL-1β Production. *Arterioscler. Thromb. Vasc. Biol.* **2017**, *37*, 328–340.
268. Han, L.; Zhang, Y.; Zhang, M.; Guo, L.; Wang, J.; Zeng, F.; Xu, D.; Yin, Z.; Xu, Y.; Wang, D.; et al. Interleukin-1β-Induced Senescence Promotes Osteoblastic Transition of Vascular Smooth Muscle Cells. *Kidney Blood Press. Res.* **2020**, *45*, 314–330.
269. Dautova, Y.; Kapustin, A.N.; Pappert, K.; Epple, M.; Okkenhaug, H.; Cook, S.J.; Shanahan, C.M.; Bootman, M.D.; Proudfoot, D. Calcium phosphate particles stimulate interleukin-1β release from human vascular smooth muscle cells: A role for spleen tyrosine kinase and exosome release. *J. Mol. Cell. Cardiol.* **2018**, *115*, 82–93.
270. Agharazii, M.; St-Louis, R.; Gautier-Bastien, A.; Ung, R.-V.; Mokas, S.; Larivière, R.; Richard, D.E. Inflammatory cytokines and reactive oxygen species as mediators of chronic kidney disease-related vascular calcification. *Am. J. Hypertens.* **2015**, *28*, 746–755.
271. Awan, Z.; Denis, M.; Roubtsova, A.; Essalmani, R.; Marcinkiewicz, J.; Awan, A.; Gram, H.; Seidah, N.G.; Genest, J. Reducing Vascular Calcification by Anti-IL-1β Monoclonal Antibody in a Mouse Model of Familial Hypercholesterolemia. *Angiology* **2016**, *67*, 157–167.
272. Kraśniak, A.; Drozdz, M.; Pasowicz, M.; Chmiel, G.; Michałek, M.; Szumilak, D.; Podolec, P.; Klimeczek, P.; Konieczyńska, M.; Wicher-Muniak, E.; et al. Factors involved in vascular calcification and atherosclerosis in maintenance haemodialysis patients. *Nephrol. Dial. Transplant. Off. Publ. Eur. Dial. Transpl. Assoc.—Eur. Ren. Assoc.* **2007**, *22*, 515–521.
273. Ikeda, K.; Souma, Y.; Akakabe, Y.; Kitamura, Y.; Matsuo, K.; Shimoda, Y.; Ueyama, T.; Matoba, S.; Yamada, H.; Okigaki, M.; et al. Macrophages play a unique role in the plaque calcification by enhancing the osteogenic signals exerted by vascular smooth muscle cells. *Biochem. Biophys. Res. Commun.* **2012**, *425*, 39–44.
274. Hoare, G.S.; Marczin, N.; Chester, A.H.; Yacoub, M.H. Role of oxidant stress in cytokine-induced activation of NF-kappaB in human aortic smooth muscle cells. *Am. J. Physiol.* **1999**, *277*, H1975–H1984.
275. Alexander, M.R.; Murgai, M.; Moehle, C.W.; Owens, G.K. Interleukin-1β modulates smooth muscle cell phenotype to a distinct inflammatory state relative to PDGF-DD via NF-κB-dependent mechanisms. *Physiol. Genomics* **2012**, *44*, 417–429.
276. Lim, J.H.; Um, H.J.; Park, J.-W.; Lee, I.-K.; Kwon, T.K. Interleukin-1beta promotes the expression of monocyte chemoattractant protein-1 in human aorta smooth muscle cells via multiple signaling pathways. *Exp. Mol. Med.* **2009**, *41*, 757–764.
277. Braun, M.; Pietsch, P.; Zepp, A.; Schrör, K.; Baumann, G.; Felix, S.B. Regulation of tumor necrosis factor alpha- and interleukin-1-beta-induced induced adhesion molecule expression in human vascular smooth muscle cells by cAMP. *Arterioscler. Thromb. Vasc. Biol.* **1997**, *17*, 2568–2575.
278. Wang, X.; Feuerstein, G.Z.; Gu, J.L.; Lysko, P.G.; Yue, T.L. Interleukin-1 beta induces expression of adhesion molecules in human vascular smooth muscle cells and enhances adhesion of leukocytes to smooth muscle cells. *Atherosclerosis* **1995**, *115*, 89–98.
279. Wang, X.; Feuerstein, G.Z.; Clark, R.K.; Yue, T.L. Enhanced leucocyte adhesion to interleukin-1 beta stimulated vascular smooth muscle cells is mainly through intercellular adhesion molecule-1. *Cardiovasc. Res.* **1994**, *28*, 1808–1814.
280. Vromman, A.; Trabelsi, N.; Rouxel, C.; Béréziat, G.; Limon, I.; Blaise, R. β-Amyloid context intensifies vascular smooth muscle cells induced inflammatory response and de-differentiation. *Aging Cell* **2013**, *12*, 358–369.
281. Clément, N.; Gueguen, M.; Glorian, M.; Blaise, R.; Andréani, M.; Brou, C.; Bausero, P.; Limon, I. Notch3 and IL-1beta exert opposing effects on a vascular smooth muscle cell inflammatory pathway in which NF-kappaB drives crosstalk. *J. Cell Sci.* **2007**, *120*, 3352–3361.
282. Clarke, M.C.H.; Talib, S.; Figg, N.L.; Bennett, M.R. Vascular smooth muscle cell apoptosis induces interleukin-1-directed inflammation: Effects of hyperlipidemia-mediated inhibition of phagocytosis. *Circ. Res.* **2010**, *106*, 363–372.
283. Geng, Y.J.; Henderson, L.E.; Levesque, E.B.; Muszynski, M.; Libby, P. Fas is expressed in human atherosclerotic intima and promotes apoptosis of cytokine-primed human vascular smooth muscle cells. *Arterioscler. Thromb. Vasc. Biol.* **1997**, *17*, 2200–2208.

284. Geng, Y.J.; Wu, Q.; Muszynski, M.; Hansson, G.K.; Libby, P. Apoptosis of vascular smooth muscle cells induced by in vitro stimulation with interferon-gamma, tumor necrosis factor-alpha, and interleukin-1 beta. *Arterioscler. Thromb. Vasc. Biol.* **1996**, *16*, 19–27.
285. Bourcier, T.; Dockter, M.; Hassid, A. Synergistic interaction of interleukin-1 beta and growth factors in primary cultures of rat aortic smooth muscle cells. *J. Cell. Physiol.* **1995**, *164*, 644–657.
286. Forsyth, E.A.; Aly, H.M.; Neville, R.F.; Sidawy, A.N. Proliferation and extracellular matrix production by human infragenicular smooth muscle cells in response to interleukin-1 beta. *J. Vasc. Surg.* **1997**, *26*, 1002–1007; discussion 1007-1008.
287. Barillari, G.; Albonici, L.; Incerpi, S.; Bogetto, L.; Pistritto, G.; Volpi, A.; Ensoli, B.; Manzari, V. Inflammatory cytokines stimulate vascular smooth muscle cells locomotion and growth by enhancing alpha5beta1 integrin expression and function. *Atherosclerosis* **2001**, *154*, 377–385.
288. Sharma, H.S.; Alagappan, V.K.T.; Willems-Widyastuti, A.; Mooi, W.J.; de Boer, W.I. Nitric oxide donors augment interleukin-1β-induced vascular endothelial growth factor in airway smooth muscle cells. *Cell Biochem. Biophys.* **2013**, *67*, 247–254.
289. Wang, X.; Chen, L.; Liu, J.; Yan, T.; Wu, G.; Xia, Y.; Zong, G.; Li, F. In vivo treatment of rat arterial adventitia with interleukin-1β induces intimal proliferation via the JAK2/STAT3 signaling pathway. *Mol. Med. Rep.* **2016**, *13*, 3451–3458.
290. Wang, J.; Razuvaev, A.; Folkersen, L.; Hedin, E.; Roy, J.; Brismar, K.; Hedin, U. The expression of IGFs and IGF binding proteins in human carotid atherosclerosis, and the possible role of IGF binding protein-1 in the regulation of smooth muscle cell proliferation. *Atherosclerosis* **2012**, *220*, 102–109.
291. Choi, H.C.; Kim, H.S.; Lee, K.Y.; Chang, K.C.; Kang, Y.J. NS-398, a selective COX-2 inhibitor, inhibits proliferation of IL-1beta-stimulated vascular smooth muscle cells by induction of HO-1. *Biochem. Biophys. Res. Commun.* **2008**, *376*, 753–757.
292. Kim, H.J.; Kim, M.Y.; Hwang, J.S.; Kim, H.J.; Lee, J.H.; Chang, K.C.; Kim, J.-H.; Han, C.W.; Kim, J.-H.; Seo, H.G. PPARdelta inhibits IL-1beta-stimulated proliferation and migration of vascular smooth muscle cells via up-regulation of IL-1Ra. *Cell. Mol. Life Sci. CMLS* **2010**, *67*, 2119–2130.
293. Li, P.; Li, Y.; Li, Z.; Wu, Y.; Zhang, C.; A., X.; Wang, C.; Shi, H.; Hui, M.; Xie, B.; et al. Cross talk between vascular smooth muscle cells and monocytes through interleukin-1β/interleukin-18 signaling promotes vein graft thickening. *Arterioscler. Thromb. Vasc. Biol.* **2014**, *34*, 2001–2011.
294. Wang, Z.; Kong, L.; Kang, J.; Vaughn, D.M.; Bush, G.D.; Walding, A.L.; Grigorian, A.A.; Robinson, J.S.; Nakayama, D.K. Interleukin-lβ induces migration of rat arterial smooth muscle cells through a mechanism involving increased matrix metalloproteinase-2 activity. *J. Surg. Res.* **2011**, *169*, 328–336.
295. Gueguen, M.; Keuylian, Z.; Mateo, V.; Mougenot, N.; Lompré, A.-M.; Michel, J.-B.; Meilhac, O.; Lipskaia, L.; Limon, I. Implication of adenylyl cyclase 8 in pathological smooth muscle cell migration occurring in rat and human vascular remodelling. *J. Pathol.* **2010**, *221*, 331–342.
296. Khan, R.; Rheaume, E.; Tardif, J.-C. Examining the Role of and Treatment Directed at IL-1β in Atherosclerosis. *Curr. Atheroscler. Rep.* **2018**, *20*, 53.
297. Eun, S.Y.; Ko, Y.S.; Park, S.W.; Chang, K.C.; Kim, H.J. IL-1β enhances vascular smooth muscle cell proliferation and migration via P2Y2 receptor-mediated RAGE expression and HMGB1 release. *Vascul. Pharmacol.* **2015**, *72*, 108–117.
298. Deuell, K.A.; Callegari, A.; Giachelli, C.M.; Rosenfeld, M.E.; Scatena, M. RANKL enhances macrophage paracrine pro-calcific activity in high phosphate-treated smooth muscle cells: Dependence on IL-6 and TNF-α. *J. Vasc. Res.* **2012**, *49*, 510–521.
299. Callegari, A.; Coons, M.L.; Ricks, J.L.; Rosenfeld, M.E.; Scatena, M. Increased Calcification in Osteoprotegerin Deficient Smooth Muscle Cells: Dependence on Receptor Activator of NF-κB Ligand and Interleukin-6. *J. Vasc. Res.* **2014**, *51*, 118–131.
300. Yao, Y.; Watson, A.D.; Ji, S.; Boström, K.I. Heat shock protein 70 enhances vascular bone morphogenetic protein-4 signaling by binding matrix Gla protein. *Circ. Res.* **2009**, *105*, 575–584.
301. Sun, M.; Chang, Q.; Xin, M.; Wang, Q.; Li, H.; Qian, J. Endogenous bone morphogenetic protein 2 plays a role in vascular smooth muscle cell calcification induced by interleukin 6 in vitro. *Int. J. Immunopathol. Pharmacol.* **2017**, *30*, 227–237.

302. Xu, D.; Zeng, F.; Han, L.; Wang, J.; Yin, Z.; Lv, L.; Guo, L.; Wang, D.; Xu, Y.; Zhou, H. The synergistic action of phosphate and interleukin-6 enhances senescence-associated calcification in vascular smooth muscle cells depending on p53. *Mech. Ageing Dev.* **2019**, *182*, 111124.
303. Kurozumi, A.; Nakano, K.; Yamagata, K.; Okada, Y.; Nakayamada, S.; Tanaka, Y. IL-6 and sIL-6R induces STAT3-dependent differentiation of human VSMCs into osteoblast-like cells through JMJD2B-mediated histone demethylation of RUNX2. *Bone* **2019**, *124*, 53–61.
304. Lee Guan-Lin; Yeh Chang-Ching; Wu Jing-Yiing; Lin Hui-Chen; Wang Yi-Fu; Kuo Ya-Yi; Hsieh Yi-Ting; Hsu Yu-Juei; Kuo Cheng-Chin TLR2 Promotes Vascular Smooth Muscle Cell Chondrogenic Differentiation and Consequent Calcification via the Concerted Actions of Osteoprotegerin Suppression and IL-6–Mediated RANKL Induction. *Arterioscler. Thromb. Vasc. Biol.* **2019**, *39*, 432–445.
305. Shavelle, D.M.; Katz, R.; Takasu, J.; Lima, J.A.; Jenny, N.S.; Budoff, M.J.; O'Brien, K.D. Soluble intercellular adhesion molecule-1 (sICAM-1) and aortic valve calcification in the multi-ethnic study of atherosclerosis (MESA). *J. Heart Valve Dis.* **2008**, *17*, 388–395.
306. Takasu, J.; Katz, R.; Shavelle, D.M.; O'Brien, K.; Mao, S.; Carr, J.J.; Cushman, M.; Budoff, M.J. Inflammation and descending thoracic aortic calcification as detected by computed tomography: The Multi-Ethnic Study of Atherosclerosis. *Atherosclerosis* **2008**, *199*, 201–206.
307. Chen, Z.; Qureshi, A.R.; Parini, P.; Hurt-Camejo, E.; Ripsweden, J.; Brismar, T.B.; Barany, P.; Jaminon, A.M.; Schurgers, L.J.; Heimbürger, O.; et al. Does statins promote vascular calcification in chronic kidney disease? *Eur. J. Clin. Invest.* **2017**, *47*, 137–148.
308. Barreto, D.V.; Barreto, F.C.; Liabeuf, S.; Temmar, M.; Lemke, H.-D.; Tribouilloy, C.; Choukroun, G.; Vanholder, R.; Massy, Z.A.; European Uremic Toxin Work Group (EUTox) Plasma interleukin-6 is independently associated with mortality in both hemodialysis and pre-dialysis patients with chronic kidney disease. *Kidney Int.* **2010**, *77*, 550–556.
309. He, J.; Reilly, M.; Yang, W.; Chen, J.; Go, A.S.; Lash, J.P.; Rahman, M.; DeFilippi, C.; Gadegbeku, C.; Kanthety, R.; et al. Risk factors for coronary artery calcium among patients with chronic kidney disease (from the Chronic Renal Insufficiency Cohort Study). *Am. J. Cardiol.* **2012**, *110*, 1735–1741.
310. Bundy, J.D.; Chen, J.; Yang, W.; Budoff, M.; Go, A.S.; Grunwald, J.E.; Kallem, R.R.; Post, W.S.; Reilly, M.P.; Ricardo, A.C.; et al. Risk factors for progression of coronary artery calcification in patients with chronic kidney disease: The CRIC study. *Atherosclerosis* **2018**, *271*, 53–60.
311. Liu, Y.; Shanahan, C.M. Signalling pathways and vascular calcification. *Front. Biosci. Landmark Ed.* **2011**, *16*, 1302–1314.
312. Wassmann, S.; Stumpf, M.; Strehlow, K.; Schmid, A.; Schieffer, B.; Böhm, M.; Nickenig, G. Interleukin-6 induces oxidative stress and endothelial dysfunction by overexpression of the angiotensin II type 1 receptor. *Circ. Res.* **2004**, *94*, 534–541.
313. den Dekker, W.K.; Tempel, D.; Bot, I.; Biessen, E.A.; Joosten, L.A.; Netea, M.G.; van der Meer, J.W.M.; Cheng, C.; Duckers, H.J. Mast cells induce vascular smooth muscle cell apoptosis via a toll-like receptor 4 activation pathway. *Arterioscler. Thromb. Vasc. Biol.* **2012**, *32*, 1960–1969.
314. Wang, Q.; Shu, C.; Su, J.; Li, X. A crosstalk triggered by hypoxia and maintained by MCP-1/miR-98/IL-6/p38 regulatory loop between human aortic smooth muscle cells and macrophages leads to aortic smooth muscle cells apoptosis via Stat1 activation. *Int. J. Clin. Exp. Pathol.* **2015**, *8*, 2670–2679.
315. Knobloch, J.; Yanik, S.D.; Körber, S.; Stoelben, E.; Jungck, D.; Koch, A. TNFα-induced airway smooth muscle cell proliferation depends on endothelin receptor signaling, GM-CSF and IL-6. *Biochem. Pharmacol.* **2016**, *116*, 188–199.
316. Lee, K.-S.; Park, J.-H.; Lee, S.; Lim, H.-J.; Choi, H.-E.; Park, H.-Y. HB-EGF induces delayed STAT3 activation via NF-kappaB mediated IL-6 secretion in vascular smooth muscle cell. *Biochim. Biophys. Acta* **2007**, *1773*, 1637–1644.
317. Xiang, S.; Dong, N.-G.; Liu, J.-P.; Wang, Y.; Shi, J.-W.; Wei, Z.-J.; Hu, X.-J.; Gong, L. Inhibitory effects of suppressor of cytokine signaling 3 on inflammatory cytokine expression and migration and proliferation of IL-6/IFN-γ-induced vascular smooth muscle cells. *J. Huazhong Univ. Sci. Technol. Med. Sci. Hua Zhong Ke Ji Xue Xue Bao Yi Xue Ying Wen Ban Huazhong Keji Daxue Xuebao Yixue Yingdewen Ban* **2013**, *33*, 615–622.
318. Yu, H.; Clarke, M.C.H.; Figg, N.; Littlewood, T.D.; Bennett, M.R. Smooth muscle cell apoptosis promotes vessel remodeling and repair via activation of cell migration, proliferation, and collagen synthesis. *Arterioscler. Thromb. Vasc. Biol.* **2011**, *31*, 2402–2409.

319. Wang, Z.; Castresana, M.R.; Newman, W.H. Reactive oxygen and NF-kappaB in VEGF-induced migration of human vascular smooth muscle cells. *Biochem. Biophys. Res. Commun.* **2001**, *285*, 669–674.
320. Wang, Z.; Newman, W.H. Smooth muscle cell migration stimulated by interleukin 6 is associated with cytoskeletal reorganization. *J. Surg. Res.* **2003**, *111*, 261–266.
321. Wang, Z.; Castresana, M.R.; Newman, W.H. NF-kappaB is required for TNF-alpha-directed smooth muscle cell migration. *FEBS Lett.* **2001**, *508*, 360–364.
322. Lee, G.-L.; Chang, Y.-W.; Wu, J.-Y.; Wu, M.-L.; Wu, K.K.; Yet, S.-F.; Kuo, C.-C. TLR 2 induces vascular smooth muscle cell migration through cAMP response element-binding protein-mediated interleukin-6 production. *Arterioscler. Thromb. Vasc. Biol.* **2012**, *32*, 2751–2760.
323. Lee, G.-L.; Wu, J.-Y.; Tsai, C.-S.; Lin, C.-Y.; Tsai, Y.-T.; Lin, C.-S.; Wang, Y.-F.; Yet, S.-F.; Hsu, Y.-J.; Kuo, C.-C. TLR4-Activated MAPK-IL-6 Axis Regulates Vascular Smooth Muscle Cell Function. *Int. J. Mol. Sci.* **2016**, *17*.
324. Sun, W.-W.; Zhu, P.; Shi, Y.-C.; Zhang, C.-L.; Huang, X.-F.; Liang, S.-Y.; Song, Z.-Y.; Lin, S. Current views on neuropeptide Y and diabetes-related atherosclerosis. *Diab. Vasc. Dis. Res.* **2017**, *14*, 277–284.
325. Crnkovic, S.; Egemnazarov, B.; Jain, P.; Seay, U.; Gattinger, N.; Marsh, L.M.; Bálint, Z.; Kovacs, G.; Ghanim, B.; Klepetko, W.; et al. NPY/Y_1 receptor-mediated vasoconstrictory and proliferative effects in pulmonary hypertension. *Br. J. Pharmacol.* **2014**, *171*, 3895–3907.
326. Pons, J.; Kitlinska, J.; Jacques, D.; Perreault, C.; Nader, M.; Everhart, L.; Zhang, Y.; Zukowska, Z. Interactions of multiple signaling pathways in neuropeptide Y-mediated bimodal vascular smooth muscle cell growth. *Can. J. Physiol. Pharmacol.* **2008**, *86*, 438–448.
327. Zukowska-Grojec, Z.; Pruszczyk, P.; Colton, C.; Yao, J.; Shen, G.H.; Myers, A.K.; Wahlestedt, C. Mitogenic effect of neuropeptide Y in rat vascular smooth muscle cells. *Peptides* **1993**, *14*, 263–268.
328. Zhong, W.; Zeng, G.; Hu, J.; Cai, Y.; Liu, J.; Huang, S.; Chen, M.; Wei, H. [Stimulation of neuropeptide Y on heat shock protein expression in renal vascular smooth muscle and the inhibition thereon by losartan]. *Zhonghua Yi Xue Za Zhi* **2003**, *83*, 515–517.
329. Erlinge, D.; Brunkwall, J.; Edvinsson, L. Neuropeptide Y stimulates proliferation of human vascular smooth muscle cells: Cooperation with noradrenaline and ATP. *Regul. Pept.* **1994**, *50*, 259–265.
330. Zhu, P.; Sun, W.; Zhang, C.; Song, Z.; Lin, S. The role of neuropeptide Y in the pathophysiology of atherosclerotic cardiovascular disease. *Int. J. Cardiol.* **2016**, *220*, 235–241.
331. Zukowska-Grojec, Z.; Karwatowska-Prokopczuk, E.; Fisher, T.A.; Ji, H. Mechanisms of vascular growth-promoting effects of neuropeptide Y: Role of its inducible receptors. *Regul. Pept.* **1998**, *75–76*, 231–238.
332. Zhang, P.; Qi, Y.-X.; Yao, Q.-P.; Chen, X.-H.; Wang, G.-L.; Shen, B.-R.; Han, Y.; Gao, L.-Z.; Jiang, Z.-L. Neuropeptide Y Stimulates Proliferation and Migration of Vascular Smooth Muscle Cells from Pregnancy Hypertensive Rats via Y1 and Y5 Receptors. *PLoS ONE* **2015**, *10*, e0131124.
333. Zhou, Y.; Shi, W.; Luo, H.; Yue, R.; Wang, Z.; Wang, W.; Liu, L.; Wang, W.E.; Wang, H.; Zeng, C. Inhibitory effect of D1-like dopamine receptors on neuropeptide Y-induced proliferation in vascular smooth muscle cells. *Hypertens. Res. Off. J. Jpn. Soc. Hypertens.* **2015**, *38*, 807–812.
334. Jiang, Z.-Q.; Zhou, Y.-L.; Chen, X.; Li, L.-Y.; Liang, S.-Y.; Lin, S.; Shu, M.-Q. Different effects of neuropeptide Y on proliferation of vascular smooth muscle cells via regulation of Geminin. *Mol. Cell. Biochem.* **2017**, *433*, 205–211.
335. Xia, X.-W.; Zhou, Y.-Q.; Luo, H.; Zeng, C. Inhibitory effect of D3 dopamine receptors on neuropeptide Y-induced migration in vascular smooth muscle cells. *Mol. Med. Rep.* **2017**, *16*, 5606–5610.
336. Li, L.; Najafi, A.H.; Kitlinska, J.B.; Neville, R.; Laredo, J.; Epstein, S.E.; Burnett, M.S.; Zukowska, Z. Of mice and men: Neuropeptide Y and its receptors are associated with atherosclerotic lesion burden and vulnerability. *J Cardiovasc. Transl. Res.* **2011**, *4*, 351–362.
337. Lagraauw, H.M.; Westra, M.M.; Bot, M.; Wezel, A.; van Santbrink, P.J.; Pasterkamp, G.; Biessen, E.A.L.; Kuiper, J.; Bot, I. Vascular neuropeptide Y contributes to atherosclerotic plaque progression and perivascular mast cell activation. *Atherosclerosis* **2014**, *235*, 196–203.
338. Carrillo-López, N.; Panizo, S.; Alonso-Montes, C.; Martínez-Arias, L.; Avello, N.; Sosa, P.; Dusso, A.S.; Cannata-Andía, J.B.; Naves-Díaz, M. High-serum phosphate and parathyroid hormone distinctly regulate bone loss and vascular calcification in experimental chronic kidney disease. *Nephrol. Dial. Transplant. Off. Publ. Eur. Dial. Transpl. Assoc.—Eur. Ren. Assoc.* **2019**, *34*, 934–941.

339. Patidar, A.; Singh, D.K.; Winocour, P.; Farrington, K.; Baydoun, A.R. Human uraemic serum displays calcific potential in vitro that increases with advancing chronic kidney disease. *Clin. Sci. Lond. Engl. 1979* **2013**, *125*, 237–245.
340. Graciolli, F.G.; Neves, K.R.; dos Reis, L.M.; Graciolli, R.G.; Noronha, I.L.; Moysés, R.M.A.; Jorgetti, V. Phosphorus overload and PTH induce aortic expression of Runx2 in experimental uraemia. *Nephrol. Dial. Transplant. Off. Publ. Eur. Dial. Transpl. Assoc.—Eur. Ren. Assoc.* **2009**, *24*, 1416–1421.
341. Neves, K.R.; Graciolli, F.G.; dos Reis, L.M.; Graciolli, R.G.; Neves, C.L.; Magalhães, A.O.; Custódio, M.R.; Batista, D.G.; Jorgetti, V.; Moysés, R.M.A. Vascular calcification: Contribution of parathyroid hormone in renal failure. *Kidney Int.* **2007**, *71*, 1262–1270.
342. Cheng, S.-L.; Shao, J.-S.; Halstead, L.R.; Distelhorst, K.; Sierra, O.; Towler, D.A. Activation of vascular smooth muscle parathyroid hormone receptor inhibits Wnt/beta-catenin signaling and aortic fibrosis in diabetic arteriosclerosis. *Circ. Res.* **2010**, *107*, 271–282.
343. Shao, J.-S.; Cheng, S.-L.; Charlton-Kachigian, N.; Loewy, A.P.; Towler, D.A. Teriparatide (human parathyroid hormone (1-34)) inhibits osteogenic vascular calcification in diabetic low density lipoprotein receptor-deficient mice. *J. Biol. Chem.* **2003**, *278*, 50195–50202.
344. Malluche, H.H.; Blomquist, G.; Monier-Faugere, M.-C.; Cantor, T.L.; Davenport, D.L. High Parathyroid Hormone Level and Osteoporosis Predict Progression of Coronary Artery Calcification in Patients on Dialysis. *J. Am. Soc. Nephrol. JASN* **2015**, *26*, 2534–2544.
345. Gulcicek, S.; Zoccali, C.; Olgun, D.Ç.; Tripepi, G.; Alagoz, S.; Yalın, S.F.; Trabulus, S.; Altiparmak, M.R.; Seyahi, N. Long-Term Progression of Coronary Artery Calcification Is Independent of Classical Risk Factors, C-Reactive Protein, and Parathyroid Hormone in Renal Transplant Patients. *Cardiorenal Med.* **2017**, *7*, 284–294.
346. Perkovic, V.; Hewitson, T.D.; Kelynack, K.J.; Martic, M.; Tait, M.G.; Becker, G.J. Parathyroid hormone has a prosclerotic effect on vascular smooth muscle cells. *Kidney Blood Press. Res.* **2003**, *26*, 27–33.
347. Sauro, M.D.; Zorn, N.E. Prolactin induces proliferation of vascular smooth muscle cells through a protein kinase C-dependent mechanism. *J. Cell. Physiol.* **1991**, *148*, 133–138.
348. Zhang, Y.; Cincotta, A.H. Inhibitory effects of bromocriptine on vascular smooth muscle cell proliferation. *Atherosclerosis* **1997**, *133*, 37–44.
349. Gan, A.M.; Pirvulescu, M.M.; Stan, D.; Simion, V.; Calin, M.; Manduteanu, I.; Butoi, E. Monocytes and smooth muscle cells cross-talk activates STAT3 and induces resistin and reactive oxygen species production [corrected]. *J. Cell. Biochem.* **2013**, *114*, 2273–2283.
350. Raghuraman, G.; Zuniga, M.C.; Yuan, H.; Zhou, W. PKCε mediates resistin-induced NADPH oxidase activation and inflammation leading to smooth muscle cell dysfunction and intimal hyperplasia. *Atherosclerosis* **2016**, *253*, 29–37.
351. Gan, A.-M.; Butoi, E.D.; Manea, A.; Simion, V.; Stan, D.; Parvulescu, M.-M.; Calin, M.; Manduteanu, I.; Simionescu, M. Inflammatory effects of resistin on human smooth muscle cells: Up-regulation of fractalkine and its receptor, CX3CR1 expression by TLR4 and Gi-protein pathways. *Cell Tissue Res.* **2013**, *351*, 161–174.
352. Cho, Y.; Lee, S.-E.; Lee, H.-C.; Hur, J.; Lee, S.; Youn, S.-W.; Lee, J.; Lee, H.-J.; Lee, T.-K.; Park, J.; et al. Adipokine resistin is a key player to modulate monocytes, endothelial cells, and smooth muscle cells, leading to progression of atherosclerosis in rabbit carotid artery. *J. Am. Coll. Cardiol.* **2011**, *57*, 99–109.
353. Zuniga, M.C.; Raghuraman, G.; Zhou, W. Physiologic levels of resistin induce a shift from proliferation to apoptosis in macrophage and VSMC co-culture. *Surgery* **2018**, *163*, 906–911.
354. Park, H.K.; Ahima, R.S. Resistin in rodents and humans. *Diabetes Metab. J.* **2013**, *37*, 404–414.
355. Jamaluddin, M.S.; Weakley, S.M.; Yao, Q.; Chen, C. Resistin: Functional roles and therapeutic considerations for cardiovascular disease. *Br. J. Pharmacol.* **2012**, *165*, 622–632.
356. Calabro, P.; Samudio, I.; Willerson, J.T.; Yeh, E.T.H. Resistin promotes smooth muscle cell proliferation through activation of extracellular signal-regulated kinase 1/2 and phosphatidylinositol 3-kinase pathways. *Circulation* **2004**, *110*, 3335–3340.
357. Kang, S.-W.; Kim, J.-L.; Kwon, G.T.; Lee, Y.-J.; Park, J.H.Y.; Lim, S.S.; Kang, Y.-H. Sensitive fern (Onoclea sensibilis) extract suppresses proliferation and migration of vascular smooth muscle cells inflamed by neighboring macrophages. *Biol. Pharm. Bull.* **2011**, *34*, 1717–1723.

358. Hirai, H.; Satoh, H.; Kudoh, A.; Watanabe, T. Interaction between resistin and adiponectin in the proliferation of rat vascular smooth muscle cells. *Mol. Cell. Endocrinol.* **2013**, *366*, 108–116.
359. Park, H.K.; Kwak, M.K.; Kim, H.J.; Ahima, R.S. Linking resistin, inflammation, and cardiometabolic diseases. *Korean, J. Intern. Med.* **2017**, *32*, 239–247.
360. Zuniga, M.C.; Raghuraman, G.; Zhou, W. Physiologic levels of resistin induce a shift from proliferation to apoptosis in macrophage and VSMC co-culture. *Surgery* **2018**, *163*, 906–911.
361. Jung, H.S.; Park, K.-H.; Cho, Y.M.; Chung, S.S.; Cho, H.J.; Cho, S.Y.; Kim, S.J.; Kim, S.Y.; Lee, H.K.; Park, K.S. Resistin is secreted from macrophages in atheromas and promotes atherosclerosis. *Cardiovasc. Res.* **2006**, *69*, 76–85.
362. Ding, Q.; Chai, H.; Mahmood, N.; Tsao, J.; Mochly-Rosen, D.; Zhou, W. Matrix metalloproteinases modulated by protein kinase Cε mediate resistin-induced migration of human coronary artery smooth muscle cells. *J. Vasc. Surg.* **2011**, *53*, 1044–1051.
363. Lee, H.M.; Kim, H.J.; Won, K.-J.; Choi, W.S.; Lee, K.-Y.; Bae, Y.M.; Park, P.-J.; Park, T.-K.; Lee, Y.L.; Lee, C.-K.; et al. Contribution of soluble intercellular adhesion molecule-1 to the migration of vascular smooth muscle cells. *Eur. J. Pharmacol.* **2008**, *579*, 260–268.
364. Payan, D.G. Receptor-mediated mitogenic effects of substance P on cultured smooth muscle cells. *Biochem. Biophys. Res. Commun.* **1985**, *130*, 104–109.
365. Yang, C.-M.; Hsiao, L.-D.; Chien, C.-S.; Lin, C.-C.; Luo, S.-F.; Wang, C.-C. Substance P-induced activation of p42/44 mitogen-activated protein kinase associated with cell proliferation in human tracheal smooth muscle cells. *Cell. Signal.* **2002**, *14*, 913–923.
366. Yamauchi, H.; Motomura, N.; Chung, U.-I.; Sata, M.; Takai, D.; Saito, A.; Ono, M.; Takamoto, S. Growth-associated hyperphosphatemia in young recipients accelerates aortic allograft calcification in a rat model. *J. Thorac. Cardiovasc. Surg.* **2013**, *145*, 522–530.
367. Kawada, S.; Nagasawa, Y.; Kawabe, M.; Ohyama, H.; Kida, A.; Kato-Kogoe, N.; Nanami, M.; Hasuike, Y.; Kuragano, T.; Kishimoto, H.; et al. Iron-induced calcification in human aortic vascular smooth muscle cells through interleukin-24 (IL-24), with/without TNF-alpha. *Sci. Rep.* **2018**, *8*, 658.
368. Shioi, A.; Katagi, M.; Okuno, Y.; Mori, K.; Jono, S.; Koyama, H.; Nishizawa, Y. Induction of bone-type alkaline phosphatase in human vascular smooth muscle cells: Roles of tumor necrosis factor-alpha and oncostatin M derived from macrophages. *Circ. Res.* **2002**, *91*, 9–16.
369. Lee, H.-L.; Woo, K.M.; Ryoo, H.-M.; Baek, J.-H. Tumor necrosis factor-alpha increases alkaline phosphatase expression in vascular smooth muscle cells via MSX2 induction. *Biochem. Biophys. Res. Commun.* **2010**, *391*, 1087–1092.
370. Aghagolzadeh, P.; Bachtler, M.; Bijarnia, R.; Jackson, C.; Smith, E.R.; Odermatt, A.; Radpour, R.; Pasch, A. Calcification of vascular smooth muscle cells is induced by secondary calciprotein particles and enhanced by tumor necrosis factor-α. *Atherosclerosis* **2016**, *251*, 404–414.
371. Ye, Y.; Bian, W.; Fu, F.; Hu, J.; Liu, H. Selenoprotein S inhibits inflammation-induced vascular smooth muscle cell calcification. *J. Biol. Inorg. Chem. JBIC Publ. Soc. Biol. Inorg. Chem.* **2018**, *23*, 739–751.
372. Zickler, D.; Luecht, C.; Willy, K.; Chen, L.; Witowski, J.; Girndt, M.; Fiedler, R.; Storr, M.; Kamhieh-Milz, J.; Schoon, J.; et al. Tumour necrosis factor-alpha in uraemic serum promotes osteoblastic transition and calcification of vascular smooth muscle cells via extracellular signal-regulated kinases and activator protein 1/c-FOS-mediated induction of interleukin 6 expression. *Nephrol. Dial. Transplant. Off. Publ. Eur. Dial. Transpl. Assoc.—Eur. Ren. Assoc.* **2018**, *33*, 574–585.
373. García-Miguel, M.; Riquelme, J.A.; Norambuena-Soto, I.; Morales, P.E.; Sanhueza-Olivares, F.; Nuñez-Soto, C.; Mondaca-Ruff, D.; Cancino-Arenas, N.; San Martín, A.; Chiong, M. Autophagy mediates tumor necrosis factor-α-induced phenotype switching in vascular smooth muscle A7r5 cell line. *PLoS ONE* **2018**, *13*, e0197210.
374. Freise, C.; Querfeld, U. The lignan (+)-episesamin interferes with TNF-α-induced activation of VSMC via diminished activation of NF-κB, ERK1/2 and AKT and decreased activity of gelatinases. *Acta Physiol. Oxf. Engl.* **2015**, *213*, 642–652.
375. Chatterjee, A.; Sharma, A.; Chen, M.; Toy, R.; Mottola, G.; Conte, M.S. The pro-resolving lipid mediator maresin 1 (MaR1) attenuates inflammatory signaling pathways in vascular smooth muscle and endothelial cells. *PLoS ONE* **2014**, *9*, e113480.

376. Park, B.; Yim, J.-H.; Lee, H.-K.; Kim, B.-O.; Pyo, S. Ramalin inhibits VCAM-1 expression and adhesion of monocyte to vascular smooth muscle cells through MAPK and PADI4-dependent NF-kB and AP-1 pathways. *Biosci. Biotechnol. Biochem.* **2015**, *79*, 539–552.
377. Lee, Y.J.; Yoon, J.J.; Lee, S.M.; Kim, J.S.; Kang, D.G.; Lee, H.S. Inhibitory effect of Zanthoxylum schinifolium on vascular smooth muscle proliferation. *Immunopharmacol. Immunotoxicol.* **2012**, *34*, 354–361.
378. Cao, Q.; Jiang, Y.; Shi, J.; Xu, C.; Liu, X.; Yang, T.; Fu, P.; Niu, T. Artemisinin inhibits the proliferation, migration, and inflammatory reaction induced by tumor necrosis factor-α in vascular smooth muscle cells through nuclear factor kappa B pathway. *J. Surg. Res.* **2015**, *194*, 667–678.
379. Jiang, F.; Jiang, R.; Zhu, X.; Zhang, X.; Zhan, Z. Genipin inhibits TNF-α-induced vascular smooth muscle cell proliferation and migration via induction of HO-1. *PLoS ONE* **2013**, *8*, e74826.
380. Kazama, K.; Usui, T.; Okada, M.; Hara, Y.; Yamawaki, H. Omentin plays an anti-inflammatory role through inhibition of TNF-α-induced superoxide production in vascular smooth muscle cells. *Eur. J. Pharmacol.* **2012**, *686*, 116–123.
381. Choi, H.; Dikalova, A.; Stark, R.J.; Lamb, F.S. c-Jun N-terminal kinase attenuates TNFα signaling by reducing Nox1-dependent endosomal ROS production in vascular smooth muscle cells. *Free Radic. Biol. Med.* **2015**, *86*, 219–227.
382. Byeon, H.-E.; Park, B.-K.; Yim, J.H.; Lee, H.K.; Moon, E.-Y.; Rhee, D.-K.; Pyo, S. Stereocalpin A inhibits the expression of adhesion molecules in activated vascular smooth muscle cells. *Int. Immunopharmacol.* **2012**, *12*, 315–325.
383. Liu, J.; Zhang, L.; Ren, Y.; Gao, Y.; Kang, L.; Lu, S. Matrine inhibits the expression of adhesion molecules in activated vascular smooth muscle cells. *Mol. Med. Rep.* **2016**, *13*, 2313–2319.
384. Yan, X.; Wu, L.; Li, B.; Meng, X.; Dai, H.; Zheng, Y.; Fu, J. Cyanidin-3-O-glucoside Induces Apoptosis and Inhibits Migration of Tumor Necrosis Factor-α-Treated Rat Aortic Smooth Muscle Cells. *Cardiovasc. Toxicol.* **2016**, *16*, 251–259.
385. Yan, L.-J.; Yang, H.-T.; Duan, H.-Y.; Wu, J.-T.; Qian, P.; Fan, X.-W.; Wang, S. Myricitrin inhibits vascular adhesion molecule expression in TNF-α-stimulated vascular smooth muscle cells. *Mol. Med. Rep.* **2017**, *16*, 6354–6359.
386. Liu, S.; Lv, J.; Han, L.; Ichikawa, T.; Wang, W.; Li, S.; Wang, X.L.; Tang, D.; Cui, T. A pro-inflammatory role of deubiquitinating enzyme cylindromatosis (CYLD) in vascular smooth muscle cells. *Biochem. Biophys. Res. Commun.* **2012**, *420*, 78–83.
387. Ali, M.S.; Starke, R.M.; Jabbour, P.M.; Tjoumakaris, S.I.; Gonzalez, L.F.; Rosenwasser, R.H.; Owens, G.K.; Koch, W.J.; Greig, N.H.; Dumont, A.S. TNF-α induces phenotypic modulation in cerebral vascular smooth muscle cells: Implications for cerebral aneurysm pathology. *J. Cereb. Blood Flow Metab. Off. J. Int. Soc. Cereb. Blood Flow Metab.* **2013**, *33*, 1564–1573.
388. Park, S.H.; Koo, H.J.; Sung, Y.Y.; Kim, H.K. The protective effect of Prunella vulgaris ethanol extract against vascular inflammation in TNF-α-stimulated human aortic smooth muscle cells. *BMB Rep.* **2013**, *46*, 352–357.
389. Chen, S.; Ding, Y.; Tao, W.; Zhang, W.; Liang, T.; Liu, C. Naringenin inhibits TNF-α induced VSMC proliferation and migration via induction of HO-1. *Food Chem. Toxicol. Int. J. Publ. Br. Ind. Biol. Res. Assoc.* **2012**, *50*, 3025–3031.
390. Butoi, E.D.; Gan, A.M.; Manduteanu, I.; Stan, D.; Calin, M.; Pirvulescu, M.; Koenen, R.R.; Weber, C.; Simionescu, M. Cross talk between smooth muscle cells and monocytes/activated monocytes via CX3CL1/CX3CR1 axis augments expression of pro-atherogenic molecules. *Biochim. Biophys. Acta* **2011**, *1813*, 2026–2035.
391. Shu, Y.-N.; Dong, L.-H.; Li, H.; Pei, Q.-Q.; Miao, S.-B.; Zhang, F.; Zhang, D.-D.; Chen, R.; Yin, Y.-J.; Lin, Y.-L.; et al. CKII-SIRT1-SM22α loop evokes a self-limited inflammatory response in vascular smooth muscle cells. *Cardiovasc. Res.* **2017**, *113*, 1198–1207.
392. Tang, M.; Fang, J. TNF-α regulates apoptosis of human vascular smooth muscle cells through gap junctions. *Mol. Med. Rep.* **2017**, *15*, 1407–1411.
393. Cao, Q.; Jiang, Y.; Shi, J.; Liu, X.; Chen, J.; Niu, T.; Li, X. Artemisinin inhibits tumour necrosis factor-α-induced vascular smooth muscle cell proliferation in vitro and attenuates balloon injury-induced neointima formation in rats. *Clin. Exp. Pharmacol. Physiol.* **2015**, *42*, 502–509.
394. Rho, M.-C.; Ah Lee, K.; Mi Kim, S.; Sik Lee, C.; Jeong Jang, M.; Kook Kim, Y.; Sun Lee, H.; Hyun Choi, Y.; Yong Rhim, B.; Kim, K. Sensitization of vascular smooth muscle cell to TNF-alpha-mediated death in the presence of palmitate. *Toxicol. Appl. Pharmacol.* **2007**, *220*, 311–319.

395. Boyle, J.J.; Weissberg, P.L.; Bennett, M.R. Tumor necrosis factor-alpha promotes macrophage-induced vascular smooth muscle cell apoptosis by direct and autocrine mechanisms. *Arterioscler. Thromb. Vasc. Biol.* **2003**, *23*, 1553–1558.
396. Zhang, T.; Yang, Z.; Bo, X.; Wu, X.; Li, Z.; Guo, S. [Cathepsin B promoted the apoptosis of vascular smooth muscle cells as induced by tumor necrosis factor-α]. *Zhonghua Yi Xue Za Zhi* **2011**, *91*, 845–849.
397. Lee, H.S.; Chang, J.S.; Baek, J.A.; Chung, M.Y.; Lee, H.C.; Rhim, B.Y.; Sok, D.E.; Rho, M.-C.; Kim, Y.K.; Kim, K. TNF-alpha activates death pathway in human aorta smooth muscle cell in the presence of 7-ketocholesterol. *Biochem. Biophys. Res. Commun.* **2005**, *333*, 1093–1099.
398. Wang, Z.; Rao, P.J.; Castresana, M.R.; Newman, W.H. TNF-alpha induces proliferation or apoptosis in human saphenous vein smooth muscle cells depending on phenotype. *Am. J. Physiol. Heart Circ. Physiol.* **2005**, *288*, H293–H301.
399. Hans, C.P.; Zerfaoui, M.; Naura, A.S.; Catling, A.; Boulares, A.H. Differential effects of PARP inhibition on vascular cell survival and ACAT-1 expression favouring atherosclerotic plaque stability. *Cardiovasc. Res.* **2008**, *78*, 429–439.
400. Zhan, M.; Yuan, F.; Liu, H.; Chen, H.; Qiu, X.; Fang, W. Inhibition of proliferation and apoptosis of vascular smooth muscle cells by ghrelin. *Acta Biochim. Biophys. Sin.* **2008**, *40*, 769–776.
401. Tang, V.; Dhirapong, A.; Yabes, A.P.; Weiss, R.H. TNF-alpha-mediated apoptosis in vascular smooth muscle cells requires p73. *Am. J. Physiol. Cell Physiol.* **2005**, *289*, C199–C206.
402. Giordano, A.; Romano, S.; Nappo, G.; Messina, S.; Polimeno, M.; Corcione, N.; Cali, G.; Ferraro, P.; Monaco, M.; Zambrano, N.; et al. Atorvastatin sensitises vascular smooth muscle cells, but not endothelial cells, to TNF-α-induced cell death. *Curr. Pharm. Des.* **2012**, *18*, 6331–6338.
403. Jin, Y.-H.; Kim, S.-A. 2-Methoxycinnamaldehyde inhibits the TNF-α-induced proliferation and migration of human aortic smooth muscle cells. *Int. J. Mol. Med.* **2017**, *39*, 191–198.
404. Li, H.; Cheng, Y.; Simoncini, T.; Xu, S. 17β-Estradiol inhibits TNF-α-induced proliferation and migration of vascular smooth muscle cells via suppression of TRAIL. *Gynecol. Endocrinol. Off. J. Int. Soc. Gynecol. Endocrinol.* **2016**, *32*, 581–586.
405. Heo, S.-K.; Yun, H.-J.; Park, W.-H.; Park, S.-D. Rhein inhibits TNF-alpha-induced human aortic smooth muscle cell proliferation via mitochondrial-dependent apoptosis. *J. Vasc. Res.* **2009**, *46*, 375–386.
406. Qi, L.; Zhi, J.; Zhang, T.; Cao, X.; Sun, L.; Xu, Y.; Li, X. Inhibition of microRNA-25 by tumor necrosis factor α is critical in the modulation of vascular smooth muscle cell proliferation. *Mol. Med. Rep.* **2015**, *11*, 4353–4358.
407. Kang, Y.H.; Yang, I.J.; Shin, H.M. Herbal formula HMC05 prevents human aortic smooth muscle cell migration and proliferation by inhibiting the ERK1/2 MAPK signaling cascade. *J. Nat. Med.* **2012**, *66*, 177–184.
408. Lee, E.-J.; Kim, D.-I.; Kim, W.-J.; Moon, S.-K. Naringin inhibits matrix metalloproteinase-9 expression and AKT phosphorylation in tumor necrosis factor-alpha-induced vascular smooth muscle cells. *Mol. Nutr. Food Res.* **2009**, *53*, 1582–1591.
409. Li, H.; Liang, J.; Castrillon, D.H.; DePinho, R.A.; Olson, E.N.; Liu, Z.-P. FoxO4 regulates tumor necrosis factor alpha-directed smooth muscle cell migration by activating matrix metalloproteinase 9 gene transcription. *Mol. Cell. Biol.* **2007**, *27*, 2676–2686.
410. Rajesh, M.; Mukhopadhyay, P.; Haskó, G.; Huffman, J.W.; Mackie, K.; Pacher, P. CB2 cannabinoid receptor agonists attenuate TNF-alpha-induced human vascular smooth muscle cell proliferation and migration. *Br. J. Pharmacol.* **2008**, *153*, 347–357.
411. Coll-Bonfill, N.; Peinado, V.I.; Pisano, M.V.; Párrizas, M.; Blanco, I.; Evers, M.; Engelmann, J.C.; García-Lucio, J.; Tura-Ceide, O.; Meister, G.; et al. Slug Is Increased in Vascular Remodeling and Induces a Smooth Muscle Cell Proliferative Phenotype. *PLoS ONE* **2016**, *11*, e0159460.
412. Mattana, J.; Effiong, C.; Kapasi, A.; Singhal, P.C. Leukocyte-polytetrafluoroethylene interaction enhances proliferation of vascular smooth muscle cells via tumor necrosis factor-alpha secretion. *Kidney Int.* **1997**, *52*, 1478–1485.
413. Fan, S.; Li, X.; Lin, J.; Chen, S.; Shan, J.; Qi, G. Honokiol inhibits tumor necrosis factor-α-stimulated rat aortic smooth muscle cell proliferation via caspase- and mitochondrial-dependent apoptosis. *Inflammation* **2014**, *37*, 17–26.

414. Suh, S.-J.; Kwak, C.-H.; Chung, T.-W.; Park, S.J.; Cheeeei, M.; Park, S.-S.; Seo, C.-S.; Son, J.-K.; Chang, Y.-C.; Park, Y.-G.; et al. Pimaric acid from Aralia cordata has an inhibitory effect on TNF-α-induced MMP-9 production and HASMC migration via down-regulated NF-κB and AP-1. *Chem. Biol. Interact.* **2012**, *199*, 112–119.
415. Goetze, S.; Xi, X.P.; Kawano, Y.; Kawano, H.; Fleck, E.; Hsueh, W.A.; Law, R.E. TNF-alpha-induced migration of vascular smooth muscle cells is MAPK dependent. *Hypertens. Dallas Tex 1979* **1999**, *33*, 183–189.
416. Kim, J.; Ko, J. Human sLZIP promotes atherosclerosis via MMP-9 transcription and vascular smooth muscle cell migration. *FASEB J. Off. Publ. Fed. Am. Soc. Exp. Biol.* **2014**, *28*, 5010–5021.
417. Lee, S.-O.; Chang, Y.-C.; Whang, K.; Kim, C.-H.; Lee, I.-S. Role of NAD(P)H:quinone oxidoreductase 1 on tumor necrosis factor-alpha-induced migration of human vascular smooth muscle cells. *Cardiovasc. Res.* **2007**, *76*, 331–339.
418. Yu, Y.-M.; Lin, H.-C. Curcumin prevents human aortic smooth muscle cells migration by inhibiting of MMP-9 expression. *Nutr. Metab. Cardiovasc. Dis. NMCD* **2010**, *20*, 125–132.
419. Kaga, T.; Kawano, H.; Sakaguchi, M.; Nakazawa, T.; Taniyama, Y.; Morishita, R. Hepatocyte growth factor stimulated angiogenesis without inflammation: Differential actions between hepatocyte growth factor, vascular endothelial growth factor and basic fibroblast growth factor. *Vascul. Pharmacol.* **2012**, *57*, 3–9.
420. Xu, Y.-G.; Zhou, S.-H.; Li, Y.-G.; Zheng, C.-H.; Li, X.-P.; Liu, Q.-M.; Xu, D.-M.; Chen, S. The mechanism underlying vascular smooth muscle cell apoptosis induced by atorvastatin may be mainly associated with down-regulation of survivin expression. *Cardiovasc. Drugs Ther.* **2007**, *21*, 145–153.
421. Shi, X.; Guo, L.-W.; Seedial, S.M.; Si, Y.; Wang, B.; Takayama, T.; Suwanabol, P.A.; Ghosh, S.; DiRenzo, D.; Liu, B.; et al. TGF-β/Smad3 inhibit vascular smooth muscle cell apoptosis through an autocrine signaling mechanism involving VEGF-A. *Cell Death Dis.* **2014**, *5*, e1317.
422. Pei, Q.-M.; Jiang, P.; Yang, M.; Qian, X.-J.; Liu, J.-B.; Kim, S.-H. Roxithromycin inhibits VEGF-induced human airway smooth muscle cell proliferation: Opportunities for the treatment of asthma. *Exp. Cell Res.* **2016**, *347*, 378–384.
423. Liao, X.H.; Xiang, Y.; Li, H.; Zheng, D.L.; Xu, Y.; Xi Yu, C.; Li, J.P.; Zhang, X.Y.; Xing, W.B.; Cao, D.S.; et al. VEGF-A Stimulates STAT3 Activity via Nitrosylation of Myocardin to Regulate the Expression of Vascular Smooth Muscle Cell Differentiation Markers. *Sci. Rep.* **2017**, *7*, 2660.
424. Kim, S.-H.; Pei, Q.-M.; Jiang, P.; Yang, M.; Qian, X.-J.; Liu, J.-B. Effect of active vitamin D3 on VEGF-induced ADAM33 expression and proliferation in human airway smooth muscle cells: Implications for asthma treatment. *Respir. Res.* **2017**, *18*, 7.
425. Weatherford, D.A.; Sackman, J.E.; Reddick, T.T.; Freeman, M.B.; Stevens, S.L.; Goldman, M.H. Vascular endothelial growth factor and heparin in a biologic glue promotes human aortic endothelial cell proliferation with aortic smooth muscle cell inhibition. *Surgery* **1996**, *120*, 433–439.
426. Parenti, A.; Bellik, L.; Brogelli, L.; Filippi, S.; Ledda, F. Endogenous VEGF-A is responsible for mitogenic effects of MCP-1 on vascular smooth muscle cells. *Am. J. Physiol. Heart Circ. Physiol.* **2004**, *286*, H1978–H1984.
427. Kim, S.-H.; Pei, Q.-M.; Jiang, P.; Yang, M.; Qian, X.-J.; Liu, J.-B. Role of licochalcone A in VEGF-induced proliferation of human airway smooth muscle cells: Implications for asthma. *Growth Factors Chur Switz.* **2017**, *35*, 39–47.
428. Wan, J.; Lata, C.; Santilli, A.; Green, D.; Roy, S.; Santilli, S. Supplemental oxygen reverses hypoxia-induced smooth muscle cell proliferation by modulating HIF-alpha and VEGF levels in a rabbit arteriovenous fistula model. *Ann. Vasc. Surg.* **2014**, *28*, 725–736.
429. Schlich, R.; Willems, M.; Greulich, S.; Ruppe, F.; Knoefel, W.T.; Ouwens, D.M.; Maxhera, B.; Lichtenberg, A.; Eckel, J.; Sell, H. VEGF in the crosstalk between human adipocytes and smooth muscle cells: Depot-specific release from visceral and perivascular adipose tissue. *Mediators Inflamm.* **2013**, *2013*, 982458.
430. Liao, X.-H.; Wang, N.; Zhao, D.-W.; Zheng, D.-L.; Zheng, L.; Xing, W.-J.; Ma, W.-J.; Bao, L.-Y.; Dong, J.; Zhang, T.-C. STAT3 Protein Regulates Vascular Smooth Muscle Cell Phenotypic Switch by Interaction with Myocardin. *J. Biol. Chem.* **2015**, *290*, 19641–19652.
431. Li, Y.; McRobb, L.S.; Khachigian, L.M. Inhibition of intimal thickening after vascular injury with a cocktail of vascular endothelial growth factor and cyclic Arg-Gly-Asp peptide. *Int. J. Cardiol.* **2016**, *220*, 185–191.
432. Yang, G.Y.; Yao, J.S.; Huey, M.; Hashimoto, T.; Young, W.L. Participation of PI3K and ERK1/2 pathways are required for human brain vascular smooth muscle cell migration. *Neurochem. Int.* **2004**, *44*, 441–446.

433. Banerjee, S.; Mehta, S.; Haque, I.; Sengupta, K.; Dhar, K.; Kambhampati, S.; Van Veldhuizen, P.J.; Banerjee, S.K. VEGF-A165 induces human aortic smooth muscle cell migration by activating neuropilin-1-VEGFR1-PI3K axis. *Biochemistry* **2008**, *47*, 3345–3351.
434. Yao, J.S.; Chen, Y.; Zhai, W.; Xu, K.; Young, W.L.; Yang, G.-Y. Minocycline exerts multiple inhibitory effects on vascular endothelial growth factor-induced smooth muscle cell migration: The role of ERK1/2, PI3K, and matrix metalloproteinases. *Circ. Res.* **2004**, *95*, 364–371.
435. Grosskreutz, C.L.; Anand-Apte, B.; Dupláa, C.; Quinn, T.P.; Terman, B.I.; Zetter, B.; D'Amore, P.A. Vascular endothelial growth factor-induced migration of vascular smooth muscle cells in vitro. *Microvasc. Res.* **1999**, *58*, 128–136.
436. Wang, H.; Keiser, J.A. Vascular endothelial growth factor upregulates the expression of matrix metalloproteinases in vascular smooth muscle cells: Role of flt-1. *Circ. Res.* **1998**, *83*, 832–840.
437. Ho-Tin-Noé, B.; Le Dall, J.; Gomez, D.; Louedec, L.; Vranckx, R.; El-Bouchtaoui, M.; Legrès, L.; Meilhac, O.; Michel, J.-B. Early atheroma-derived agonists of peroxisome proliferator-activated receptor-γ trigger intramedial angiogenesis in a smooth muscle cell-dependent manner. *Circ. Res.* **2011**, *109*, 1003–1014.
438. Benitez, R.; Delgado-Maroto, V.; Caro, M.; Forte-Lago, I.; Duran-Prado, M.; O'Valle, F.; Lichtman, A.H.; Gonzalez-Rey, E.; Delgado, M. Vasoactive Intestinal Peptide Ameliorates Acute Myocarditis and Atherosclerosis by Regulating Inflammatory and Autoimmune Responses. *J. Immunol. Baltim. Md 1950* **2018**, *200*, 3697–3710.
439. Li, W.; Hamada, Y.; Nakashima, E.; Naruse, K.; Kamiya, H.; Akiyama, N.; Hirooka, H.; Takahashi, N.; Horiuchi, S.; Hotta, N.; et al. Suppression of 3-deoxyglucosone and heparin-binding epidermal growth factor-like growth factor mRNA expression by an aldose reductase inhibitor in rat vascular smooth muscle cells. *Biochem. Biophys. Res. Commun.* **2004**, *314*, 370–376.
440. Partridge, C.R.; Williams, E.S.; Barhoumi, R.; Tadesse, M.G.; Johnson, C.D.; Lu, K.P.; Meininger, G.A.; Wilson, E.; Ramos, K.S. Novel genomic targets in oxidant-induced vascular injury. *J. Mol. Cell. Cardiol.* **2005**, *38*, 983–996.
441. Newaz, M.; Yousefipour, Z. Acrolein-induced inflammatory signaling in vascular smooth muscle cells requires activation of serum response factor (SRF) and NFκB. *J. Basic Clin. Physiol. Pharmacol.* **2013**, *24*, 287–297.
442. Haldar, S.; Dru, C.; Choudhury, D.; Mishra, R.; Fernandez, A.; Biondi, S.; Liu, Z.; Shimada, K.; Arditi, M.; Bhowmick, N.A. Inflammation and pyroptosis mediate muscle expansion in an interleukin-1β (IL-1β)-dependent manner. *J. Biol. Chem.* **2015**, *290*, 6574–6583.
443. Chen, L.; Wu, X.; Zeb, F.; Huang, Y.; An, J.; Jiang, P.; Chen, A.; Xu, C.; Feng, Q. Acrolein-induced apoptosis of smooth muscle cells through NEAT1-Bmal1/Clock pathway and a protection from asparagus extract. *Environ. Pollut. Barking Essex 1987* **2020**, *258*, 113735.
444. Dong, Y.; Noda, K.; Murata, M.; Yoshida, S.; Saito, W.; Kanda, A.; Ishida, S. Localization of Acrolein-Lysine Adduct in Fibrovascular Tissues of Proliferative Diabetic Retinopathy. *Curr. Eye Res.* **2017**, *42*, 111–117.
445. Vindis, C.; Escargueil-Blanc, I.; Uchida, K.; Elbaz, M.; Salvayre, R.; Negre-Salvayre, A. Lipid oxidation products and oxidized low-density lipoproteins impair platelet-derived growth factor receptor activity in smooth muscle cells: Implication in atherosclerosis. *Redox Rep. Commun. Free Radic. Res.* **2007**, *12*, 96–100.
446. Van Campenhout, A.; Moran, C.S.; Parr, A.; Clancy, P.; Rush, C.; Jakubowski, H.; Golledge, J. Role of homocysteine in aortic calcification and osteogenic cell differentiation. *Atherosclerosis* **2009**, *202*, 557–566.
447. Liu, T.; Lin, J.; Ju, T.; Chu, L.; Zhang, L. Vascular smooth muscle cell differentiation to an osteogenic phenotype involves matrix metalloproteinase-2 modulation by homocysteine. *Mol. Cell. Biochem.* **2015**, *406*, 139–149.
448. Li, J.; Chai, S.; Tang, C.; Du, J. Homocysteine potentiates calcification of cultured rat aortic smooth muscle cells. *Life Sci.* **2003**, *74*, 451–461.
449. Ren, J.-L.; Hou, Y.-L.; Ni, X.-Q.; Zhu, Q.; Chen, Y.; Zhang, L.-S.; Liu, X.; Xue, C.-D.; Wu, N.; Yu, Y.-R.; et al. Intermedin1-53 Ameliorates Homocysteine-Promoted Atherosclerotic Calcification by Inhibiting Endoplasmic Reticulum Stress. *J. Cardiovasc. Pharmacol. Ther.* **2019**, 1074248419885633.
450. Zhu, L.; Zhang, N.; Yan, R.; Yang, W.; Cong, G.; Yan, N.; Ma, W.; Hou, J.; Yang, L.; Jia, S. Hyperhomocysteinemia induces vascular calcification by activating the transcription factor RUNX2 via Krüppel-like factor 4 up-regulation in mice. *J. Biol. Chem.* **2019**, *294*, 19465–19474.
451. Kim, B.J.; Kim, B.S.; Kang, J.H. Plasma homocysteine and coronary artery calcification in Korean men. *Eur. J. Prev. Cardiol.* **2015**, *22*, 478–485.

452. Peña-Duque, M.A.; Baños-González, M.A.; Valente-Acosta, B.; Rodríguez-Lobato, L.G.; Martínez-Ríos, M.A.; Cardoso-Saldaña, G.; Barragán-García, R.; Herrera-Alarcón, V.; Linares-López, C.; Delgado-Granados, H.; et al. Homocysteine is related to aortic mineralization in patients with ischemic heart disease. *J. Atheroscler. Thromb.* **2012**, *19*, 292–297.
453. Kronenberg, F.; Mündle, M.; Längle, M.; Neyer, U. Prevalence and progression of peripheral arterial calcifications in patients with ESRD. *Am. J. Kidney Dis. Off. J. Natl. Kidney Found.* **2003**, *41*, 140–148.
454. Rasouli, M.L.; Nasir, K.; Blumenthal, R.S.; Park, R.; Aziz, D.C.; Budoff, M.J. Plasma homocysteine predicts progression of atherosclerosis. *Atherosclerosis* **2005**, *181*, 159–165.
455. Kullo, I.J.; Li, G.; Bielak, L.F.; Bailey, K.R.; Sheedy, P.F.; Peyser, P.A.; Turner, S.T.; Kardia, S.L.R. Association of plasma homocysteine with coronary artery calcification in different categories of coronary heart disease risk. *Mayo Clin. Proc.* **2006**, *81*, 177–182.
456. Baber, U.; de Lemos, J.A.; Khera, A.; McGuire, D.K.; Omland, T.; Toto, R.D.; Hedayati, S.S. Non-traditional risk factors predict coronary calcification in chronic kidney disease in a population-based cohort. *Kidney Int.* **2008**, *73*, 615–621.
457. Held, C.; Sumner, G.; Sheridan, P.; McQueen, M.; Smith, S.; Dagenais, G.; Yusuf, S.; Lonn, E. Correlations between plasma homocysteine and folate concentrations and carotid atherosclerosis in high-risk individuals: Baseline data from the Homocysteine and Atherosclerosis Reduction Trial (HART). *Vasc. Med. Lond. Engl.* **2008**, *13*, 245–253.
458. Karger, A.B.; Steffen, B.T.; Nomura, S.O.; Guan, W.; Garg, P.K.; Szklo, M.; Budoff, M.J.; Tsai, M.Y. Association Between Homocysteine and Vascular Calcification Incidence, Prevalence, and Progression in the MESA Cohort. *J. Am. Heart Assoc.* **2020**, *9*, e013934.
459. Novaro, G.M.; Aronow, H.D.; Mayer-Sabik, E.; Griffin, B.P. Plasma homocysteine and calcific aortic valve disease. *Heart Br. Card. Soc.* **2004**, *90*, 802–803.
460. de Menezes, F.L.; Koch-Nogueira, P.C.; do Val, M.L.D.M.; Pestana, J.O.M.; Jorgetti, V.; Dos Reis, M.A.; Dos Reis Monteiro, M.L.G.; Leite, H.P. Is arterial calcification in children and adolescents with end-stage renal disease a rare finding? *Nephrol. Carlton Vic* **2019**, *24*, 696–702.
461. Godsland, I.F.; Elkeles, R.S.; Feher, M.D.; Nugara, F.; Rubens, M.B.; Richmond, W.; Khan, M.; Donovan, J.; Anyaoku, V.; Flather, M.D.; et al. Coronary calcification, homocysteine, C-reactive protein and the metabolic syndrome in Type 2 diabetes: The Prospective Evaluation of Diabetic Ischaemic Heart Disease by Coronary Tomography (PREDICT) Study. *Diabet. Med. J. Br. Diabet. Assoc.* **2006**, *23*, 1192–1200.
462. Krajnc, M.; Pečovnik Balon, B.; Krajnc, I. Non-traditional risk factors for coronary calcification and its progression in patients with type 2 diabetes: The impact of postprandial glycemia and fetuin-A. *J. Int. Med. Res.* **2019**, *47*, 846–858.
463. Nonaka, H.; Tsujino, T.; Watari, Y.; Emoto, N.; Yokoyama, M. Taurine prevents the decrease in expression and secretion of extracellular superoxide dismutase induced by homocysteine: Amelioration of homocysteine-induced endoplasmic reticulum stress by taurine. *Circulation* **2001**, *104*, 1165–1170.
464. Wang, G.; Siow, Y.L.; O, K. Homocysteine stimulates nuclear factor kappaB activity and monocyte chemoattractant protein-1 expression in vascular smooth-muscle cells: A possible role for protein kinase C. *Biochem. J.* **2000**, *352 Pt 3*, 817–826.
465. Ke, X.D.; Foucault-Bertaud, A.; Genovesio, C.; Dignat-George, F.; Lamy, E.; Charpiot, P. Homocysteine modulates the proteolytic potential of human arterial smooth muscle cells through a reactive oxygen species dependant mechanism. *Mol. Cell. Biochem.* **2010**, *335*, 203–210.
466. Papatheodorou, L.; Weiss, N. Vascular oxidant stress and inflammation in hyperhomocysteinemia. *Antioxid. Redox Signal.* **2007**, *9*, 1941–1958.
467. Su, J.; Wang, S.; Hunag, Y.; Jinag, Y. [A comparative study on pathogenic effects of homocysteine and cysteine on atherosclerosis]. *Wei Sheng Yan Jiu* **2009**, *38*, 43–46.
468. Wei, L.-H.; Chao, N.-X.; Gao, S.; Yu, Y.-T.; Shi, L.; Ma, X.-B.; Liao, N.; Lan, K.; Luo, Y.; Xie, Z.-Y.; et al. Homocysteine induces vascular inflammatory response via SMAD7 hypermethylation in human umbilical vein smooth muscle cells. *Microvasc. Res.* **2018**, *120*, 8–12.
469. Zhao, J.; Chen, H.; Liu, N.; Chen, J.; Gu, Y.; Chen, J.; Yang, K. Role of Hyperhomocysteinemia and Hyperuricemia in Pathogenesis of Atherosclerosis. *J. Stroke Cerebrovasc. Dis. Off. J. Natl. Stroke Assoc.* **2017**, *26*, 2695–2699.

470. Chang, L.; Xu, J.; Zhao, J.; Pang, Y.; Tang, C.; Qi, Y. Taurine antagonized oxidative stress injury induced by homocysteine in rat vascular smooth muscle cells. *Acta Pharmacol. Sin.* **2004**, *25*, 341–346.
471. Pang, X.; Si, J.; Xu, S.; Li, Y.; Liu, J. Simvastatin inhibits homocysteine-induced CRP generation via interfering with the ROS-p38/ERK1/2 signal pathway in rat vascular smooth muscle cells. *Vascul. Pharmacol.* **2017**, *88*, 42–47.
472. Desai, A.; Lankford, H.A.; Warren, J.S. Homocysteine augments cytokine-induced chemokine expression in human vascular smooth muscle cells: Implications for atherogenesis. *Inflammation* **2001**, *25*, 179–186.
473. Zhang, L.; Jin, M.; Hu, X.-S.; Zhu, J.-H. Homocysteine stimulates nuclear factor kappaB activity and interleukin-6 expression in rat vascular smooth muscle cells. *Cell Biol. Int.* **2006**, *30*, 592–597.
474. Pang, X.; Liu, J.; Zhao, J.; Mao, J.; Zhang, X.; Feng, L.; Han, C.; Li, M.; Wang, S.; Wu, D. Homocysteine induces the expression of C-reactive protein via NMDAr-ROS-MAPK-NF-κB signal pathway in rat vascular smooth muscle cells. *Atherosclerosis* **2014**, *236*, 73–81.
475. Huang, T.; Yuan, G.; Zhang, Z.; Zou, Z.; Li, D. Cardiovascular pathogenesis in hyperhomocysteinemia. *Asia Pac. J. Clin. Nutr.* **2008**, *17*, 8–16.
476. Buemi, M.; Marino, D.; Di Pasquale, G.; Floccari, F.; Ruello, A.; Aloisi, C.; Corica, F.; Senatore, M.; Romeo, A.; Frisina, N. Effects of homocysteine on proliferation, necrosis, and apoptosis of vascular smooth muscle cells in culture and influence of folic acid. *Thromb. Res.* **2001**, *104*, 207–213.
477. Rasmussen, L.M.; Hansen, P.R.; Ledet, T. Homocysteine and the production of collagens, proliferation and apoptosis in human arterial smooth muscle cells. *APMIS Acta Pathol. Microbiol. Immunol. Scand.* **2004**, *112*, 598–604.
478. Zhan, X.L.; Yang, X.H.; Gu, Y.H.; Guo, L.L.; Jin, H.M. Epigallocatechin gallate protects against homocysteine-induced vascular smooth muscle cell proliferation. *Mol. Cell. Biochem.* **2018**, *439*, 131–140.
479. Zhang, H.-P.; Wang, Y.-H.; Cao, C.-J.; Yang, X.-M.; Ma, S.-C.; Han, X.-B.; Yang, X.-L.; Yang, A.-N.; Tian, J.; Xu, H.; et al. A regulatory circuit involving miR-143 and DNMT3a mediates vascular smooth muscle cell proliferation induced by homocysteine. *Mol. Med. Rep.* **2016**, *13*, 483–490.
480. Ma, S.-C.; Zhang, H.-P.; Jiao, Y.; Wang, Y.-H.; Zhang, H.; Yang, X.-L.; Yang, A.-N.; Jiang, Y.-D. Homocysteine-induced proliferation of vascular smooth muscle cells occurs via PTEN hypermethylation and is mitigated by Resveratrol. *Mol. Med. Rep.* **2018**, *17*, 5312–5319.
481. Meng, L.; Zhou, C.; Guo, Y.; Zhai, X.; Jiang, C.; Li, G.; Chi, J.; Guo, H. [Exploring the active ingredient of Chinese yellow wine which could inhibit the Hcy induced proliferation and migration of vascular smooth muscle cells]. *Zhongguo Ying Yong Sheng Li Xue Za Zhi Zhongguo Yingyong Shenglixue Zazhi Chin. J. Appl. Physiol.* **2015**, *31*, 437–442.
482. Shirpoor, A.; Salami, S.; Khadem Ansari, M.-H.; Ilkhanizadeh, B.; Abdollahzadeh, N. Ethanol promotes rat aortic vascular smooth muscle cell proliferation via increase of homocysteine and oxidized-low-density lipoprotein. *J. Cardiol.* **2013**, *62*, 374–378.
483. Han, X.-B.; Zhang, H.-P.; Cao, C.-J.; Wang, Y.-H.; Tian, J.; Yang, X.-L.; Yang, A.-N.; Wang, J.; Jiang, Y.-D.; Xu, H. Aberrant DNA methylation of the PDGF gene in homocysteine-mediated VSMC proliferation and its underlying mechanism. *Mol. Med. Rep.* **2014**, *10*, 947–954.
484. Lin, H.; Ni, T.; Zhang, J.; Meng, L.; Gao, F.; Pan, S.; Luo, H.; Xu, F.; Ru, G.; Chi, J.; et al. Knockdown of Herp alleviates hyperhomocysteinemia mediated atherosclerosis through the inhibition of vascular smooth muscle cell phenotype switching. *Int. J. Cardiol.* **2018**, *269*, 242–249.
485. Lee, H.-Y.; Chae, I.-H.; Kim, H.-S.; Park, Y.-B.; Choi, Y.-S.; Lee, Y.-W.; Park, S.-J.; Cha, Y.-J. Differential effects of homocysteine on porcine endothelial and vascular smooth muscle cells. *J. Cardiovasc. Pharmacol.* **2002**, *39*, 643–651.
486. Tsai, J.C.; Perrella, M.A.; Yoshizumi, M.; Hsieh, C.M.; Haber, E.; Schlegel, R.; Lee, M.E. Promotion of vascular smooth muscle cell growth by homocysteine: A link to atherosclerosis. *Proc. Natl. Acad. Sci. U. S. A.* **1994**, *91*, 6369–6373.
487. Carmody, B.J.; Arora, S.; Avena, R.; Cosby, K.; Sidawy, A.N. Folic acid inhibits homocysteine-induced proliferation of human arterial smooth muscle cells. *J. Vasc. Surg.* **1999**, *30*, 1121–1128.
488. Yang, F.; Tan, H.-M.; Wang, H. Hyperhomocysteinemia and atherosclerosis. *Sheng Li Xue Bao* **2005**, *57*, 103–114.
489. Ma, S.-C.; Cao, J.-C.; Zhang, H.-P.; Jiao, Y.; Zhang, H.; He, Y.-Y.; Wang, Y.-H.; Yang, X.-L.; Yang, A.-N.; Tian, J.; et al. Aberrant promoter methylation of multiple genes in VSMC proliferation induced by Hcy. *Mol. Med. Rep.* **2017**, *16*, 7775–7783.

490. Bao, X.; Zheng, H. Atorvastatin attenuates homocysteine-induced migration of smooth muscle cells through mevalonate pathway involving reactive oxygen species and p38 MAPK. *Clin. Exp. Pharmacol. Physiol.* **2015**, *42*, 865–873.
491. Jiang, C.; Zhang, H.; Zhang, W.; Kong, W.; Zhu, Y.; Zhang, H.; Xu, Q.; Li, Y.; Wang, X. Homocysteine promotes vascular smooth muscle cell migration by induction of the adipokine resistin. *Am. J. Physiol. Cell Physiol.* **2009**, *297*, C1466–C1476.
492. Doronzo, G.; Russo, I.; Mattiello, L.; Trovati, M.; Anfossi, G. Homocysteine rapidly increases matrix metalloproteinase-2 expression and activity in cultured human vascular smooth muscle cells. Role of phosphatidyl inositol 3-kinase and mitogen activated protein kinase pathways. *Thromb. Haemost.* **2005**, *94*, 1285–1293.
493. Chen, Y.; Liu, R.; Zhang, G.; Yu, Q.; Jia, M.; Zheng, C.; Wang, Y.; Xu, C.; Zhang, Y.; Liu, E. Hypercysteinemia promotes atherosclerosis by reducing protein S-nitrosylation. *Biomed. Pharmacother. Biomedecine Pharmacother.* **2015**, *70*, 253–259.
494. Chen, J.; Zhang, X.; Zhang, H.; Liu, T.; Zhang, H.; Teng, J.; Ji, J.; Ding, X. Indoxyl Sulfate Enhance the Hypermethylation of Klotho and Promote the Process of Vascular Calcification in Chronic Kidney Disease. *Int. J. Biol. Sci.* **2016**, *12*, 1236–1246.
495. Hénaut, L.; Mary, A.; Chillon, J.-M.; Kamel, S.; Massy, Z. The Impact of Uremic Toxins on Vascular Smooth Muscle Cell Function. *Toxins* **2018**, *10*, 218.
496. Zhang, H.; Chen, J.; Shen, Z.; Gu, Y.; Xu, L.; Hu, J.; Zhang, X.; Ding, X. Indoxyl sulfate accelerates vascular smooth muscle cell calcification via microRNA-29b dependent regulation of Wnt/β-catenin signaling. *Toxicol. Lett.* **2018**, *284*, 29–36.
497. Wu, Y.; Han, X.; Wang, L.; Diao, Z.; Liu, W. Indoxyl sulfate promotes vascular smooth muscle cell calcification via the JNK/Pit-1 pathway. *Ren. Fail.* **2016**, *38*, 1702–1710.
498. Opdebeeck, B.; Maudsley, S.; Azmi, A.; De Maré, A.; De Leger, W.; Meijers, B.; Verhulst, A.; Evenepoel, P.; D'Haese, P.C.; Neven, E. Indoxyl Sulfate and p-Cresyl Sulfate Promote Vascular Calcification and Associate with Glucose Intolerance. *J. Am. Soc. Nephrol. JASN* **2019**, *30*, 751–766.
499. Asami, M.; Tanabe, K.; Ito, S.; Yoshida, E.; Aoki, J.; Tanimoto, S.; Horiuchi, Y.; Yoshida, M. Impact of Indoxyl Sulfate on Coronary Plaques in Patients on Hemodialysis. *Int. Heart. J.* **2018**, *59*, 489–496.
500. Barreto, F.C.; Barreto, D.V.; Liabeuf, S.; Meert, N.; Glorieux, G.; Temmar, M.; Choukroun, G.; Vanholder, R.; Massy, Z.A.; European Uremic Toxin Work Group (EUTox) Serum indoxyl sulfate is associated with vascular disease and mortality in chronic kidney disease patients. *Clin. J. Am. Soc. Nephrol. CJASN* **2009**, *4*, 1551–1558.
501. Taki, K.; Takayama, F.; Tsuruta, Y.; Niwa, T. Oxidative stress, advanced glycation end product, and coronary artery calcification in hemodialysis patients. *Kidney Int.* **2006**, *70*, 218–224.
502. Muteliefu, G.; Enomoto, A.; Jiang, P.; Takahashi, M.; Niwa, T. Indoxyl sulphate induces oxidative stress and the expression of osteoblast-specific proteins in vascular smooth muscle cells. *Nephrol. Dial. Transplant. Off. Publ. Eur. Dial. Transpl. Assoc.—Eur. Ren. Assoc.* **2009**, *24*, 2051–2058.
503. Shimizu, H.; Hirose, Y.; Goto, S.; Nishijima, F.; Zrelli, H.; Zghonda, N.; Niwa, T.; Miyazaki, H. Indoxyl sulfate enhances angiotensin II signaling through upregulation of epidermal growth factor receptor expression in vascular smooth muscle cells. *Life Sci.* **2012**, *91*, 172–177.
504. Shimizu, H.; Hirose, Y.; Nishijima, F.; Tsubakihara, Y.; Miyazaki, H. ROS and PDGF-beta [corrected] receptors are critically involved in indoxyl sulfate actions that promote vascular smooth muscle cell proliferation and migration. *Am. J. Physiol. Cell Physiol.* **2009**, *297*, C389–C396.
505. Yisireyili, M.; Saito, S.; Abudureyimu, S.; Adelibieke, Y.; Ng, H.-Y.; Nishijima, F.; Takeshita, K.; Murohara, T.; Niwa, T. Indoxyl sulfate-induced activation of (pro)renin receptor promotes cell proliferation and tissue factor expression in vascular smooth muscle cells. *PLoS ONE* **2014**, *9*, e109268.
506. Muteliefu, G.; Enomoto, A.; Niwa, T. Indoxyl sulfate promotes proliferation of human aortic smooth muscle cells by inducing oxidative stress. *J. Ren. Nutr. Off. J. Counc. Ren. Nutr. Natl. Kidney Found.* **2009**, *19*, 29–32.
507. Muteliefu, G.; Shimizu, H.; Enomoto, A.; Nishijima, F.; Takahashi, M.; Niwa, T. Indoxyl sulfate promotes vascular smooth muscle cell senescence with upregulation of p53, p21, and prelamin A through oxidative stress. *Am. J. Physiol. Cell Physiol.* **2012**, *303*, C126–C134.

508. Adelibieke, Y.; Yisireyili, M.; Ng, H.-Y.; Saito, S.; Nishijima, F.; Niwa, T. Indoxyl sulfate induces IL-6 expression in vascular endothelial and smooth muscle cells through OAT3-mediated uptake and activation of AhR/NF-κB pathway. *Nephron Exp. Nephrol.* **2014**, *128*, 1–8.
509. Yamamoto, H.; Tsuruoka, S.; Ioka, T.; Ando, H.; Ito, C.; Akimoto, T.; Fujimura, A.; Asano, Y.; Kusano, E. Indoxyl sulfate stimulates proliferation of rat vascular smooth muscle cells. *Kidney Int.* **2006**, *69*, 1780–1785.
510. Ryu, J.-H.; Park, H.; Kim, S.-J. The effects of indoxyl sulfate-induced endothelial microparticles on neointimal hyperplasia formation in an ex vivo model. *Ann. Surg. Treat. Res.* **2017**, *93*, 11–17.
511. Lin, C.-Y.; Hsu, S.-C.; Lee, H.-S.; Lin, S.-H.; Tsai, C.-S.; Huang, S.-M.; Shih, C.-C.; Hsu, Y.-J. Enhanced expression of glucose transporter-1 in vascular smooth muscle cells via the Akt/tuberous sclerosis complex subunit 2 (TSC2)/mammalian target of rapamycin (mTOR)/ribosomal S6 protein kinase (S6K) pathway in experimental renal failure. *J. Vasc. Surg.* **2013**, *57*, 475–485.
512. Ng, H.-Y.; Bolati, W.; Lee, C.-T.; Chien, Y.-S.; Yisireyili, M.; Saito, S.; Pei, S.-N.; Nishijima, F.; Niwa, T. Indoxyl Sulfate Downregulates Mas Receptor via Aryl Hydrocarbon Receptor/Nuclear Factor-kappa B, and Induces Cell Proliferation and Tissue Factor Expression in Vascular Smooth Muscle Cells. *Nephron* **2016**, *133*, 205–212.
513. Mozar, A.; Louvet, L.; Morlière, P.; Godin, C.; Boudot, C.; Kamel, S.; Drüeke, T.B.; Massy, Z.A. Uremic toxin indoxyl sulfate inhibits human vascular smooth muscle cell proliferation. *Ther. Apher. Dial. Off. Peer-Rev. J. Int. Soc. Apher. Jpn. Soc. Apher. Jpn. Soc. Dial. Ther.* **2011**, *15*, 135–139.
514. Taki, K.; Tsuruta, Y.; Niwa, T. Indoxyl sulfate and atherosclerotic risk factors in hemodialysis patients. *Am. J. Nephrol.* **2007**, *27*, 30–35.
515. Parhami, F.; Tintut, Y.; Ballard, A.; Fogelman, A.M.; Demer, L.L. Leptin enhances the calcification of vascular cells: Artery wall as a target of leptin. *Circ. Res.* **2001**, *88*, 954–960.
516. Dubey, L.; Hesong, Z. Role of leptin in atherogenesis. *Exp. Clin. Cardiol.* **2006**, *11*, 269–275.
517. Zeadin, M.G.; Butcher, M.K.; Shaughnessy, S.G.; Werstuck, G.H. Leptin promotes osteoblast differentiation and mineralization of primary cultures of vascular smooth muscle cells by inhibiting glycogen synthase kinase (GSK)-3β. *Biochem. Biophys. Res. Commun.* **2012**, *425*, 924–930.
518. Zeadin, M.; Butcher, M.; Werstuck, G.; Khan, M.; Yee, C.K.; Shaughnessy, S.G. Effect of leptin on vascular calcification in apolipoprotein E-deficient mice. *Arterioscler. Thromb. Vasc. Biol.* **2009**, *29*, 2069–2075.
519. Chen, N.X.; O'Neill, K.; Akl, N.K.; Moe, S.M. Adipocyte induced arterial calcification is prevented with sodium thiosulfate. *Biochem. Biophys. Res. Commun.* **2014**, *449*, 151–156.
520. Iribarren, C.; Husson, G.; Go, A.S.; Lo, J.C.; Fair, J.M.; Rubin, G.D.; Hlatky, M.A.; Fortmann, S.P. Plasma leptin levels and coronary artery calcification in older adults. *J. Clin. Endocrinol. Metab.* **2007**, *92*, 729–732.
521. Szulc, P.; Amri, E.Z.; Varennes, A.; Panaia-Ferrari, P.; Fontas, E.; Goudable, J.; Chapurlat, R.; Breuil, V. Positive Association of High Leptin Level and Abdominal Aortic Calcification in Men The Prospective MINOS Study. *Circ. J. Off. J. Jpn. Circ. Soc.* **2018**, *82*, 2954–2961.
522. de Oliveira, R.B.; Liabeuf, S.; Okazaki, H.; Lenglet, A.; Desjardins, L.; Lemke, H.-D.; Vanholder, R.; Choukroun, G.; Massy, Z.A.; European Uremic Toxin Work Group (EUTox) The clinical impact of plasma leptin levels in a cohort of chronic kidney disease patients. *Clin. Kidney, J.* **2013**, *6*, 63–70.
523. Liu, G.-Y.; Liang, Q.-H.; Cui, R.-R.; Liu, Y.; Wu, S.-S.; Shan, P.-F.; Yuan, L.-Q.; Liao, E.-Y. Leptin promotes the osteoblastic differentiation of vascular smooth muscle cells from female mice by increasing RANKL expression. *Endocrinology* **2014**, *155*, 558–567.
524. Li, L.; Mamputu, J.-C.; Wiernsperger, N.; Renier, G. Signaling pathways involved in human vascular smooth muscle cell proliferation and matrix metalloproteinase-2 expression induced by leptin: Inhibitory effect of metformin. *Diabetes* **2005**, *54*, 2227–2234.
525. Ghantous, C.M.; Azrak, Z.; Hanache, S.; Abou-Kheir, W.; Zeidan, A. Differential Role of Leptin and Adiponectin in Cardiovascular System. *Int. J. Endocrinol.* **2015**, *2015*, 534320.
526. Huang, F.; Xiong, X.; Wang, H.; You, S.; Zeng, H. Leptin-induced vascular smooth muscle cell proliferation via regulating cell cycle, activating ERK1/2 and NF-kappaB. *Acta Biochim. Biophys. Sin.* **2010**, *42*, 325–331.
527. Shi, R.; Chen, X.; Zhu, J.; Chen, L.; Zhu, S. [Leptin promotes the proliferation of airway smooth muscle cells and the expressions of HIF-1α and NF-κB of hypoxic rats]. *Xi Bao Yu Fen Zi Mian Yi Xue Za Zhi Chin. J. Cell. Mol. Immunol.* **2015**, *31*, 32–35.

528. Yu, Y.-M.; Tsai, C.-C.; Tzeng, Y.-W.; Chang, W.-C.; Chiang, S.-Y.; Lee, M.-F. Ursolic acid suppresses leptin-induced cell proliferation in rat vascular smooth muscle cells. *Can. J. Physiol. Pharmacol.* **2017**, *95*, 811–818.
529. Liu, W.; Zhu, S.; Chen, Y.; Wu, X.; Ni, W.; Chen, Y.; Zhao, L. [The effects of leptin on apoptosis of airway smooth muscle cells via the PI3K/Akt signaling pathway]. *Zhonghua Jie He He Hu Xi Za Zhi Zhonghua Jiehe He Huxi Zazhi Chin. J. Tuberc. Respir. Dis.* **2012**, *35*, 915–918.
530. Schroeter, M.R.; Leifheit-Nestler, M.; Hubert, A.; Schumann, B.; Glückermann, R.; Eschholz, N.; Krüger, N.; Lutz, S.; Hasenfuss, G.; Konstantinides, S.; et al. Leptin promotes neointima formation and smooth muscle cell proliferation via NADPH oxidase activation and signalling in caveolin-rich microdomains. *Cardiovasc. Res.* **2013**, *99*, 555–565.
531. Schäfer, K.; Halle, M.; Goeschen, C.; Dellas, C.; Pynn, M.; Loskutoff, D.J.; Konstantinides, S. Leptin promotes vascular remodeling and neointimal growth in mice. *Arterioscler. Thromb. Vasc. Biol.* **2004**, *24*, 112–117.
532. Bodary, P.F.; Shen, Y.; Ohman, M.; Bahrou, K.L.; Vargas, F.B.; Cudney, S.S.; Wickenheiser, K.J.; Myers, M.G.; Eitzman, D.T. Leptin regulates neointima formation after arterial injury through mechanisms independent of blood pressure and the leptin receptor/STAT3 signaling pathways involved in energy balance. *Arterioscler. Thromb. Vasc. Biol.* **2007**, *27*, 70–76.
533. Oda, A.; Taniguchi, T.; Yokoyama, M. Leptin stimulates rat aortic smooth muscle cell proliferation and migration. *Kobe, J. Med. Sci.* **2001**, *47*, 141–150.
534. Chai, S.; Wang, W.; Liu, J.; Guo, H.; Zhang, Z.; Wang, C.; Wang, J. Leptin knockout attenuates hypoxia-induced pulmonary arterial hypertension by inhibiting proliferation of pulmonary arterial smooth muscle cells. *Transl. Res. J. Lab. Clin. Med.* **2015**, *166*, 772–782.
535. Xie, X.; Li, S.; Zhu, Y.; Liu, L.; Ke, R.; Wang, J.; Yan, X.; Yang, L.; Gao, L.; Zang, W.; et al. Egr-1 mediates leptin-induced PPARγ reduction and proliferation of pulmonary artery smooth muscle cells. *Mol. Biol. Cell* **2018**, *29*, 356–362.
536. Noblet, J.N.; Goodwill, A.G.; Sassoon, D.J.; Kiel, A.M.; Tune, J.D. Leptin augments coronary vasoconstriction and smooth muscle proliferation via a Rho-kinase-dependent pathway. *Basic Res. Cardiol.* **2016**, *111*, 25.
537. Shin, H.-J.; Oh, J.; Kang, S.M.; Lee, J.H.; Shin, M.-J.; Hwang, K.-C.; Jang, Y.; Chung, J.H. Leptin induces hypertrophy via p38 mitogen-activated protein kinase in rat vascular smooth muscle cells. *Biochem. Biophys. Res. Commun.* **2005**, *329*, 18–24.
538. Shen, Y.; He, Z.; Lu, D.; Ou, B.; Pan, J.; Wang, X.; Li, J. [Leptin promotes neointimal formation by stimulating vascular smooth muscle cell proliferation through leptin receptor]. *Zhonghua Xin Xue Guan Bing Za Zhi* **2009**, *37*, 634–638.
539. Schroeter, M.R.; Eschholz, N.; Herzberg, S.; Jerchel, I.; Leifheit-Nestler, M.; Czepluch, F.S.; Chalikias, G.; Konstantinides, S.; Schäfer, K. Leptin-dependent and leptin-independent paracrine effects of perivascular adipose tissue on neointima formation. *Arterioscler. Thromb. Vasc. Biol.* **2013**, *33*, 980–987.
540. Tsai, Y.-C.; Leu, S.-Y.; Peng, Y.-J.; Lee, Y.-M.; Hsu, C.-H.; Chou, S.-C.; Yen, M.-H.; Cheng, P.-Y. Genistein suppresses leptin-induced proliferation and migration of vascular smooth muscle cells and neointima formation. *J. Cell. Mol. Med.* **2017**, *21*, 422–431.
541. Tsai, Y.-C.; Lee, Y.-M.; Hsu, C.-H.; Leu, S.-Y.; Chiang, H.-Y.; Yen, M.-H.; Cheng, P.-Y. The effect of ferulic acid ethyl ester on leptin-induced proliferation and migration of aortic smooth muscle cells. *Exp. Mol. Med.* **2015**, *47*, e180.
542. Nair, P.; Radford, K.; Fanat, A.; Janssen, L.J.; Peters-Golden, M.; Cox, P.G. The effects of leptin on airway smooth muscle responses. *Am. J. Respir. Cell Mol. Biol.* **2008**, *39*, 475–481.
543. Rodríguez, A.; Gómez-Ambrosi, J.; Catalán, V.; Fortuño, A.; Frühbeck, G. Leptin inhibits the proliferation of vascular smooth muscle cells induced by angiotensin II through nitric oxide-dependent mechanisms. *Mediators Inflamm.* **2010**, *2010*, 105489.
544. Bohlen, F.; Kratzsch, J.; Mueller, M.; Seidel, B.; Friedman-Einat, M.; Witzigmann, H.; Teupser, D.; Koerner, A.; Storck, M.; Thiery, J. Leptin inhibits cell growth of human vascular smooth muscle cells. *Vascul. Pharmacol.* **2007**, *46*, 67–71.
545. Liu, R.; Chen, B.; Chen, J.; Lan, J. Leptin upregulates smooth muscle cell expression of MMP-9 to promote plaque destabilization by activating AP-1 via the leptin receptor/MAPK/ERK signaling pathways. *Exp. Ther. Med.* **2018**, *16*, 5327–5333.

546. Shan, J.; Nguyen, T.B.; Totary-Jain, H.; Dansky, H.; Marx, S.O.; Marks, A.R. Leptin-enhanced neointimal hyperplasia is reduced by mTOR and PI3K inhibitors. *Proc. Natl. Acad. Sci. U. S. A.* **2008**, *105*, 19006–19011.
547. Schneiderman, J.; Schaefer, K.; Kolodgie, F.D.; Savion, N.; Kotev-Emeth, S.; Dardik, R.; Simon, A.J.; Halak, M.; Pariente, C.; Engelberg, I.; et al. Leptin locally synthesized in carotid atherosclerotic plaques could be associated with lesion instability and cerebral emboli. *J. Am. Heart Assoc.* **2012**, *1*, e001727.
548. Beltowski, J. Leptin and atherosclerosis. *Atherosclerosis* **2006**, *189*, 47–60.
549. Rezzani, R.; Favero, G.; Stacchiotti, A.; Rodella, L.F. Endothelial and vascular smooth muscle cell dysfunction mediated by cyclophylin A and the atheroprotective effects of melatonin. *Life Sci.* **2013**, *92*, 875–882.
550. Pascua, P.; Camello-Almaraz, C.; Camello, P.J.; Martin-Cano, F.E.; Vara, E.; Fernandez-Tresguerres, J.A.; Pozo, M.J. Melatonin, and to a lesser extent growth hormone, restores colonic smooth muscle physiology in old rats. *J. Pineal Res.* **2011**, *51*, 405–415.
551. Sönmez, M.F.; Narin, F.; Balcioğlu, E. Melatonin and vitamin C attenuates alcohol-induced oxidative stress in aorta. *Basic Clin. Pharmacol. Toxicol.* **2009**, *105*, 410–415.
552. Shi, D.; Xiao, X.; Wang, J.; Liu, L.; Chen, W.; Fu, L.; Xie, F.; Huang, W.; Deng, W. Melatonin suppresses proinflammatory mediators in lipopolysaccharide-stimulated CRL1999 cells via targeting MAPK, NF-κB, c/EBPβ, and p300 signaling. *J. Pineal Res.* **2012**, *53*, 154–165.
553. Jin, H.; Wang, Y.; Zhou, L.; Liu, L.; Zhang, P.; Deng, W.; Yuan, Y. Melatonin attenuates hypoxic pulmonary hypertension by inhibiting the inflammation and the proliferation of pulmonary arterial smooth muscle cells. *J. Pineal Res.* **2014**, *57*, 442–450.
554. Chang, T.; Wang, R.; Wu, L. Methylglyoxal-induced nitric oxide and peroxynitrite production in vascular smooth muscle cells. *Free Radic. Biol. Med.* **2005**, *38*, 286–293.
555. Dhar, I.; Dhar, A.; Wu, L.; Desai, K. Arginine attenuates methylglyoxal- and high glucose-induced endothelial dysfunction and oxidative stress by an endothelial nitric-oxide synthase-independent mechanism. *J. Pharmacol. Exp. Ther.* **2012**, *342*, 196–204.
556. Wu, L.; Juurlink, B.H.J. Increased methylglyoxal and oxidative stress in hypertensive rat vascular smooth muscle cells. *Hypertens. Dallas Tex 1979* **2002**, *39*, 809–814.
557. Wang, H.; Liu, J.; Wu, L. Methylglyoxal-induced mitochondrial dysfunction in vascular smooth muscle cells. *Biochem. Pharmacol.* **2009**, *77*, 1709–1716.
558. Yabe-Nishimura, C.; Nishinaka, T.; Iwata, K.; Seo, H.G. Up-regulation of aldose reductase by the substrate, methylglyoxal. *Chem. Biol. Interact.* **2003**, *143–144*, 317–323.
559. Wu, L. The pro-oxidant role of methylglyoxal in mesenteric artery smooth muscle cells. *Can. J. Physiol. Pharmacol.* **2005**, *83*, 63–68.
560. Chang, K.C.; Paek, K.S.; Kim, H.J.; Lee, Y.S.; Yabe-Nishimura, C.; Seo, H.G. Substrate-Induced Up-Regulation of Aldose Reductase by Methylglyoxal, a Reactive Oxoaldehyde Elevated in Diabetes. *Mol. Pharmacol.* **2002**, *61*, 1184–1191.
561. Alomar, F.; Singh, J.; Jang, H.-S.; Rozanzki, G.J.; Shao, C.H.; Padanilam, B.J.; Mayhan, W.G.; Bidasee, K.R. Smooth muscle-generated methylglyoxal impairs endothelial cell-mediated vasodilatation of cerebral microvessels in type 1 diabetic rats. *Br. J. Pharmacol.* **2016**, *173*, 3307–3326.
562. Chang, T.; Wang, R.; Olson, D.J.H.; Mousseau, D.D.; Ross, A.R.S.; Wu, L. Modification of Akt1 by methylglyoxal promotes the proliferation of vascular smooth muscle cells. *FASEB J. Off. Publ. Fed. Am. Soc. Exp. Biol.* **2011**, *25*, 1746–1757.
563. Cao, W.; Chang, T.; Li, X.-Q.; Wang, R.; Wu, L. Dual effects of fructose on ChREBP and FoxO1/3α are responsible for AldoB up-regulation and vascular remodelling. *Clin. Sci. Lond. Engl. 1979* **2017**, *131*, 309–325.
564. Wang, Z.; Jiang, Y.; Liu, N.; Ren, L.; Zhu, Y.; An, Y.; Chen, D. Advanced glycation end-product Nε-carboxymethyl-Lysine accelerates progression of atherosclerotic calcification in diabetes. *Atherosclerosis* **2012**, *221*, 387–396.
565. Li, L.H.; Ye, F.; Fu, X.L.; Xu, S.N.; Bao, Z.Y.; Sun, Z.; Yan, J.C.; Wu, J.N.; Wang, Z.Q. [Association between serum Nε-carboxymethyl-lysine level and anterior tibial arterial plaque calcification in patients with diabetic foot post foot amputation]. *Zhonghua Xin Xue Guan Bing Za Zhi* **2017**, *45*, 958–962.
566. Kislinger, T.; Fu, C.; Huber, B.; Qu, W.; Taguchi, A.; Du Yan, S.; Hofmann, M.; Yan, S.F.; Pischetsrieder, M.; Stern, D.; et al. N(epsilon)-(carboxymethyl)lysine adducts of proteins are ligands for receptor for advanced glycation end products that activate cell signaling pathways and modulate gene expression. *J. Biol. Chem.* **1999**, *274*, 31740–31749.

567. Meloche, J.; Paulin, R.; Courboulin, A.; Lambert, C.; Barrier, M.; Bonnet, P.; Bisserier, M.; Roy, M.; Sussman, M.A.; Agharazii, M.; et al. RAGE-Dependent Activation of the Oncoprotein Pim1 Plays a Critical Role in Systemic Vascular Remodeling Processes. *Arterioscler. Thromb. Vasc. Biol.* **2011**, *31*, 2114–2124.
568. Maciel, R.A.P.; Rempel, L.C.T.; Bosquetti, B.; Finco, A.B.; Pecoits-Filho, R.; Souza, W.M. de; Stinghen, A.E.M. p-cresol but not p-cresyl sulfate stimulate MCP-1 production via NF-κB p65 in human vascular smooth muscle cells. *J. Bras. Nefrol. Orgao Of. Soc. Bras. E Lat.-Am. Nefrol.* **2016**, *38*, 153–160.
569. Guo, J.; Lu, L.; Hua, Y.; Huang, K.; Wang, I.; Huang, L.; Fu, Q.; Chen, A.; Chan, P.; Fan, H.; et al. Vasculopathy in the setting of cardiorenal syndrome: Roles of protein-bound uremic toxins. *Am. J. Physiol. Heart Circ. Physiol.* **2017**, *313*, H1–H13.
570. Liabeuf, S.; Barreto, D.V.; Barreto, F.C.; Meert, N.; Glorieux, G.; Schepers, E.; Temmar, M.; Choukroun, G.; Vanholder, R.; Massy, Z.A.; et al. Free p-cresylsulphate is a predictor of mortality in patients at different stages of chronic kidney disease. *Nephrol. Dial. Transplant. Off. Publ. Eur. Dial. Transpl. Assoc.—Eur. Ren. Assoc.* **2010**, *25*, 1183–1191.
571. Watanabe, H.; Miyamoto, Y.; Enoki, Y.; Ishima, Y.; Kadowaki, D.; Kotani, S.; Nakajima, M.; Tanaka, M.; Matsushita, K.; Mori, Y.; et al. p-Cresyl sulfate, a uremic toxin, causes vascular endothelial and smooth muscle cell damages by inducing oxidative stress. *Pharmacol. Res. Perspect.* **2015**, *3*, e00092.
572. Gross, P.; Massy, Z.A.; Henaut, L.; Boudot, C.; Cagnard, J.; March, C.; Kamel, S.; Drueke, T.B.; Six, I. Para-cresyl sulfate acutely impairs vascular reactivity and induces vascular remodeling. *J. Cell. Physiol.* **2015**, *230*, 2927–2935.
573. Han, H.; Chen, Y.; Zhu, Z.; Su, X.; Ni, J.; Du, R.; Zhang, R.; Jin, W. p-Cresyl sulfate promotes the formation of atherosclerotic lesions and induces plaque instability by targeting vascular smooth muscle cells. *Front. Med.* **2016**, *10*, 320–329.
574. Schmidt, S.; Westhoff, T.H.; Krauser, P.; Zidek, W.; van der Giet, M. The uraemic toxin phenylacetic acid increases the formation of reactive oxygen species in vascular smooth muscle cells. *Nephrol. Dial. Transplant. Off. Publ. Eur. Dial. Transpl. Assoc.—Eur. Ren. Assoc.* **2008**, *23*, 65–71.
575. Facchiano, F.; D'Arcangelo, D.; Riccomi, A.; Lentini, A.; Beninati, S.; Capogrossi, M.C. Transglutaminase activity is involved in polyamine-induced programmed cell death. *Exp. Cell Res.* **2001**, *271*, 118–129.
576. Bagdade, J.D.; Subbaiah, P.V.; Bartos, D.; Bartos, F.; Campbell, R.A. Polyamines: An unrecognised cardiovascular risk factor in chronic dialysis? *Lancet Lond. Engl.* **1979**, *1*, 412–413.
577. Subbaiah, P.V.; Bagdade, J.D. Polyamines and atherosclerosis. Platelet releasate and other mitogens stimulate putrescine transport in arterial smooth muscle cells. *Atherosclerosis* **1982**, *44*, 49–60.
578. Mrhová, O.; Hladovec, J.; Urbanová, D.; Rossmann, P. The effect of polyamines on the endothelium and vascular wall metabolism in the rat. *Czech. Med.* **1991**, *14*, 97–105.
579. De Meyer Guido, R.Y.; Grootaert Mandy, O.J.; Michiels Cédéric, F.; Kurdi Ammar; Schrijvers Dorien, M.; Martinet Wim Autophagy in Vascular Disease. *Circ. Res.* **2015**, *116*, 468–479.
580. Grossi, M.; Persson, L.; Swärd, K.; Turczyńska, K.M.; Forte, A.; Hellstrand, P.; Nilsson, B.-O. Inhibition of polyamine formation antagonizes vascular smooth muscle cell proliferation and preserves the contractile phenotype. *Basic Clin. Pharmacol. Toxicol.* **2014**, *115*, 379–388.
581. Nishida, K.; Abiko, T.; Ishihara, M.; Tomikawa, M. Arterial injury-induced smooth muscle cell proliferation in rats is accompanied by increase in polyamine synthesis and level. *Atherosclerosis* **1990**, *83*, 119–125.
582. Liang, M.; Ekblad, E.; Hellstrand, P.; Nilsson, B.-O. Polyamine synthesis inhibition attenuates vascular smooth muscle cell migration. *J. Vasc. Res.* **2004**, *41*, 141–147.
583. Michiels, C.F.; Kurdi, A.; Timmermans, J.-P.; De Meyer, G.R.Y.; Martinet, W. Spermidine reduces lipid accumulation and necrotic core formation in atherosclerotic plaques via induction of autophagy. *Atherosclerosis* **2016**, *251*, 319–327.
584. Ciceri, P.; Volpi, E.; Brenna, I.; Elli, F.; Borghi, E.; Brancaccio, D.; Cozzolino, M. The combination of lanthanum chloride and the calcimimetic calindol delays the progression of vascular smooth muscle cells calcification. *Biochem. Biophys. Res. Commun.* **2012**, *418*, 770–773.
585. Sinha-Hikim, I.; Shen, R.; Paul Lee, W.-N.N.; Crum, A.; Vaziri, N.D.; Norris, K.C. Effects of a novel cystine-based glutathione precursor on oxidative stress in vascular smooth muscle cells. *Am. J. Physiol. Cell Physiol.* **2010**, *299*, C638–C642.

586. Sinha-Hikim, I.; Shen, R.; Kovacheva, E.; Crum, A.; Vaziri, N.D.; Norris, K.C. Inhibition of apoptotic signalling in spermine-treated vascular smooth muscle cells by a novel glutathione precursor. *Cell Biol. Int.* **2010**, *34*, 503–511.
587. Zhu, L.; Xiao, R.; Zhang, X.; Lang, Y.; Liu, F.; Yu, Z.; Zhang, J.; Su, Y.; Lu, Y.; Wang, T.; et al. Spermine on Endothelial Extracellular Vesicles Mediates Smoking-Induced Pulmonary Hypertension Partially Through Calcium-Sensing Receptor. *Arterioscler. Thromb. Vasc. Biol.* **2019**, *39*, 482–495.
588. Wei, C.; Li, H.-Z.; Wang, Y.-H.; Peng, X.; Shao, H.-J.; Li, H.-X.; Bai, S.-Z.; Lu, X.-X.; Wu, L.-Y.; Wang, R.; et al. Exogenous spermine inhibits the proliferation of human pulmonary artery smooth muscle cells caused by chemically-induced hypoxia via the suppression of the ERK1/2- and PI3K/AKT-associated pathways. *Int. J. Mol. Med.* **2016**, *37*, 39–46.
589. Li, W.; Zheng, T.; Altura, B.T.; Altura, B.M. Magnesium modulates contractile responses of rat aorta to thiocyanate: A possible relationship to smoking-induced atherosclerosis. *Toxicol. Appl. Pharmacol.* **1999**, *157*, 77–84.
590. Nagayama, K.; Morino, K.; Sekine, O.; Nakagawa, F.; Ishikado, A.; Iwasaki, H.; Okada, T.; Tawa, M.; Sato, D.; Imamura, T.; et al. Duality of n-3 Polyunsaturated Fatty Acids on Mcp-1 Expression in Vascular Smooth Muscle: A Potential Role of 4-Hydroxy Hexenal. *Nutrients* **2015**, *7*, 8112–8126.
591. Lee, S.J.; Seo, K.W.; Yun, M.R.; Bae, S.S.; Lee, W.S.; Hong, K.W.; Kim, C.D. 4-Hydroxynonenal enhances MMP-2 production in vascular smooth muscle cells via mitochondrial ROS-mediated activation of the Akt/NF-kappaB signaling pathways. *Free Radic. Biol. Med.* **2008**, *45*, 1487–1492.
592. Manea, A.; Manea, S.-A.; Todirita, A.; Albulescu, I.C.; Raicu, M.; Sasson, S.; Simionescu, M. High-glucose-increased expression and activation of NADPH oxidase in human vascular smooth muscle cells is mediated by 4-hydroxynonenal-activated PPARα and PPARβ/δ. *Cell Tissue Res.* **2015**, *361*, 593–604.
593. Nègre-Salvayre, A.; Garoby-Salom, S.; Swiader, A.; Rouahi, M.; Pucelle, M.; Salvayre, R. Proatherogenic effects of 4-hydroxynonenal. *Free Radic. Biol. Med.* **2017**, *111*, 127–139.
594. Ruef, J.; Moser, M.; Bode, C.; Kübler, W.; Runge, M.S. 4-hydroxynonenal induces apoptosis, NF-kappaB-activation and formation of 8-isoprostane in vascular smooth muscle cells. *Basic Res. Cardiol.* **2001**, *96*, 143–150.
595. Xu, T.; Liu, S.; Ma, T.; Jia, Z.; Zhang, Z.; Wang, A. Aldehyde dehydrogenase 2 protects against oxidative stress associated with pulmonary arterial hypertension. *Redox Biol.* **2017**, *11*, 286–296.
596. Kalyankrishna, S.; Parmentier, J.-H.; Malik, K.U. Arachidonic acid-derived oxidation products initiate apoptosis in vascular smooth muscle cells. *Prostaglandins Other Lipid Mediat.* **2002**, *70*, 13–29.
597. Lee, J.Y.; Jung, G.Y.; Heo, H.J.; Yun, M.R.; Park, J.Y.; Bae, S.S.; Hong, K.W.; Lee, W.S.; Kim, C.D. 4-Hydroxynonenal induces vascular smooth muscle cell apoptosis through mitochondrial generation of reactive oxygen species. *Toxicol. Lett.* **2006**, *166*, 212–221.
598. Akiba, S.; Kumazawa, S.; Yamaguchi, H.; Hontani, N.; Matsumoto, T.; Ikeda, T.; Oka, M.; Sato, T. Acceleration of matrix metalloproteinase-1 production and activation of platelet-derived growth factor receptor beta in human coronary smooth muscle cells by oxidized LDL and 4-hydroxynonenal. *Biochim. Biophys. Acta* **2006**, *1763*, 797–804.
599. Kakishita, H.; Hattori, Y. Vascular smooth muscle cell activation and growth by 4-hydroxynonenal. *Life Sci.* **2001**, *69*, 689–697.
600. Escargueil-Blanc, I.; Salvayre, R.; Vacaresse, N.; Jürgens, G.; Darblade, B.; Arnal, J.F.; Parthasarathy, S.; Nègre-Salvayre, A. Mildly oxidized LDL induces activation of platelet-derived growth factor beta-receptor pathway. *Circulation* **2001**, *104*, 1814–1821.
601. Watanabe, T.; Pakala, R.; Katagiri, T.; Benedict, C.R. Lipid peroxidation product 4-hydroxy-2-nonenal acts synergistically with serotonin in inducing vascular smooth muscle cell proliferation. *Atherosclerosis* **2001**, *155*, 37–44.
602. Watanabe, T.; Pakala, R.; Katagiri, T.; Benedict, C.R. Mildly oxidized low-density lipoprotein acts synergistically with angiotensin II in inducing vascular smooth muscle cell proliferation. *J. Hypertens.* **2001**, *19*, 1065–1073.
603. Lee, S.J.; Baek, S.E.; Jang, M.A.; Kim, C.D. Osteopontin plays a key role in vascular smooth muscle cell proliferation via EGFR-mediated activation of AP-1 and C/EBPβ pathways. *Pharmacol. Res.* **2016**, *108*, 1–8.
604. Huang, S.; Boda, B.; Vernaz, J.; Ferreira, E.; Wiszniewski, L.; Constant, S. Establishment and characterization of an in vitro human small airway model (SmallAir™). *Eur. J. Pharm. Biopharm. Off. J. Arbeitsgemeinschaft Pharm. Verfahrenstechnik EV* **2017**, *118*, 68–72.

605. Hattori, Y.; Hattori, S.; Kasai, K. 4-hydroxynonenal prevents NO production in vascular smooth muscle cells by inhibiting nuclear factor-kappaB-dependent transcriptional activation of inducible NO synthase. *Arterioscler. Thromb. Vasc. Biol.* **2001**, *21*, 1179–1183.
606. Huh, J.Y.; Son, D.J.; Lee, Y.; Lee, J.; Kim, B.; Lee, H.M.; Jo, H.; Choi, S.; Ha, H.; Chung, M.-H. 8-Hydroxy-2-deoxyguanosine prevents plaque formation and inhibits vascular smooth muscle cell activation through Rac1 inactivation. *Free Radic. Biol. Med.* **2012**, *53*, 109–121.
607. Schepers, E.; Glorieux, G.; Dou, L.; Cerini, C.; Gayrard, N.; Louvet, L.; Maugard, C.; Preus, P.; Rodriguez-Ortiz, M.; Argiles, A.; et al. Guanidino compounds as cause of cardiovascular damage in chronic kidney disease: An in vitro evaluation. *Blood Purif.* **2010**, *30*, 277–287.
608. Jang, J.J.; Berkheimer, S.B.; Merchant, M.; Krishnaswami, A. Asymmetric dimethylarginine and coronary artery calcium scores are increased in patients infected with human immunodeficiency virus. *Atherosclerosis* **2011**, *217*, 514–517.
609. Iribarren, C.; Husson, G.; Sydow, K.; Wang, B.-Y.; Sidney, S.; Cooke, J.P. Asymmetric dimethyl-arginine and coronary artery calcification in young adults entering middle age: The CARDIA Study. *Eur. J. Cardiovasc. Prev. Rehabil. Off. J. Eur. Soc. Cardiol. Work. Groups Epidemiol. Prev. Card. Rehabil. Exerc. Physiol.* **2007**, *14*, 222–229.
610. Kobayashi, S.; Oka, M.; Maesato, K.; Ikee, R.; Mano, T.; Hidekazu, M.; Ohtake, T. Coronary Artery Calcification, ADMA, and Insulin Resistance in CKD Patients. *Clin. J. Am. Soc. Nephrol.* **2008**, *3*, 1289–1295.
611. Kiani, A.N.; Mahoney, J.A.; Petri, M. Asymmetric dimethylarginine is a marker of poor prognosis and coronary calcium in systemic lupus erythematosus. *J. Rheumatol.* **2007**, *34*, 1502–1505.
612. Krzanowski, M.; Krzanowska, K.; Gajda, M.; Dumnicka, P.; Kopeć, G.; Guzik, B.; Woziwodzka, K.; Dziewierz, A.; Litwin, J.A.; Sułowicz, W. Asymmetric dimethylarginine as a useful risk marker of radial artery calcification in patients with advanced kidney disease. *Pol. Arch. Intern. Med.* **2018**, *128*, 157–165.
613. Coen, G.; Mantella, D.; Sardella, D.; Beraldi, M.P.; Ferrari, I.; Pierantozzi, A.; Lippi, B.; Di Giulio, S. Asymmetric dimethylarginine, vascular calcifications and parathyroid hormone serum levels in hemodialysis patients. *J. Nephrol.* **2009**, *22*, 616–622.
614. Yuan, Q.; Jiang, D.-J.; Chen, Q.-Q.; Wang, S.; Xin, H.-Y.; Deng, H.-W.; Li, Y.-J. Role of asymmetric dimethylarginine in homocysteine-induced apoptosis of vascular smooth muscle cells. *Biochem. Biophys. Res. Commun.* **2007**, *356*, 880–885.
615. Pekarova, M.; Koudelka, A.; Kolarova, H.; Ambrozova, G.; Klinke, A.; Cerna, A.; Kadlec, J.; Trundova, M.; Sindlerova Svihalkova, L.; Kuchta, R.; et al. Asymmetric dimethyl arginine induces pulmonary vascular dysfunction via activation of signal transducer and activator of transcription 3 and stabilization of hypoxia-inducible factor 1-alpha. *Vascul. Pharmacol.* **2015**, *73*, 138–148.
616. Li, X.-H.; Peng, J.; Tan, N.; Wu, W.-H.; Li, T.-T.; Shi, R.-Z.; Li, Y.-J. Involvement of asymmetric dimethylarginine and Rho kinase in the vascular remodeling in monocrotaline-induced pulmonary hypertension. *Vascul. Pharmacol.* **2010**, *53*, 223–229.
617. Zhou, Y.; Lan, X.; Guo, H.; Zhang, Y.; Ma, L.; Cao, J. Rho/ROCK signal cascade mediates asymmetric dimethylarginine-induced vascular smooth muscle cells migration and phenotype change. *BioMed Res. Int.* **2014**, *2014*, 683707.
618. Sun, L.; Zhang, T.; Yu, X.; Xin, W.; Lan, X.; Zhang, D.; Huang, C.; Du, G. Asymmetric dimethylarginine confers the communication between endothelial and smooth muscle cells and leads to VSMC migration through p38 and ERK1/2 signaling cascade. *FEBS Lett.* **2011**, *585*, 2727–2734.
619. Zou, T.; Liu, N.; Li, S.; Su, Y.; Man, Y.; Lu, D. [Vitamin E inhibits homocysteine-mediated smooth muscle cell proliferation]. *Nan Fang Yi Ke Da Xue Xue Bao* **2007**, *27*, 783–786.
620. Fleischhacker, E.; Esenabhalu, V.E.; Holzmann, S.; Skrabal, F.; Koidl, B.; Kostner, G.M.; Graier, W.F. In human hypercholesterolemia increased reactivity of vascular smooth muscle cells is due to altered subcellular Ca(2+) distribution. *Atherosclerosis* **2000**, *149*, 33–42.
621. Alzoghaibi, M.A.; Walsh, S.W.; Willey, A.; Fowler, A.A.; Graham, M.F. Linoleic acid, but not oleic acid, upregulates the production of interleukin-8 by human intestinal smooth muscle cells isolated from patients with Crohn's disease. *Clin. Nutr. Edinb. Scotl.* **2003**, *22*, 529–535.

622. Newman, W.H.; Zunzunegui, R.G.; Warejcka, D.J.; Dalton, M.L.; Castresana, M.R. A reactive oxygen-generating system activates nuclear factor-kappaB and releases tumor necrosis factor-alpha in coronary smooth muscle cells. *J. Surg. Res.* **1999**, *85*, 142–147.
623. Hassoun, P.M.; Shedd, A.L.; Lanzillo, J.J.; Thappa, V.; Landman, M.J.; Fanburg, B.L. Inhibition of pulmonary artery smooth muscle cell growth by hypoxanthine, xanthine, and uric acid. *Am. J. Respir. Cell Mol. Biol.* **1992**, *6*, 617–624.
624. Rao, G.N.; Corson, M.A.; Berk, B.C. Uric acid stimulates vascular smooth muscle cell proliferation by increasing platelet-derived growth factor A-chain expression. *J. Biol. Chem.* **1991**, *266*, 8604–8608.
625. Ramana, K.V.; Friedrich, B.; Srivastava, S.; Bhatnagar, A.; Srivastava, S.K. Activation of nuclear factor-kappaB by hyperglycemia in vascular smooth muscle cells is regulated by aldose reductase. *Diabetes* **2004**, *53*, 2910–2920.
626. Hattori, Y.; Hattori, S.; Sato, N.; Kasai, K. High-glucose-induced nuclear factor kappaB activation in vascular smooth muscle cells. *Cardiovasc. Res.* **2000**, *46*, 188–197.
627. Reddy, A.B.M.; Ramana, K.V.; Srivastava, S.; Bhatnagar, A.; Srivastava, S.K. Aldose reductase regulates high glucose-induced ectodomain shedding of tumor necrosis factor (TNF)-alpha via protein kinase C-delta and TNF-alpha converting enzyme in vascular smooth muscle cells. *Endocrinology* **2009**, *150*, 63–74.
628. Zhang, J.; Duarte, C.G.; Ellis, S. Contrast medium- and mannitol-induced apoptosis in heart and kidney of SHR rats. *Toxicol. Pathol.* **1999**, *27*, 427–435.
629. Kwak, H.J.; Lee, S.J.; Lee, Y.H.; Ryu, C.H.; Koh, K.N.; Choi, H.Y.; Koh, G.Y. Angiopoietin-1 inhibits irradiation- and mannitol-induced apoptosis in endothelial cells. *Circulation* **2000**, *101*, 2317–2324.
630. Orlov, S.N.; Dam, T.V.; Tremblay, J.; Hamet, P. Apoptosis in vascular smooth muscle cells: Role of cell shrinkage. *Biochem. Biophys. Res. Commun.* **1996**, *221*, 708–715.
631. Shi, L.; Ji, Y.; Liu, D.; Liu, Y.; Xu, Y.; Cao, Y.; Jiang, X.; Xu, C. Sitagliptin attenuates high glucose-induced alterations in migration, proliferation, calcification and apoptosis of vascular smooth muscle cells through ERK1/2 signal pathway. *Oncotarget* **2017**, *8*, 77168–77180.
632. Ramana, K.V.; Tammali, R.; Reddy, A.B.M.; Bhatnagar, A.; Srivastava, S.K. Aldose reductase-regulated tumor necrosis factor-alpha production is essential for high glucose-induced vascular smooth muscle cell growth. *Endocrinology* **2007**, *148*, 4371–4384.
633. Alesutan, I.; Musculus, K.; Castor, T.; Alzoubi, K.; Voelkl, J.; Lang, F. Inhibition of Phosphate-Induced Vascular Smooth Muscle Cell Osteo-/Chondrogenic Signaling and Calcification by Bafilomycin A1 and Methylamine. *Kidney Blood Press. Res.* **2015**, *40*, 490–499.
634. Hernandez, M.; Solé, M.; Boada, M.; Unzeta, M. Soluble semicarbazide sensitive amine oxidase (SSAO) catalysis induces apoptosis in vascular smooth muscle cells. *Biochim. Biophys. Acta* **2006**, *1763*, 164–173.
635. Hoffmann, G.; Frede, S.; Kenn, S.; Smolny, M.; Wachter, H.; Fuchs, D.; Grote, J.; Rieder, J.; Schobersberger, W. Neopterin-induced tumor necrosis factor-alpha synthesis in vascular smooth muscle cells in vitro. *Int. Arch. Allergy Immunol.* **1998**, *116*, 240–245.
636. Hoffmann, G.; Kenn, S.; Wirleitner, B.; Deetjen, C.; Frede, S.; Smolny, M.; Rieder, J.; Fuchs, D.; Baier-Bitterlich, G.; Schobersberger, W. Neopterin induces nitric oxide-dependent apoptosis in rat vascular smooth muscle cells. *Immunobiology* **1998**, *199*, 63–73.
637. Hoffmann, G.; Schobersberger, W.; Frede, S.; Pelzer, L.; Fandrey, J.; Wachter, H.; Fuchs, D.; Grote, J. Neopterin activates transcription factor nuclear factor-kappa B in vascular smooth muscle cells. *FEBS Lett.* **1996**, *391*, 181–184.
638. Shirai, R.; Sato, K.; Yamashita, T.; Yamaguchi, M.; Okano, T.; Watanabe-Kominato, K.; Watanabe, R.; Matsuyama, T.-A.; Ishibashi-Ueda, H.; Koba, S.; et al. Neopterin Counters Vascular Inflammation and Atherosclerosis. *J. Am. Heart Assoc.* **2018**, *7*.
639. Cui, Y.; An, S.; Jabr, S.; Maturana, J.A.; Wu, J.M.; Gutstein, W.H. The anti-proliferative effects of nicotinamide and 3-aminobenzamide on human smooth muscle cells in vitro. *Biochem. Mol. Biol. Int.* **1993**, *31*, 935–944.
640. Hao, W.; Yang, R.; Yang, Y.; Jin, S.; Li, Y.; Yuan, F.; Guo, Q.; Xiao, L.; Wang, X.; Wang, F.; et al. Stellate ganglion block ameliorates vascular calcification by inhibiting endoplasmic reticulum stress. *Life Sci.* **2018**, *193*, 1–8.
641. Zhang, H.; Faber, J.E. Trophic effect of norepinephrine on arterial intima-media and adventitia is augmented by injury and mediated by different alpha1-adrenoceptor subtypes. *Circ. Res.* **2001**, *89*, 815–822.

642. Liu, S.; Li, Y.; Zhang, Z.; Xie, F.; Xu, Q.; Huang, X.; Huang, J.; Li, C. α1-Adrenergic receptors mediate combined signals initiated by mechanical stretch stress and norepinephrine leading to accelerated mouse vein graft atherosclerosis. *J. Vasc. Surg.* **2013**, *57*, 1645–1656, 1656.e1-3.
643. Mimura, Y.; Kobayashi, S.; Notoya, K.; Okabe, M.; Kimura, I.; Horikoshi, I.; Kimura, M. Activation by alpha 1-adrenergic agonists of the progression phase in the proliferation of primary cultures of smooth muscle cells in mouse and rat aorta. *Biol. Pharm. Bull.* **1995**, *18*, 1373–1376.
644. Li, Z.; Yu, C.; Han, Y.; Ren, H.; Shi, W.; Fu, C.; He, D.; Huang, L.; Yang, C.; Wang, X.; et al. Inhibitory effect of D1-like and D3 dopamine receptors on norepinephrine-induced proliferation in vascular smooth muscle cells. *Am. J. Physiol. Heart Circ. Physiol.* **2008**, *294*, H2761–H2768.
645. Parmentier, J.-H.; Smelcer, P.; Pavicevic, Z.; Basic, E.; Idrizovic, A.; Estes, A.; Malik, K.U. PKC-zeta mediates norepinephrine-induced phospholipase D activation and cell proliferation in VSMC. *Hypertens. Dallas Tex 1979* **2003**, *41*, 794–800.
646. Wen, Y.Y.; Wang, S.X.; Chen, M.Q. Studies on age-related changes in vascular smooth muscle and their Ca2+ mechanisms. *Clin. Exp. Pharmacol. Physiol.* **1999**, *26*, 840–841.
647. Jiao, L.; Wang, M.-C.; Yang, Y.-A.; Chen, E.-Q.; Xu, H.-T.; Wu, K.-Y.; Zhang, S.-M. Norepinephrine reversibly regulates the proliferation and phenotypic transformation of vascular smooth muscle cells. *Exp. Mol. Pathol.* **2008**, *85*, 196–200.
648. Bell, L.; Madri, J.A. Effect of platelet factors on migration of cultured bovine aortic endothelial and smooth muscle cells. *Circ. Res.* **1989**, *65*, 1057–1065.
649. Luo, Q.; Wang, X.; Liu, R.; Qiao, H.; Wang, P.; Jiang, C.; Zhang, Q.; Cao, Y.; Yu, H.; Qu, L. alpha1A-adrenoceptor is involved in norepinephrine-induced proliferation of pulmonary artery smooth muscle cells via CaMKII signaling. *J. Cell. Biochem.* **2019**, *120*, 9345–9355.
650. Yu, C.; Wang, Z.; Han, Y.; Liu, Y.; Wang, W.E.; Chen, C.; Wang, H.; Jose, P.A.; Zeng, C. Dopamine D_4 receptors inhibit proliferation and migration of vascular smooth muscle cells induced by insulin via down-regulation of insulin receptor expression. *Cardiovasc. Diabetol.* **2014**, *13*, 97.
651. Liu, R.; Zhang, Q.; Luo, Q.; Qiao, H.; Wang, P.; Yu, J.; Cao, Y.; Lu, B.; Qu, L. Norepinephrine stimulation of alpha1D-adrenoceptor promotes proliferation of pulmonary artery smooth muscle cells via ERK-1/2 signaling. *Int. J. Biochem. Cell Biol.* **2017**, *88*, 100–112.
652. Düsing, R.; Göbel, B.; Weisser, B.; Dittrich, D.; Kraemer, S.; Vetter, H. [Mechanism and significance of arteriolar media hypertrophy/ hyperplasia in arterial hypertension. Role of the Na+/H+ antiport]. *Klin. Wochenschr.* **1988**, *66*, 1151–1159.
653. Shimamura, N.; Ohkuma, H. Phenotypic transformation of smooth muscle in vasospasm after aneurysmal subarachnoid hemorrhage. *Transl. Stroke Res.* **2014**, *5*, 357–364.
654. Zhang, H.; Facemire, C.S.; Banes, A.J.; Faber, J.E. Different alpha-adrenoceptors mediate migration of vascular smooth muscle cells and adventitial fibroblasts in vitro. *Am. J. Physiol. Heart Circ. Physiol.* **2002**, *282*, H2364–H2370.
655. Turner, J.L.; Bierman, E.L. Effects of glucose and sorbitol on proliferation of cultured human skin fibroblasts and arterial smooth-muscle cells. *Diabetes* **1978**, *27*, 583–588.
656. Zhang, X.; Li, Y.; Yang, P.; Liu, X.; Lu, L.; Chen, Y.; Zhong, X.; Li, Z.; Liu, H.; Ou, C.; et al. Trimethylamine-N-Oxide Promotes Vascular Calcification Through Activation of NLRP3 (Nucleotide-Binding Domain, Leucine-Rich-Containing Family, Pyrin Domain-Containing-3) Inflammasome and NF-κB (Nuclear Factor κB) Signals. *Arterioscler. Thromb. Vasc. Biol.* **2020**, *40*, 751–765.
657. Meyer, K.A.; Benton, T.Z.; Bennett, B.J.; Jacobs, D.R.; Lloyd-Jones, D.M.; Gross, M.D.; Carr, J.J.; Gordon-Larsen, P.; Zeisel, S.H. Microbiota-Dependent Metabolite Trimethylamine N-Oxide and Coronary Artery Calcium in the Coronary Artery Risk Development in Young Adults Study (CARDIA). *J. Am. Heart Assoc.* **2016**, *5*.
658. Seldin, M.M.; Meng, Y.; Qi, H.; Zhu, W.; Wang, Z.; Hazen, S.L.; Lusis, A.J.; Shih, D.M. Trimethylamine N-Oxide Promotes Vascular Inflammation Through Signaling of Mitogen-Activated Protein Kinase and Nuclear Factor-κB. *J. Am. Heart Assoc.* **2016**, *5*.
659. Lau, W.L.; Vaziri, N.D. Urea, a true uremic toxin: The empire strikes back. *Clin. Sci. Lond. Engl. 1979* **2017**, *131*, 3–12.

660. Apostolov, E.O.; Basnakian, A.G.; Ok, E.; Shah, S.V. Carbamylated low-density lipoprotein: Nontraditional risk factor for cardiovascular events in patients with chronic kidney disease. *J. Ren. Nutr. Off. J. Counc. Ren. Nutr. Natl. Kidney Found.* **2012**, *22*, 134–138.
661. Song, Z.; Zhao, Y.; Wang, X.; Xu, M.-J. [Secondary hyperuricemia in chronic renal failure promotes vascular calcification in rats]. *Sheng Li Xue Bao* **2016**, *68*, 709–715.
662. Yan, B.; Liu, D.; Zhu, J.; Pang, X. The effects of hyperuricemia on the differentiation and proliferation of osteoblasts and vascular smooth muscle cells are implicated in the elevated risk of osteopenia and vascular calcification in gout: An in vivo and in vitro analysis. *J. Cell. Biochem.* **2019**, *120*, 19660–19672.
663. Jun, J.E.; Lee, Y.-B.; Lee, S.-E.; Ahn, J.Y.; Kim, G.; Jin, S.-M.; Hur, K.Y.; Lee, M.-K.; Kang, M.R.; Kim, J.H. Elevated serum uric acid predicts the development of moderate coronary artery calcification independent of conventional cardiovascular risk factors. *Atherosclerosis* **2018**, *272*, 233–239.
664. Grossman, C.; Shemesh, J.; Koren-Morag, N.; Bornstein, G.; Ben-Zvi, I.; Grossman, E. Serum uric acid is associated with coronary artery calcification. *J. Clin. Hypertens. Greenwich Conn* **2014**, *16*, 424–428.
665. Calvo, R.Y.; Araneta, M.R.G.; Kritz-Silverstein, D.; Laughlin, G.A.; Barrett-Connor, E. Relation of serum uric acid to severity and progression of coronary artery calcium in postmenopausal White and Filipino women (from the Rancho Bernardo study). *Am. J. Cardiol.* **2014**, *113*, 1153–1158.
666. D'Marco, L.; García, I.; Vega, C. [Uric acid, atherosclerosis and vascular calcifications in chronic kidney disease]. *Invest. Clin.* **2012**, *53*, 52–59.
667. Malik, R.; Aneni, E.C.; Shahrayar, S.; Freitas, W.M.; Ali, S.S.; Veledar, E.; Latif, M.A.; Aziz, M.; Ahmed, R.; Khan, S.A.; et al. Elevated serum uric acid is associated with vascular inflammation but not coronary artery calcification in the healthy octogenarians: The Brazilian study on healthy aging. *Aging Clin. Exp. Res.* **2016**, *28*, 359–362.
668. Corry, D.B.; Eslami, P.; Yamamoto, K.; Nyby, M.D.; Makino, H.; Tuck, M.L. Uric acid stimulates vascular smooth muscle cell proliferation and oxidative stress via the vascular renin-angiotensin system. *J. Hypertens.* **2008**, *26*, 269–275.
669. Tang, L.; Xu, Y.; Wei, Y.; He, X. Uric acid induces the expression of TNF-α via the ROS-MAPK-NF-κB signaling pathway in rat vascular smooth muscle cells. *Mol. Med. Rep.* **2017**, *16*, 6928–6933.
670. Oğuz, N.; Kırça, M.; Çetin, A.; Yeşilkaya, A. Effect of uric acid on inflammatory COX-2 and ROS pathways in vascular smooth muscle cells. *J. Recept. Signal Transduct. Res.* **2017**, *37*, 500–505.
671. Dai, Y.; Cao, Y.; Zhang, Z.; Vallurupalli, S.; Mehta, J.L. Xanthine Oxidase Induces Foam Cell Formation through LOX-1 and NLRP3 Activation. *Cardiovasc. Drugs Ther.* **2017**, *31*, 19–27.
672. Kang, D.-H.; Han, L.; Ouyang, X.; Kahn, A.M.; Kanellis, J.; Li, P.; Feng, L.; Nakagawa, T.; Watanabe, S.; Hosoyamada, M.; et al. Uric acid causes vascular smooth muscle cell proliferation by entering cells via a functional urate transporter. *Am. J. Nephrol.* **2005**, *25*, 425–433.
673. Kang, D.-H.; Park, S.-K.; Lee, I.-K.; Johnson, R.J. Uric acid-induced C-reactive protein expression: Implication on cell proliferation and nitric oxide production of human vascular cells. *J. Am. Soc. Nephrol. JASN* **2005**, *16*, 3553–3562.
674. Kanellis, J.; Watanabe, S.; Li, J.H.; Kang, D.H.; Li, P.; Nakagawa, T.; Wamsley, A.; Sheikh-Hamad, D.; Lan, H.Y.; Feng, L.; et al. Uric acid stimulates monocyte chemoattractant protein-1 production in vascular smooth muscle cells via mitogen-activated protein kinase and cyclooxygenase-2. *Hypertens. Dallas Tex 1979* **2003**, *41*, 1287–1293.
675. Fu, X.; Niu, N.; Li, G.; Xu, M.; Lou, Y.; Mei, J.; Liu, Q.; Sui, Z.; Sun, J.; Qu, P. Blockage of macrophage migration inhibitory factor (MIF) suppressed uric acid-induced vascular inflammation, smooth muscle cell de-differentiation, and remodeling. *Biochem. Biophys. Res. Commun.* **2019**, *508*, 440–444.
676. Kang, D.-H.; Nakagawa, T.; Feng, L.; Watanabe, S.; Han, L.; Mazzali, M.; Truong, L.; Harris, R.; Johnson, R.J. A role for uric acid in the progression of renal disease. *J. Am. Soc. Nephrol. JASN* **2002**, *13*, 2888–2897.
677. Yamamoto, Y.; Ogino, K.; Igawa, G.; Matsuura, T.; Kaetsu, Y.; Sugihara, S.; Matsubara, K.; Miake, J.; Hamada, T.; Yoshida, A.; et al. Allopurinol reduces neointimal hyperplasia in the carotid artery ligation model in spontaneously hypertensive rats. *Hypertens. Res. Off. J. Jpn. Soc. Hypertens.* **2006**, *29*, 915–921.
678. Kırça, M.; Oğuz, N.; Çetin, A.; Uzuner, F.; Yeşilkaya, A. Uric acid stimulates proliferative pathways in vascular smooth muscle cells through the activation of p38 MAPK, p44/42 MAPK and PDGFRβ. *J. Recept. Signal Transduct. Res.* **2017**, *37*, 167–173.

679. Nair, R.V.; Cao, W.; Morris, R.E. Inhibition of smooth muscle cell proliferation in vitro by leflunomide, a new immunosuppressant, is antagonized by uridine. *Immunol. Lett.* **1995**, *48*, 77–80.
